# Supplementary material for: Green and Efficient Construction of Chromeno[3,4-c]pyrrole Core via Barton–Zard Reaction from 3-Nitro-2H-chromenes and Ethyl Isocyanoacetate
Source: Molecules. 2022 Dec 2;27(23):8456. doi: 10.3390/molecules27238456 (PMC9737169; doi:10.3390/molecules27238456)
Supplement: Supplementary file 1 [file molecules-27-08456-s001.zip › molecules-2059303-supplementary.pdf]

Supporting information for

Green and efficient construction of chromeno[3,4-*c*]pyrrole core *via* Barton-Zard reaction from 3-nitro-2*H*-chromenes and ethyl isocyanoacetate

Ivan A. Kochnev, Alexey Y. Barkov, Nikolay S. Zimnitskiy, Vladislav Y. Korotaev* and Vyacheslav Y. Sosnovskikh*

Institute of Natural Sciences and Mathematics, Ural Federal University, 51 Lenina Ave., 620000 Ekaterinburg, Russian Federation

E-mail: *korotev.vladislav@urfu.ru*, *vy.sosnovskikh@urfu.ru*

Table of Contents

1. Copies of ^{1}H and ^{13}C NMR spectra of synthesized compounds S2–S32

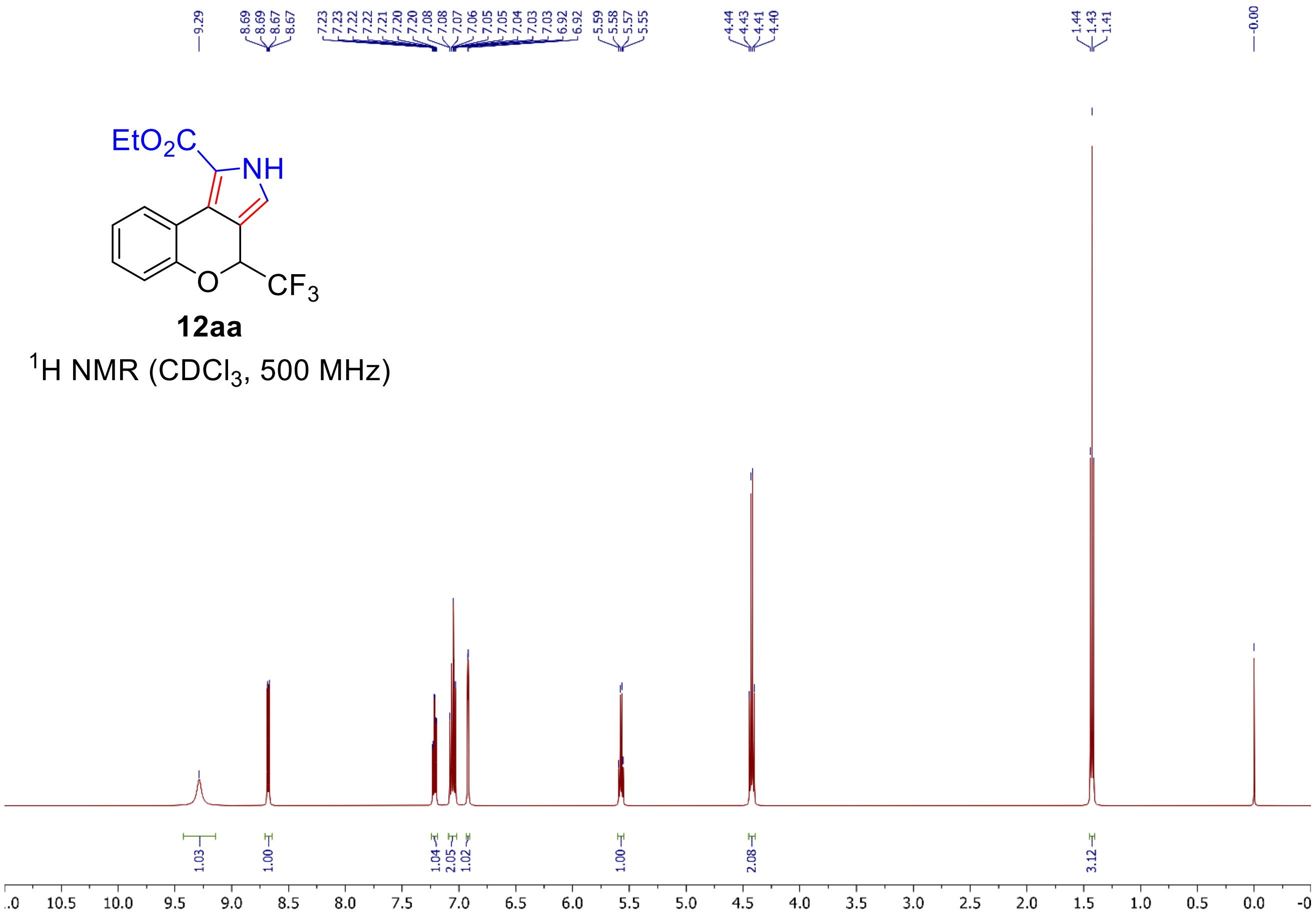
12aa
^{1}H NMR ($CDCl_3$, 500 MHz)
9.29
8.69 8.69 8.67 8.67
7.23 7.23 7.22 7.22 7.21 7.20 7.20 7.08 7.07 7.06 7.05 7.05 7.04 7.03 7.03 6.92 6.92
5.59 5.58 5.57 5.55
4.44 4.43 4.41 4.40
1.44 1.43 1.41
0.00
1.03 1.00 1.04 2.05 1.02 1.00 2.08 3.12

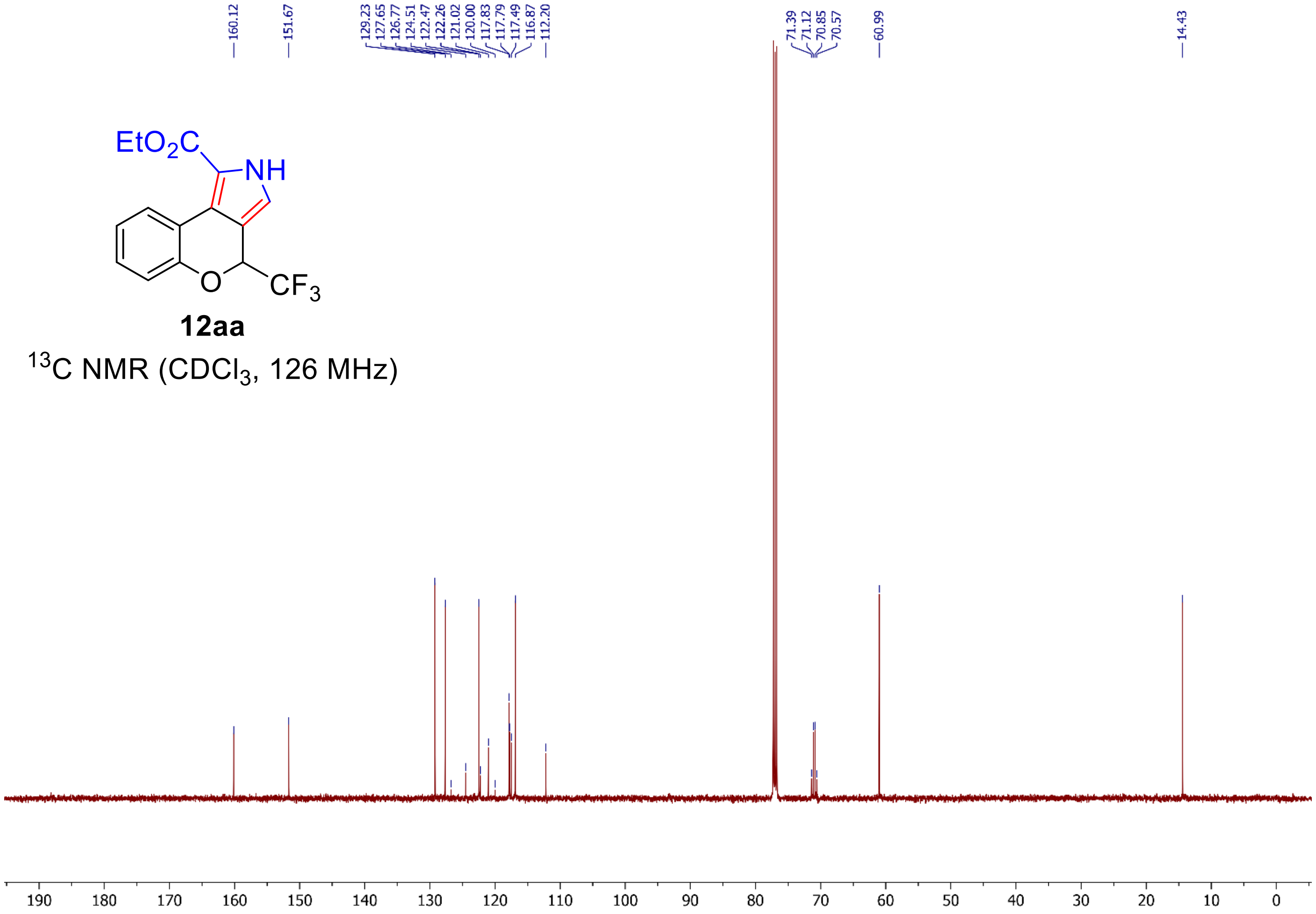
12aa
^{13}C NMR ($CDCl_3$, 126 MHz)
160.12
151.67
129.23 127.65 126.77 124.51 122.47 122.26 121.02 120.00 117.83 117.79 117.49 116.87 112.20
71.39 71.12 70.85 70.57
60.99
14.43

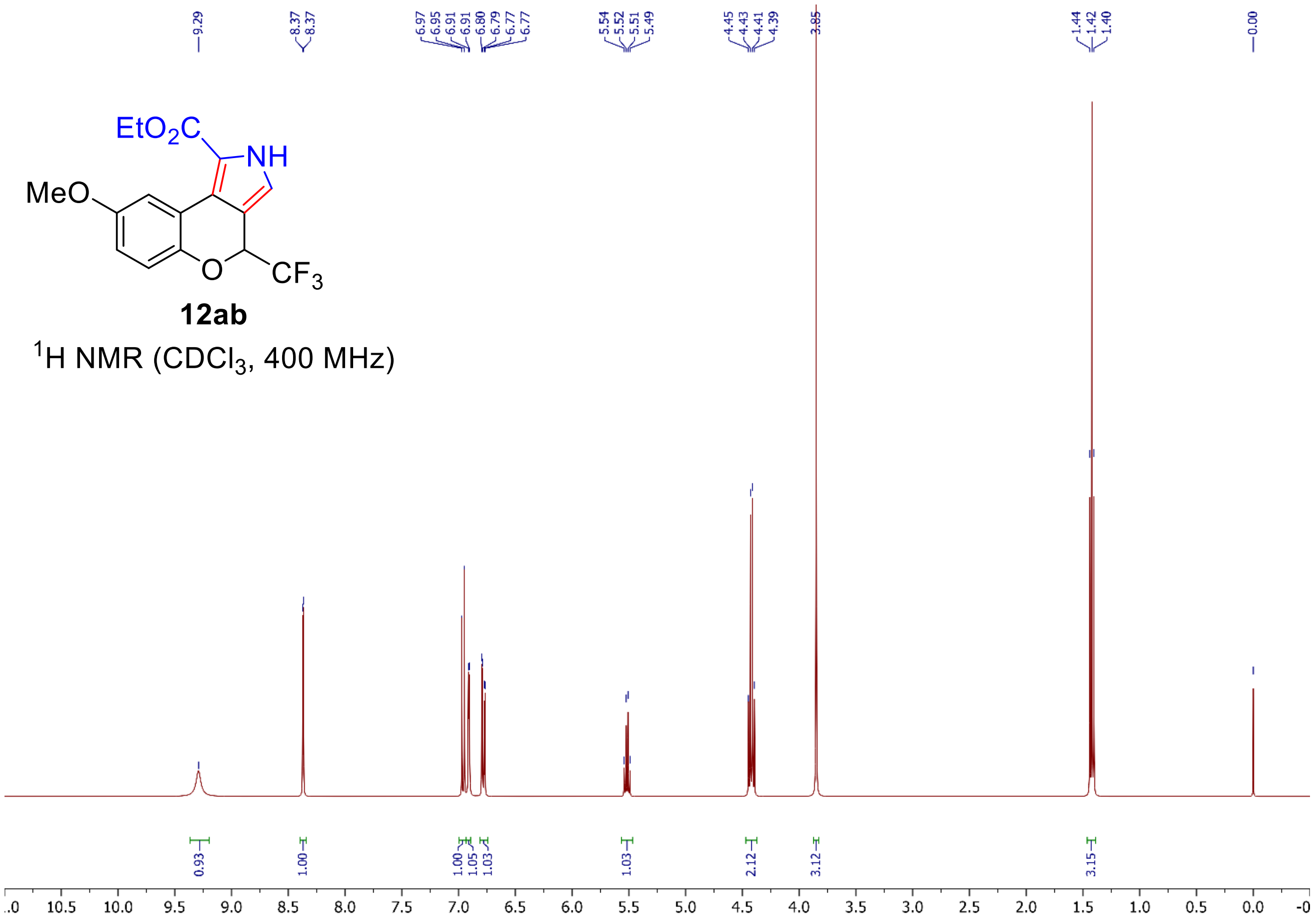

12ab

^{1}H NMR ($CDCl_3$, 400 MHz)

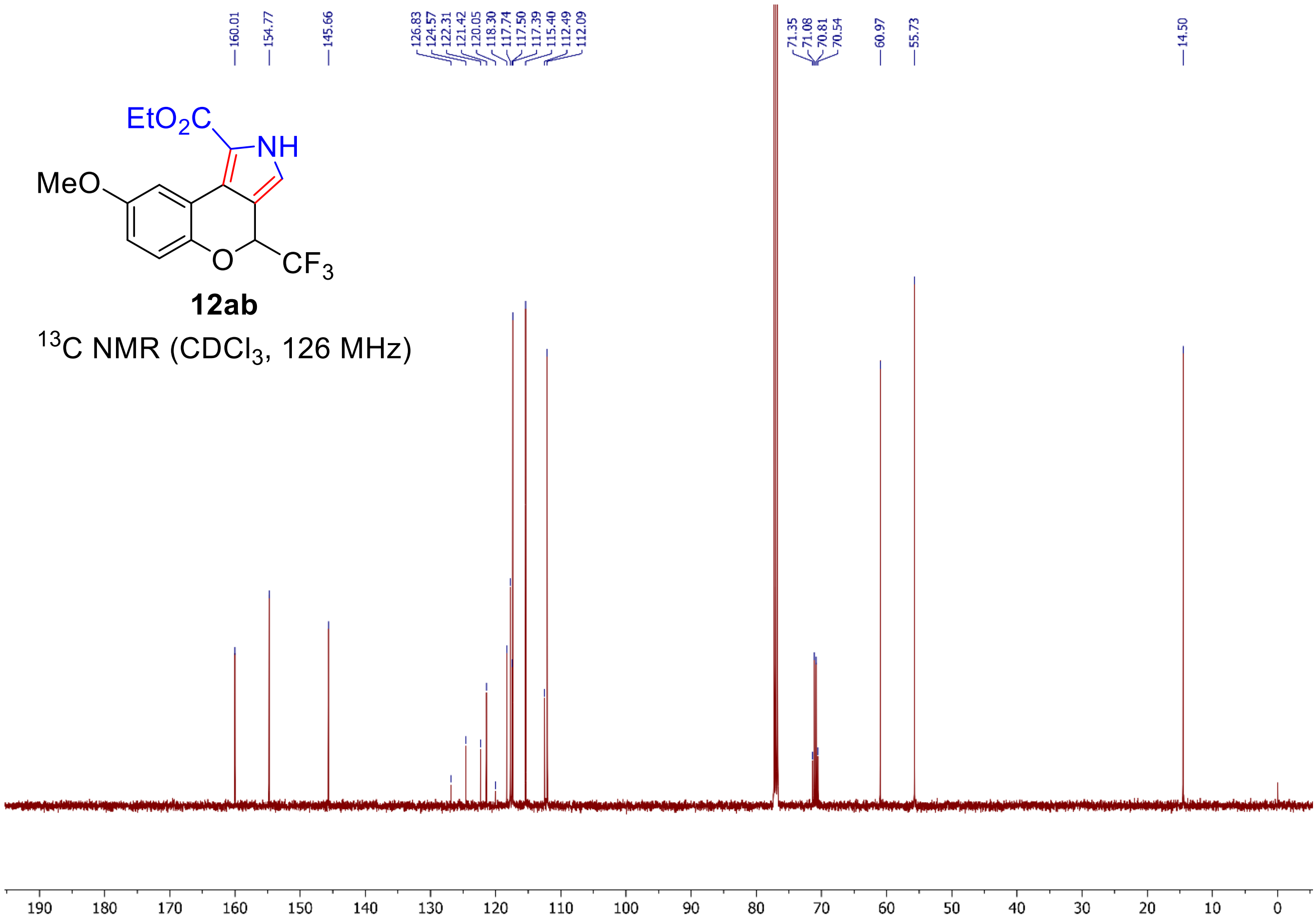

12ab

^{13}C NMR ($CDCl_3$, 126 MHz)

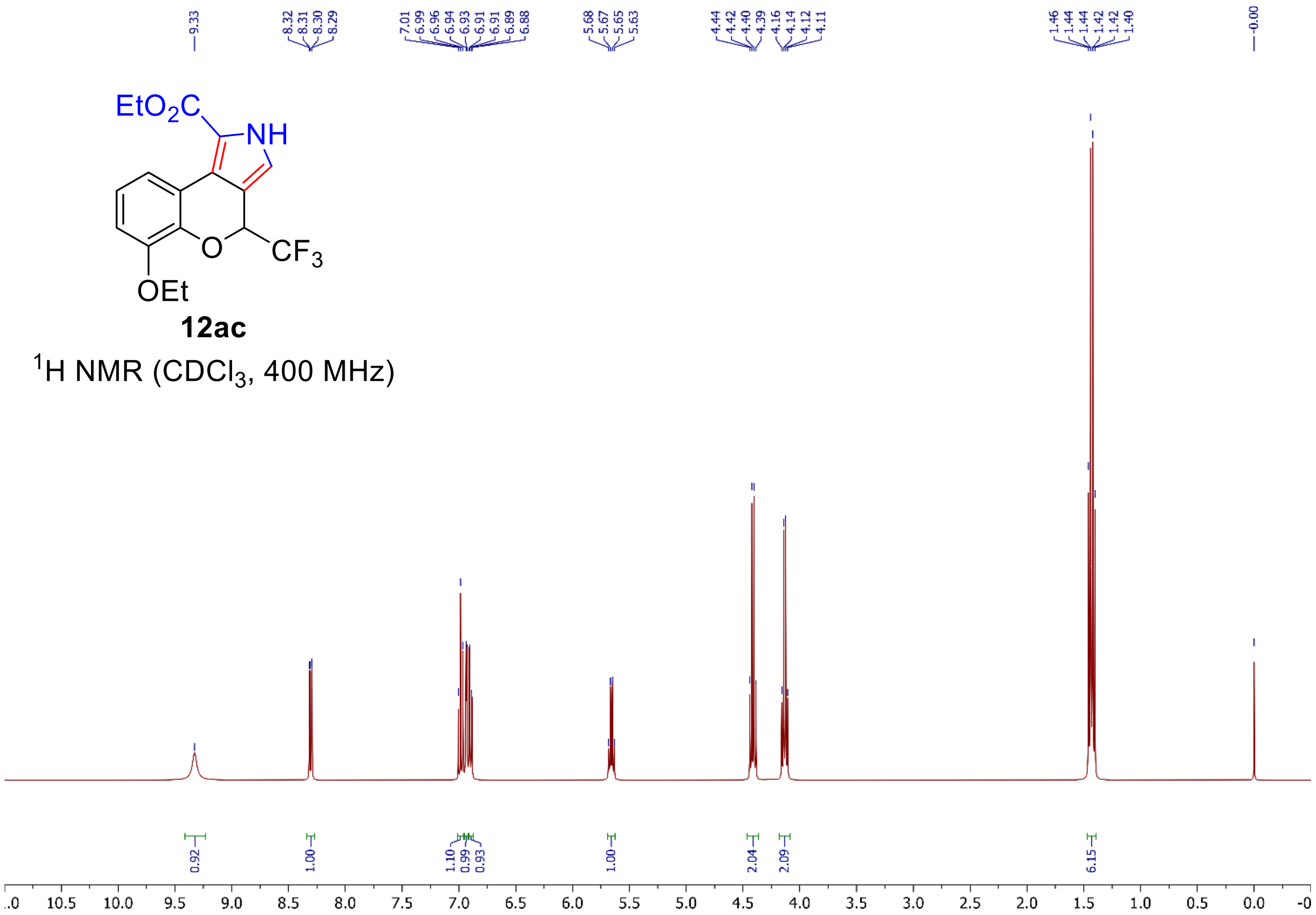

12ac

^{1}H NMR ($CDCl_3$, 400 MHz)

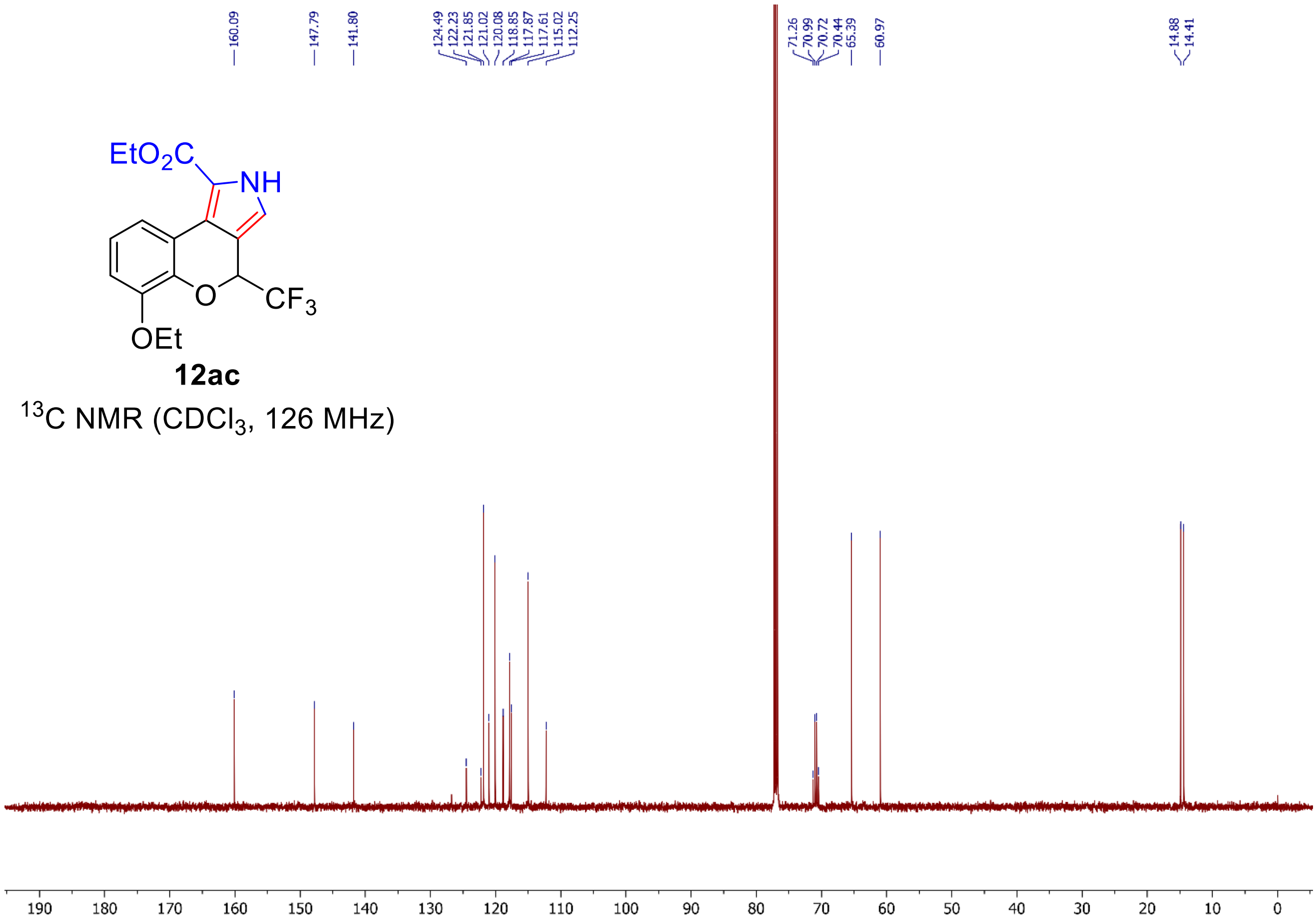

12ac

^{13}C NMR ($CDCl_3$, 126 MHz)

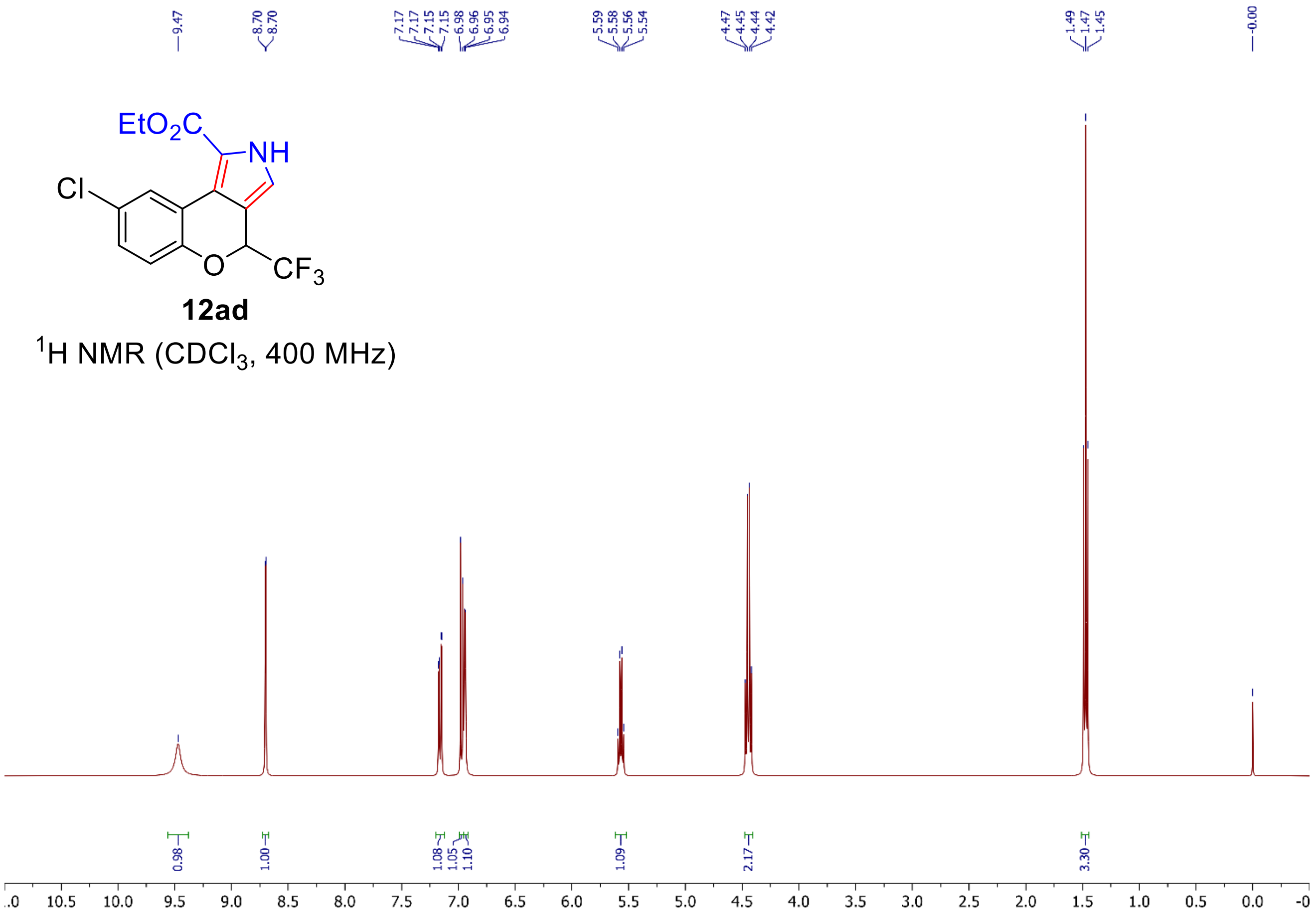
9.47
8.70 8.70
7.17 7.17 7.15 7.15 6.98 6.96 6.95 6.94
5.59 5.58 5.56 5.54
4.47 4.45 4.44 4.42
1.49 1.47 1.45
-0.00
EtO2C
NH
Cl
O
CF3
12ad
1H NMR (CDCl3, 400 MHz)
0.98
1.00
1.08 1.05 1.10
1.09
2.17
3.30

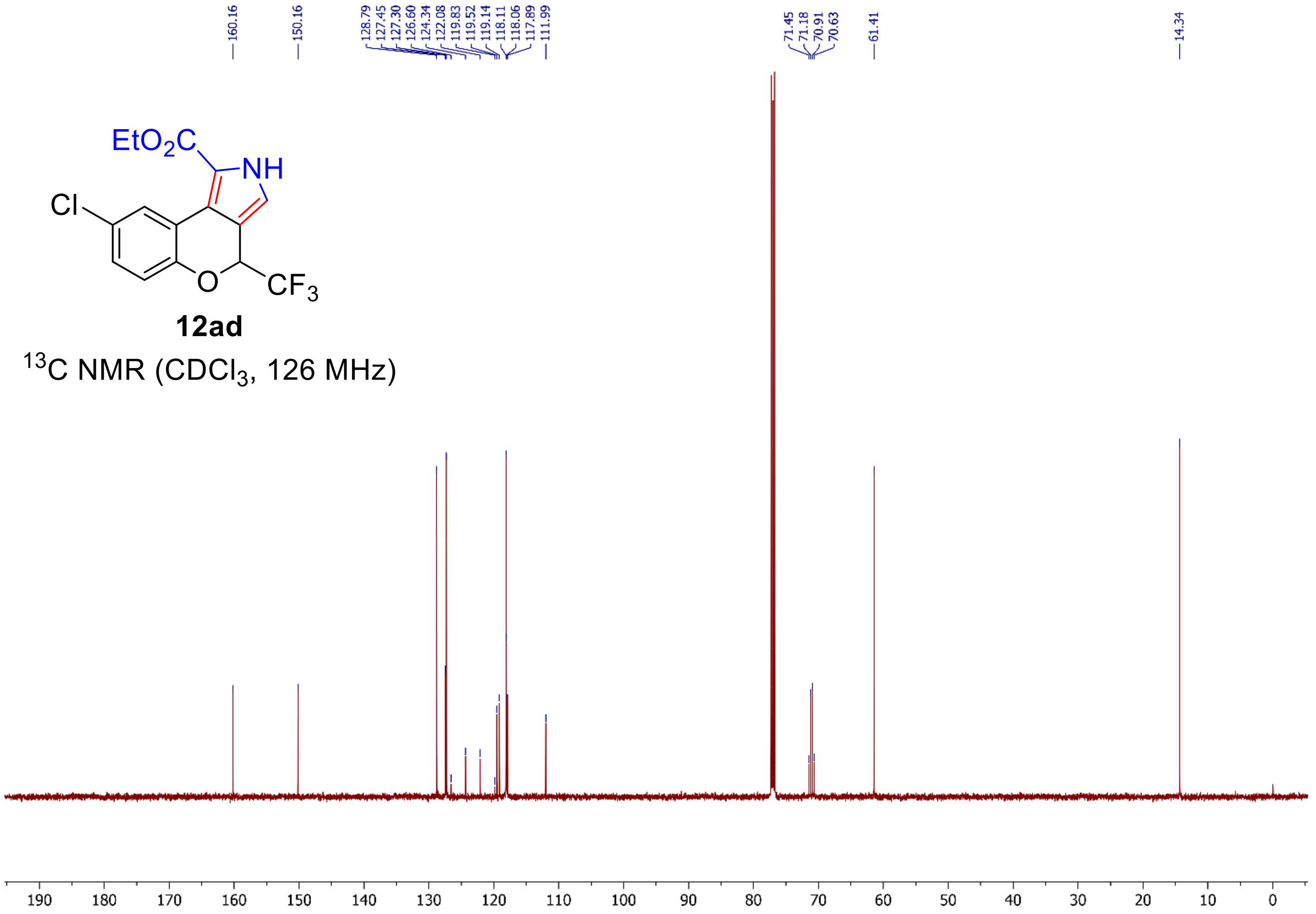
160.16
150.16
128.79 127.45 127.30 126.60 124.34 122.08 119.83 119.52 119.14 118.11 118.06 117.89 111.99
71.45 71.18 70.91 70.63
61.41
14.34
EtO2C
NH
Cl
O
CF3
12ad
13C NMR (CDCl3, 126 MHz)

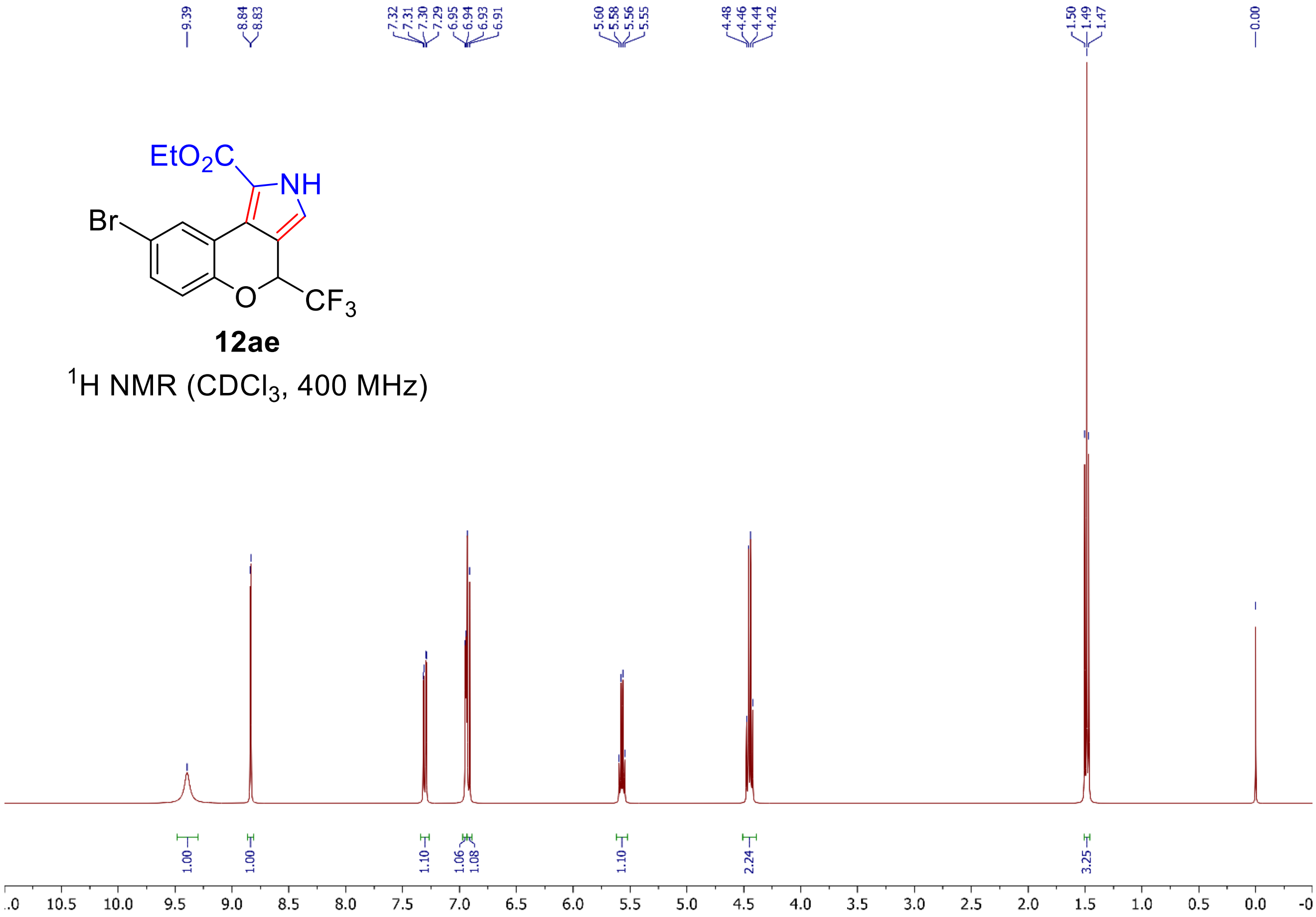

12ae

^{1}H NMR ($CDCl_3$, 400 MHz)

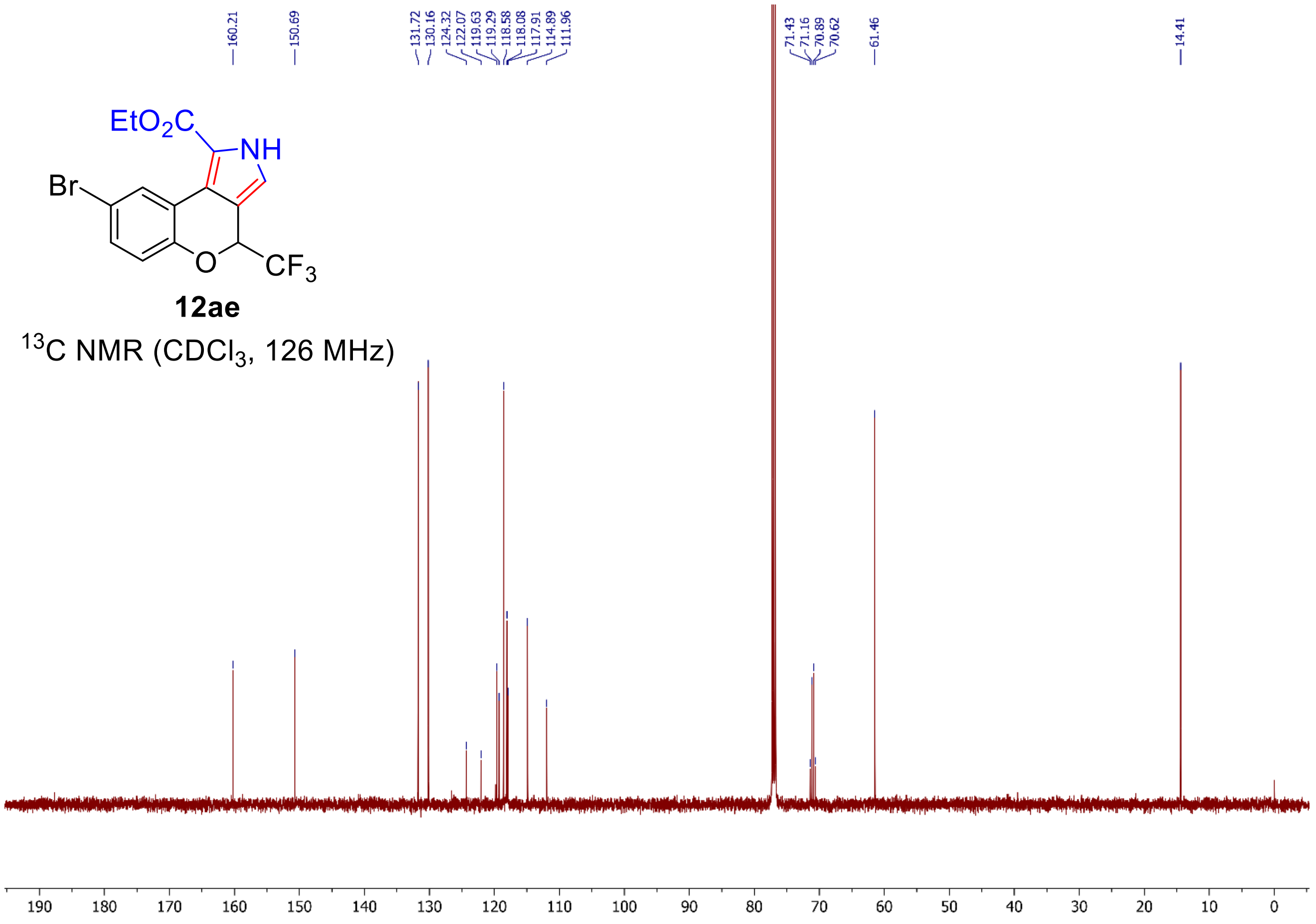

12ae

^{13}C NMR ($CDCl_3$, 126 MHz)

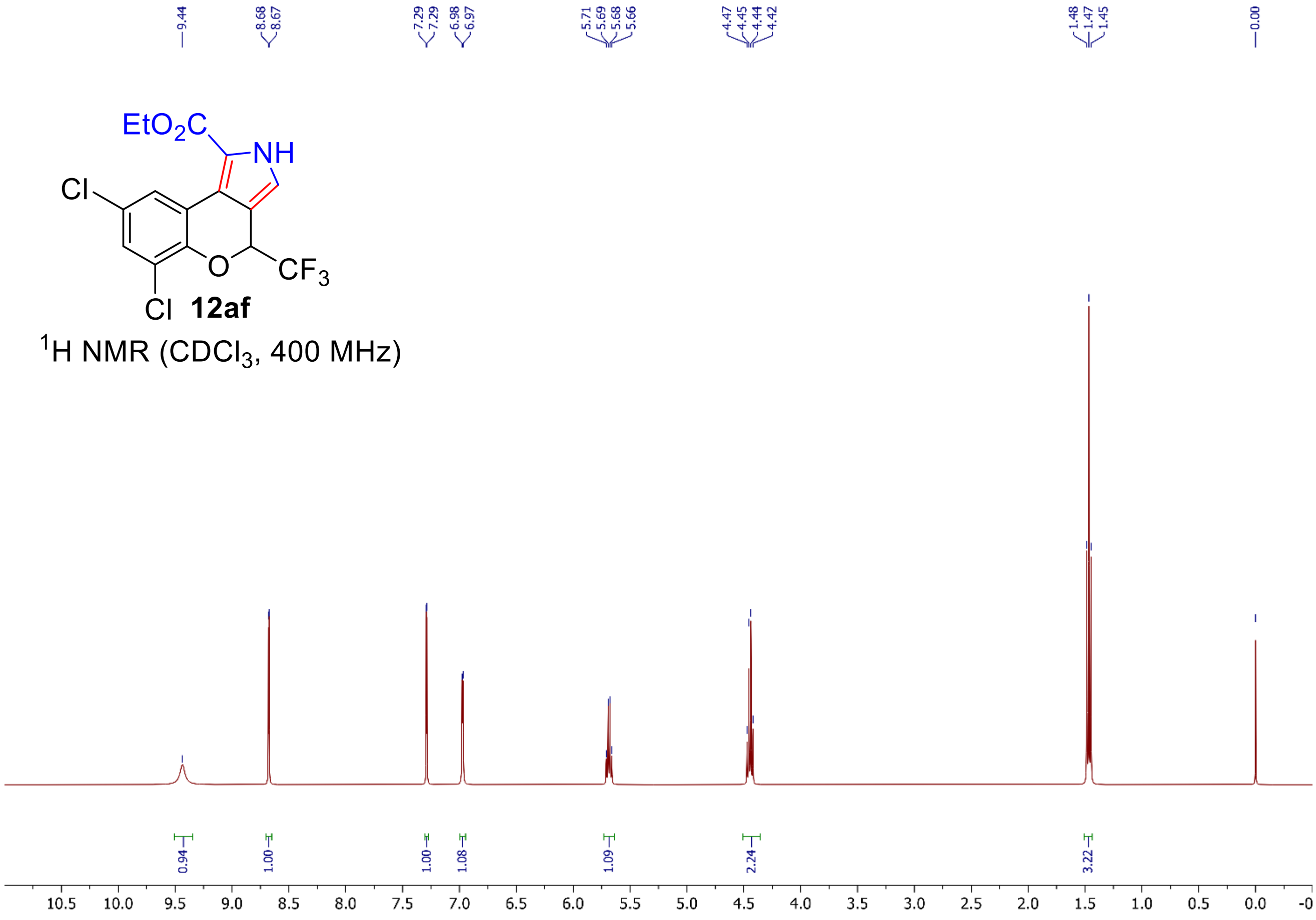
9.44
8.68
8.67
7.29
7.29
6.98
6.97
5.71
5.69
5.68
5.66
4.47
4.45
4.44
4.42
1.48
1.47
1.45
0.00
EtO_2C
NH
Cl
Cl
O
CF_3
12af
^{1}H NMR ($CDCl_3$, 400 MHz)
0.94
1.00
1.00
1.08
1.09
2.24
3.22
10.5 10.0 9.5 9.0 8.5 8.0 7.5 7.0 6.5 6.0 5.5 5.0 4.5 4.0 3.5 3.0 2.5 2.0 1.5 1.0 0.5 0.0 -0

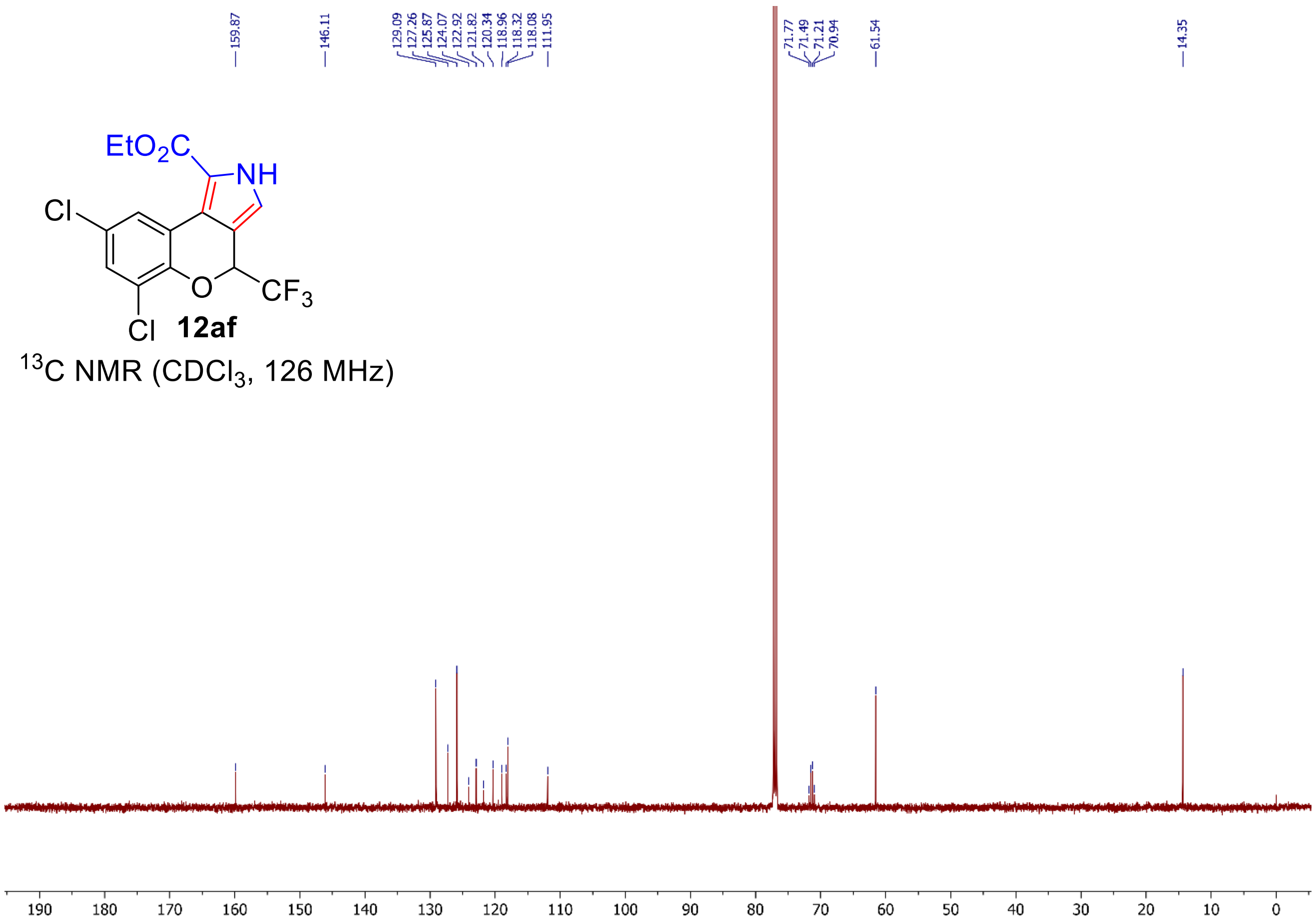
159.87
146.11
129.09
127.26
125.87
124.07
122.92
121.82
120.34
118.96
118.32
118.08
111.95
71.77
71.49
71.21
70.94
61.54
14.35
EtO_2C
NH
Cl
Cl
O
CF_3
12af
^{13}C NMR ($CDCl_3$, 126 MHz)
190 180 170 160 150 140 130 120 110 100 90 80 70 60 50 40 30 20 10 0

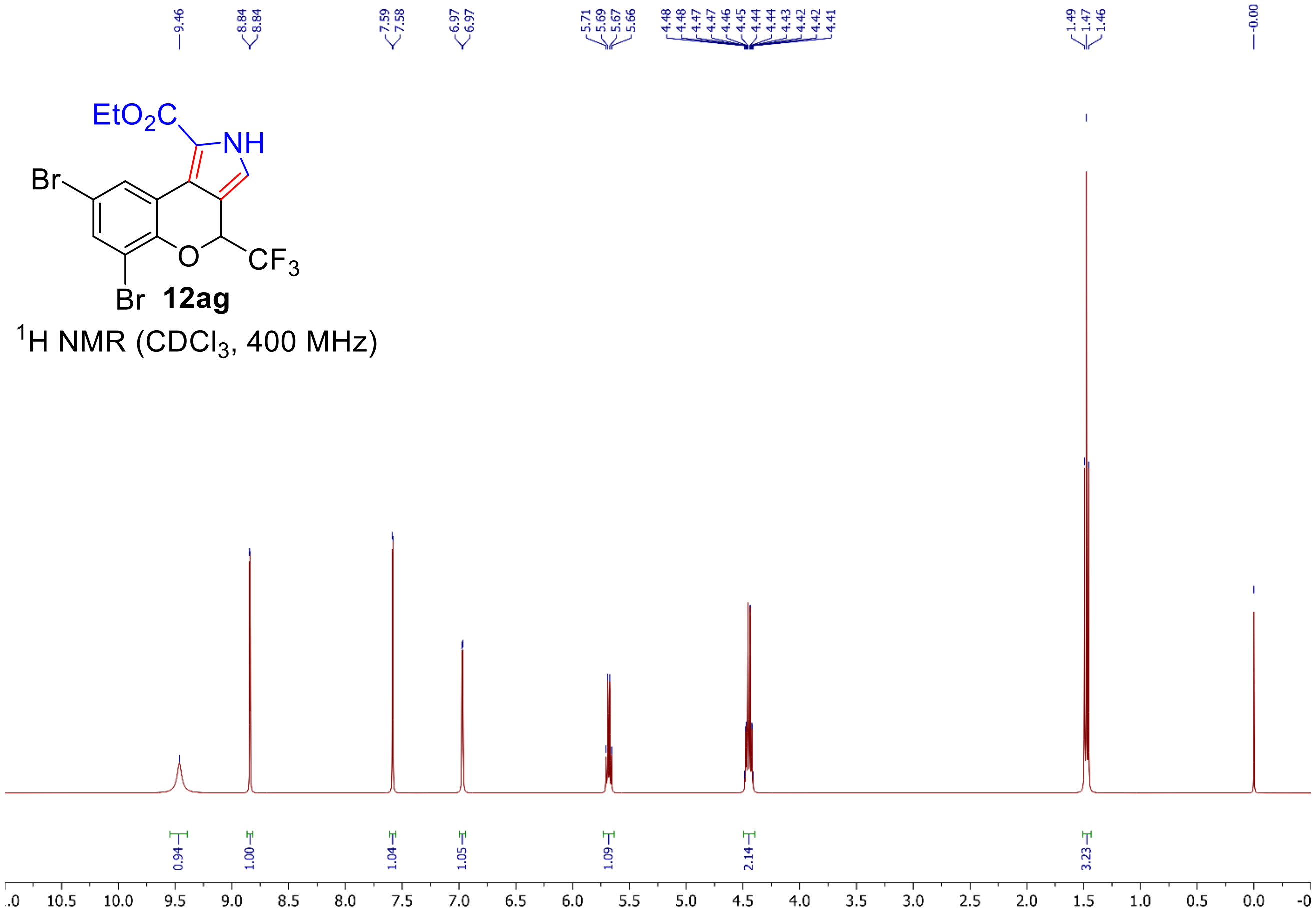

12ag

^{1}H NMR ($CDCl_3$, 400 MHz)

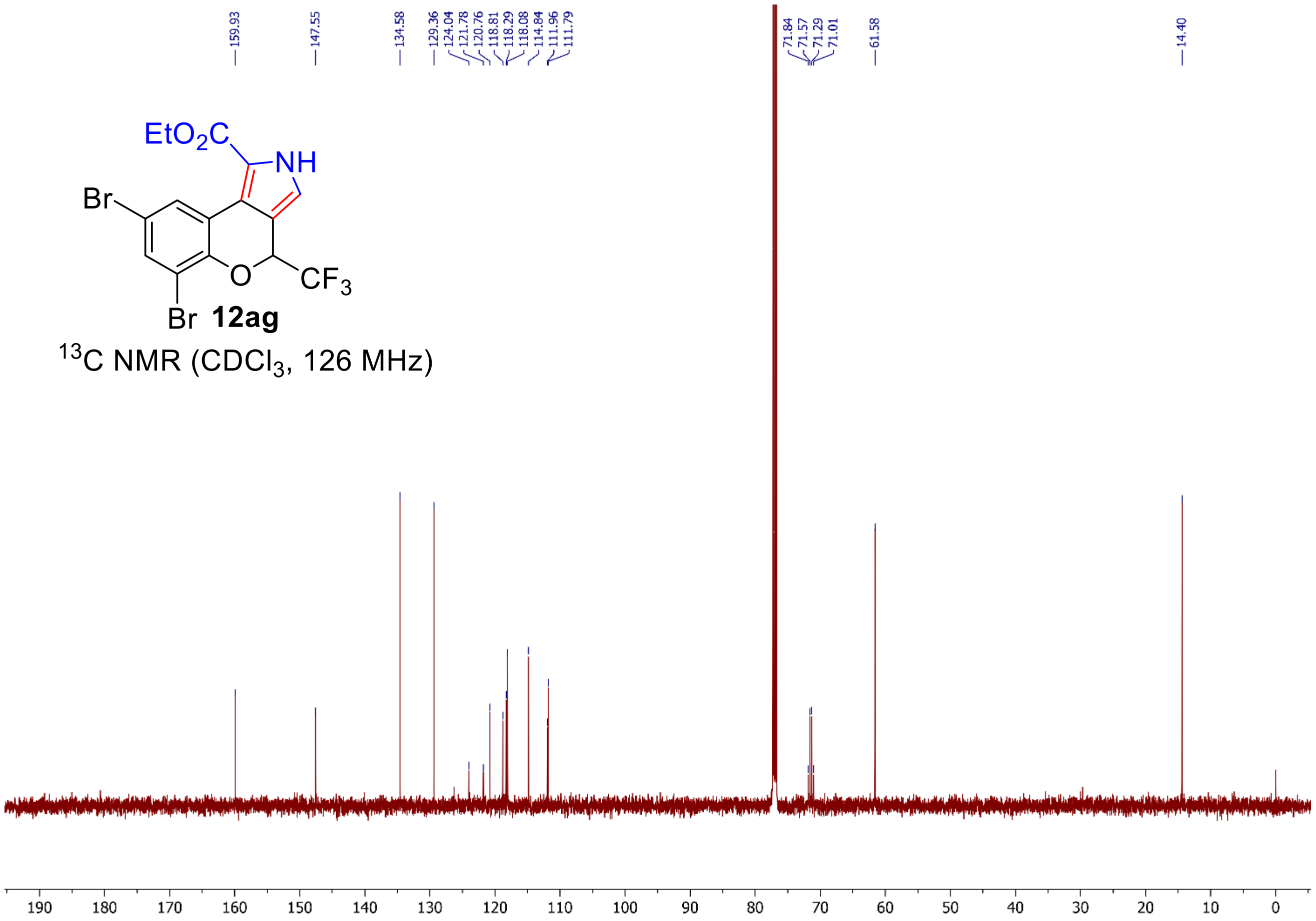

12ag

^{13}C NMR ($CDCl_3$, 126 MHz)

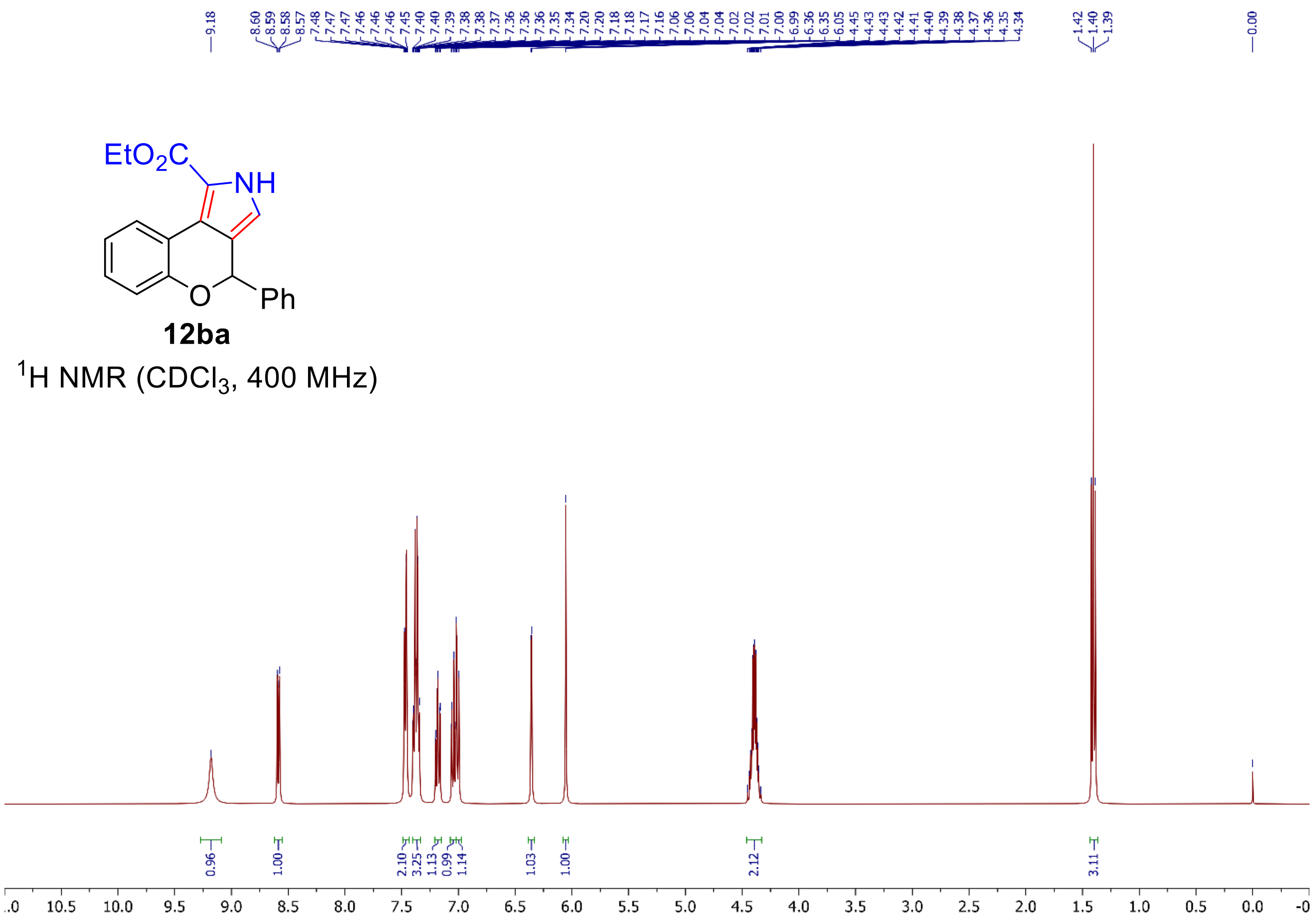
EtO2C
NH
O
Ph
12ba
1H NMR (CDCl3, 400 MHz)

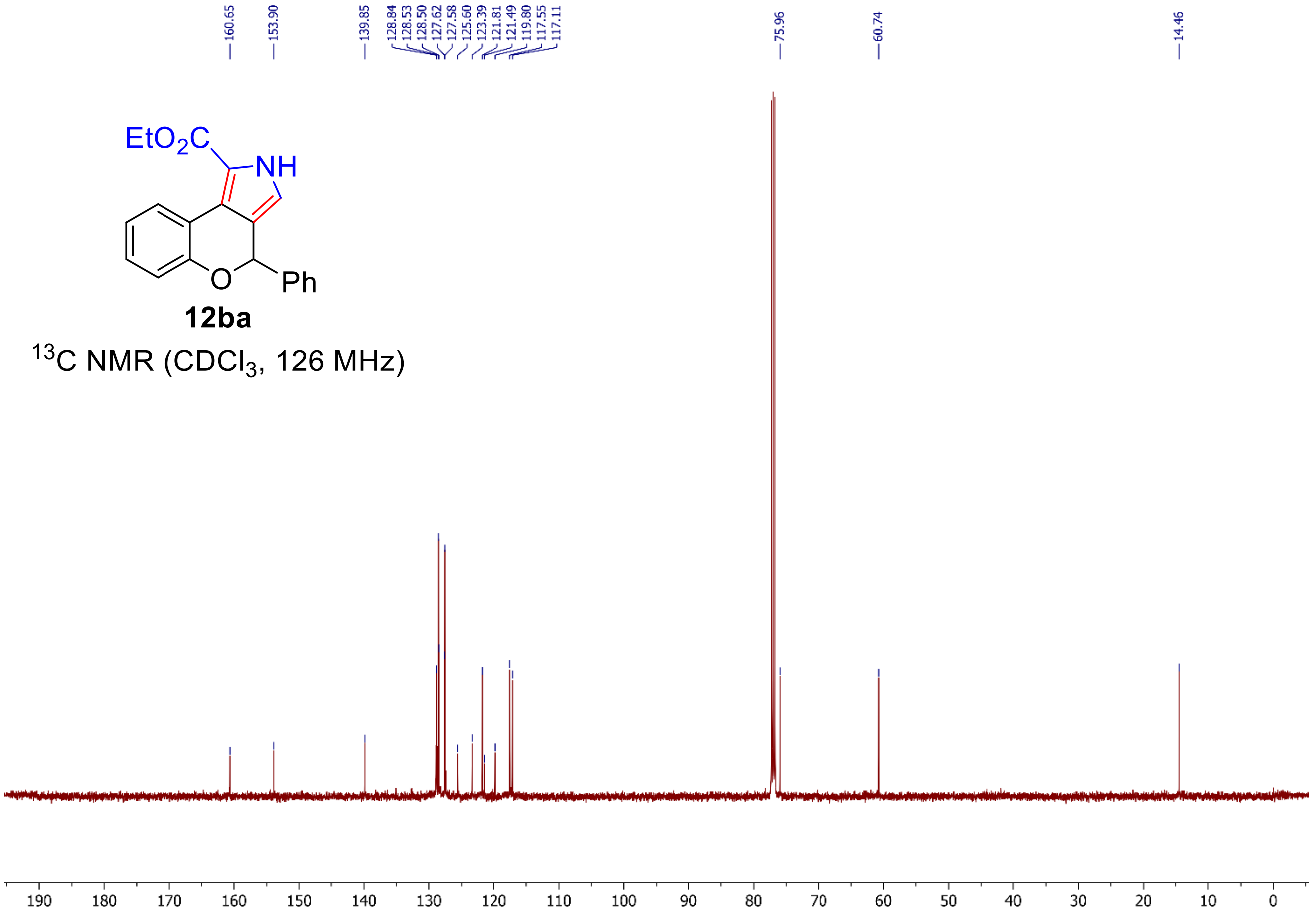
EtO2C
NH
O
Ph
12ba
13C NMR (CDCl3, 126 MHz)

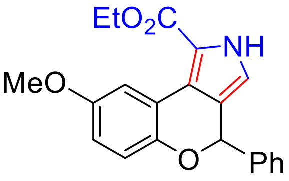

12bb

^{1}H NMR ($CDCl_3$, 400 MHz)

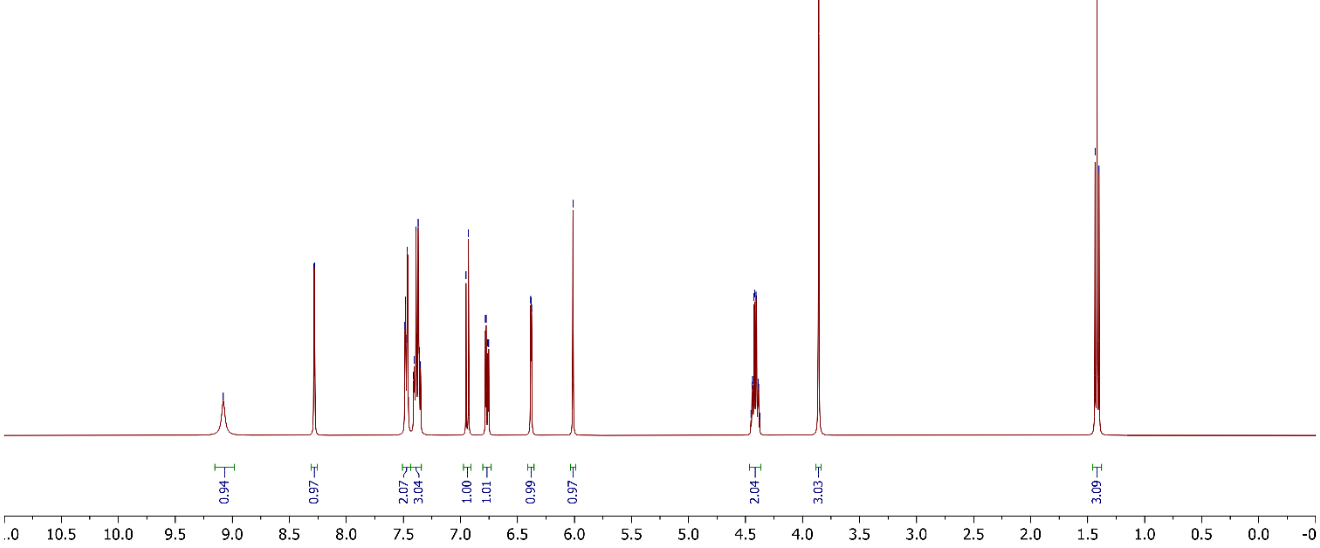

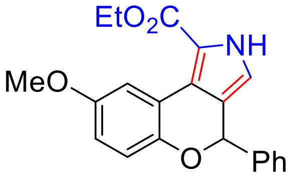

12bb

^{13}C NMR ($CDCl_3$, 126 MHz)

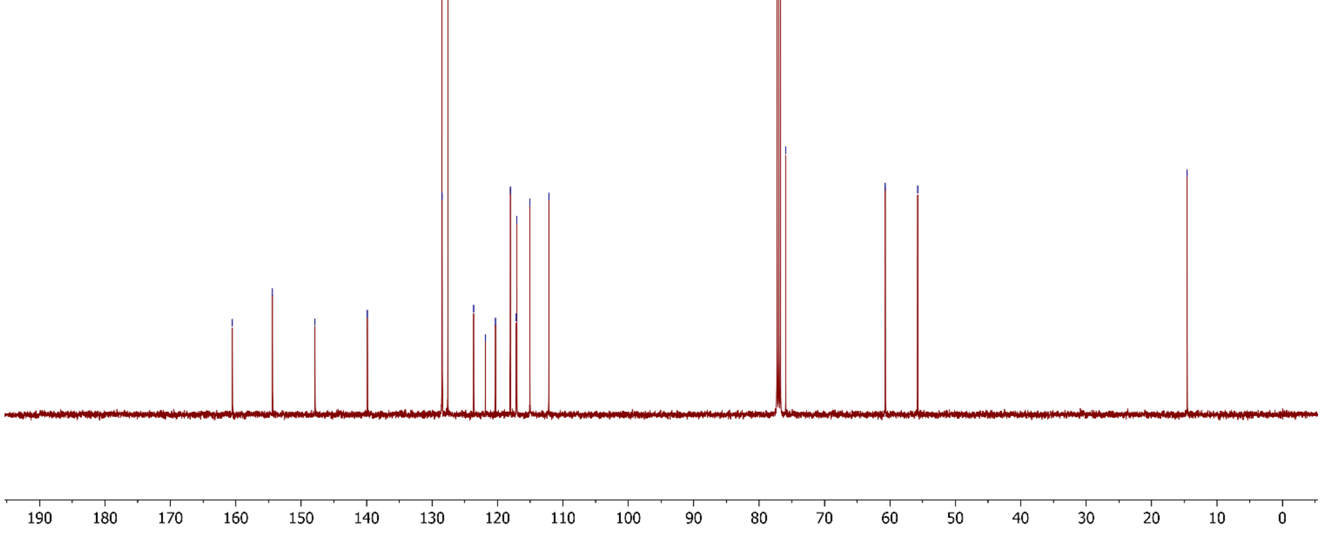

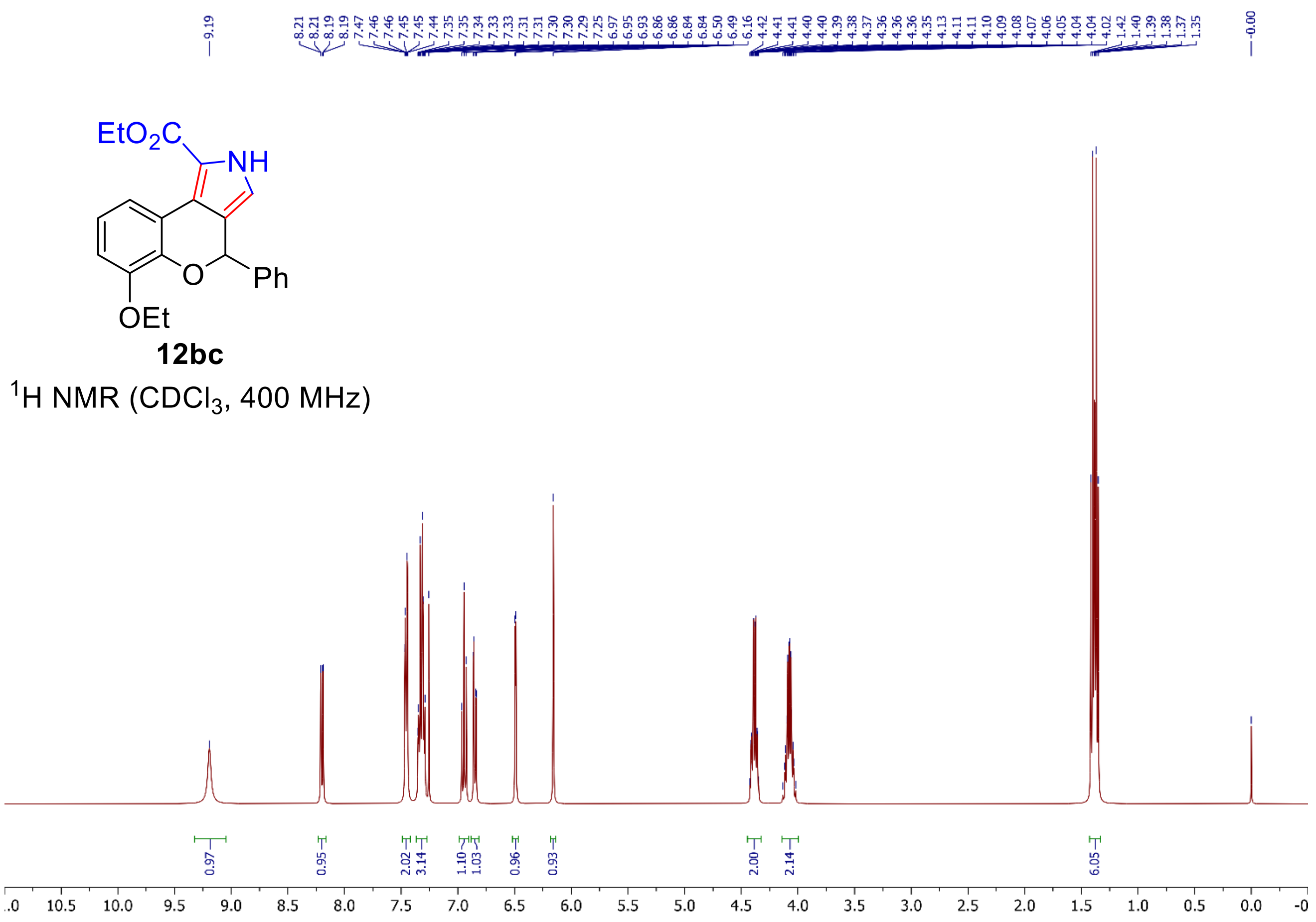

12bc

^{1}H NMR ($CDCl_3$, 400 MHz)

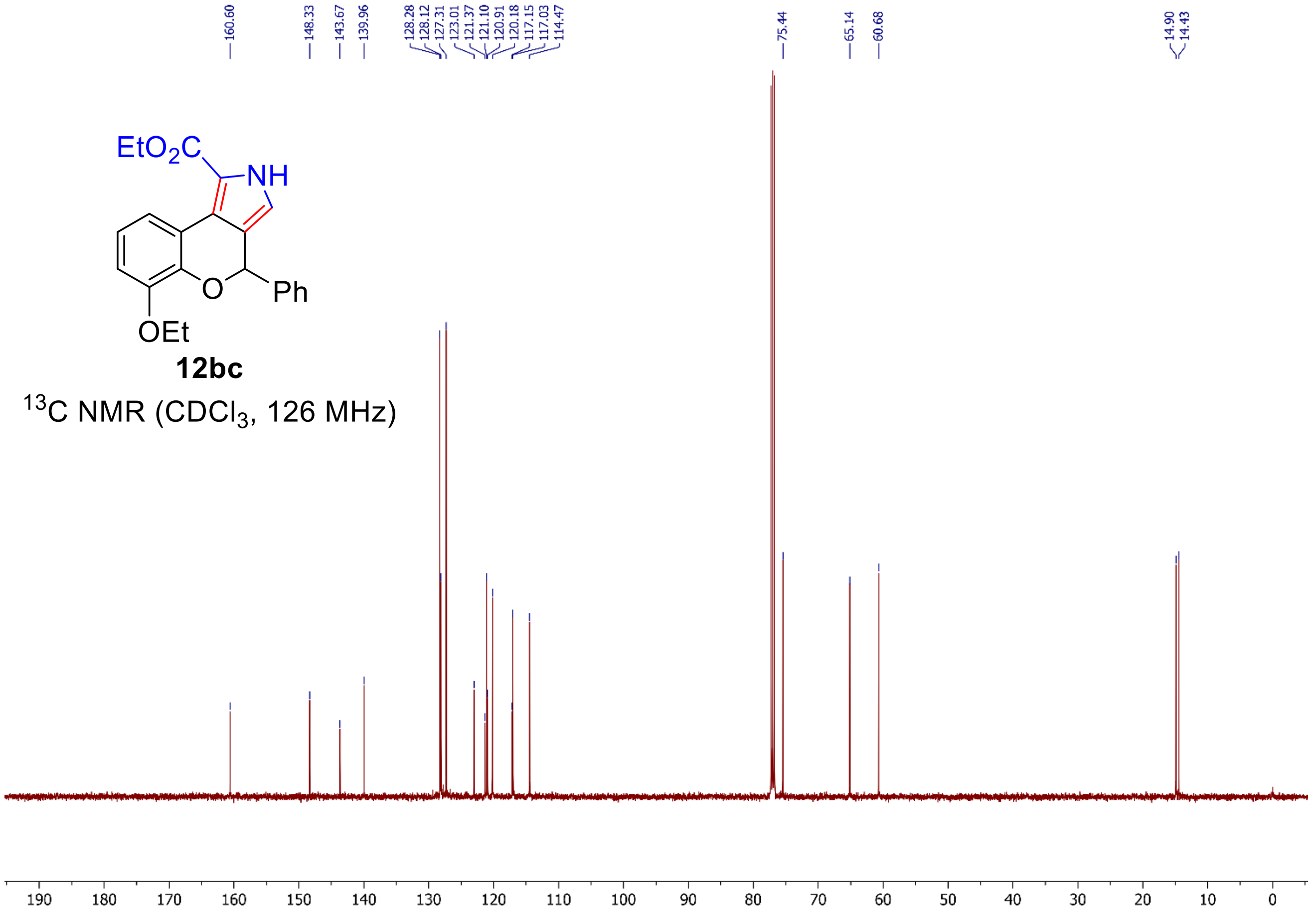

12bc

^{13}C NMR ($CDCl_3$, 126 MHz)

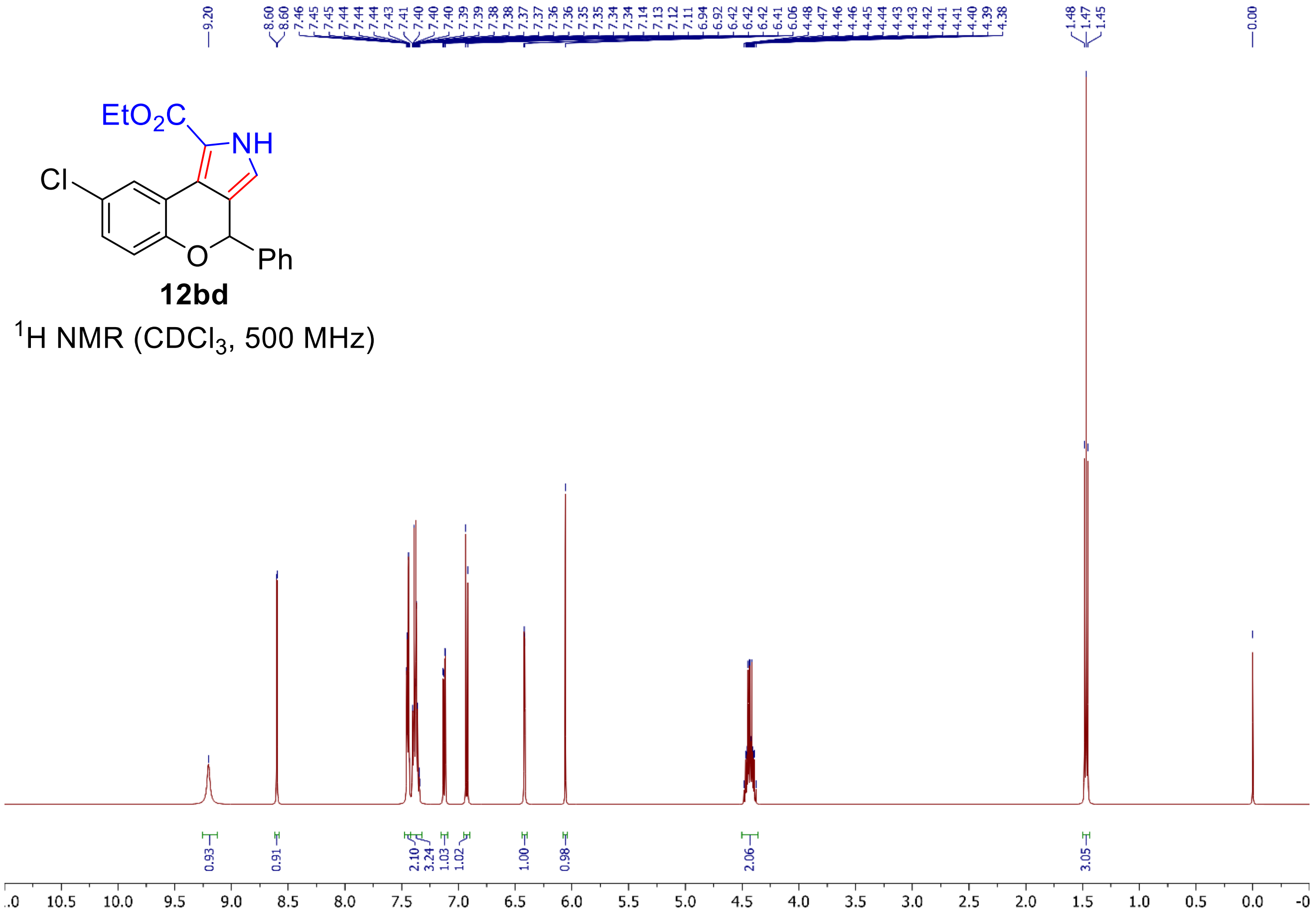
9.20
8.60 8.60 7.46 7.45 7.45 7.44 7.44 7.44 7.43 7.41 7.40 7.40 7.40 7.39 7.39 7.38 7.38 7.37 7.36 7.36 7.35 7.35 7.34 7.34 7.14 7.13 7.12 7.11 6.94 6.92 6.42 6.42 6.41 6.06 4.48 4.47 4.46 4.46 4.45 4.44 4.43 4.43 4.42 4.41 4.40 4.39 4.38
1.48 1.47 1.45
0.00
EtO2C
NH
Cl
O
Ph
12bd
1H NMR (CDCl3, 500 MHz)
0.93 0.91 2.10 3.24 1.03 1.02 1.00 0.98 2.06 3.05
10.5 10.0 9.5 9.0 8.5 8.0 7.5 7.0 6.5 6.0 5.5 5.0 4.5 4.0 3.5 3.0 2.5 2.0 1.5 1.0 0.5 0.0

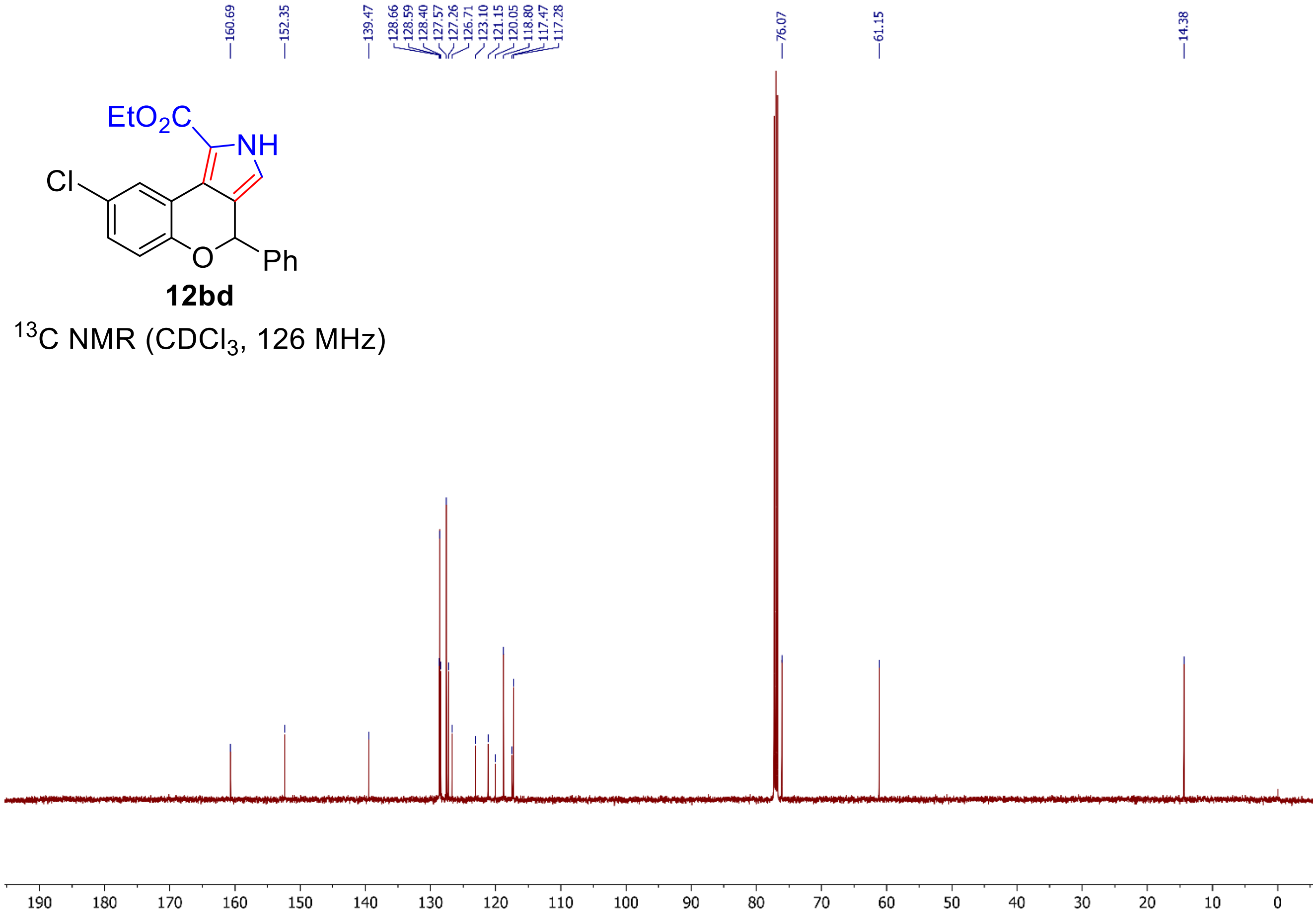
160.69
152.35
139.47
128.66 128.59 128.40 127.57 127.26 126.71 123.10 121.15 120.05 118.80 117.47 117.28
76.07
61.15
14.38
EtO2C
NH
Cl
O
Ph
12bd
13C NMR (CDCl3, 126 MHz)
190 180 170 160 150 140 130 120 110 100 90 80 70 60 50 40 30 20 10 0

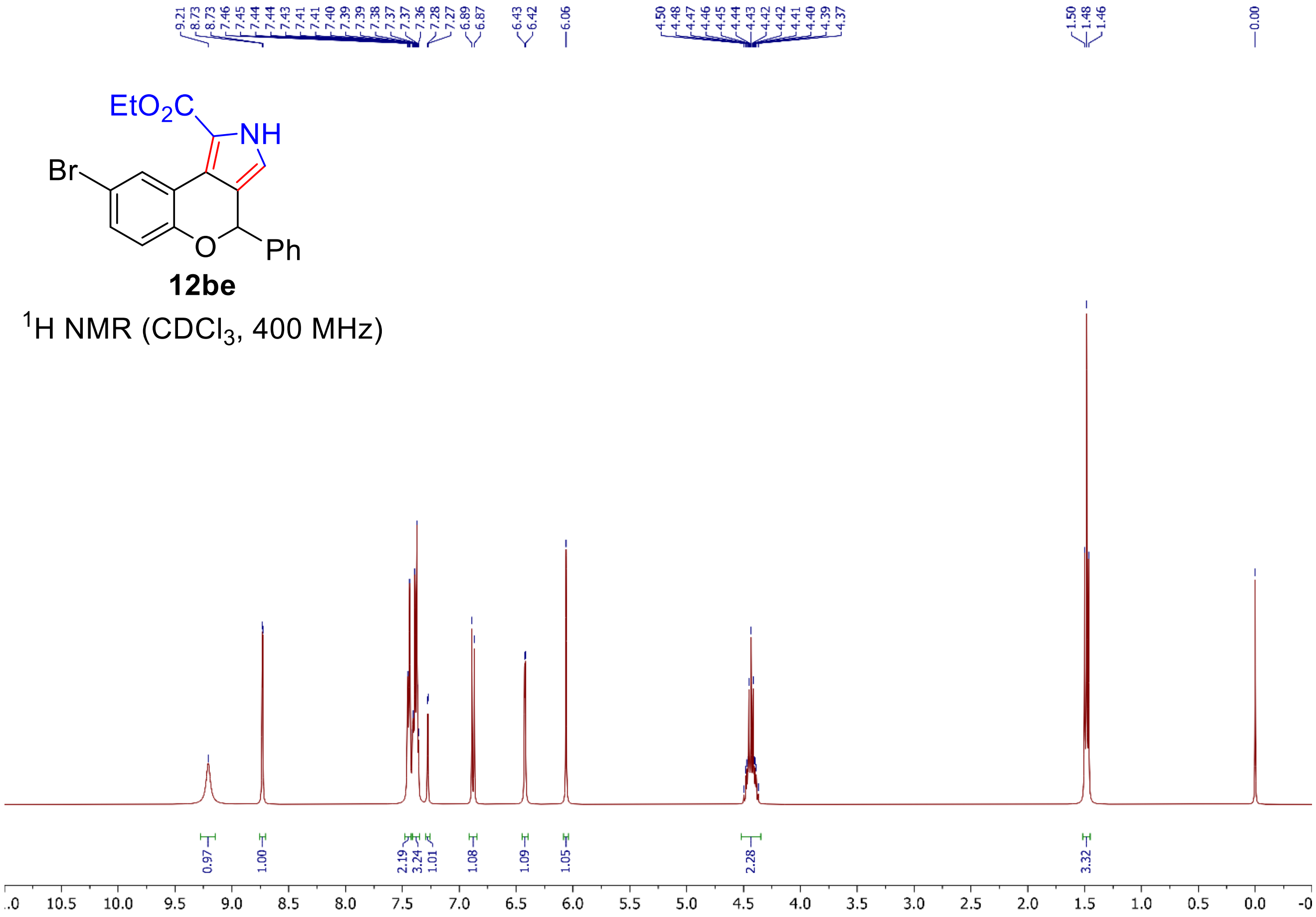

12be

^{1}H NMR ($CDCl_3$, 400 MHz)

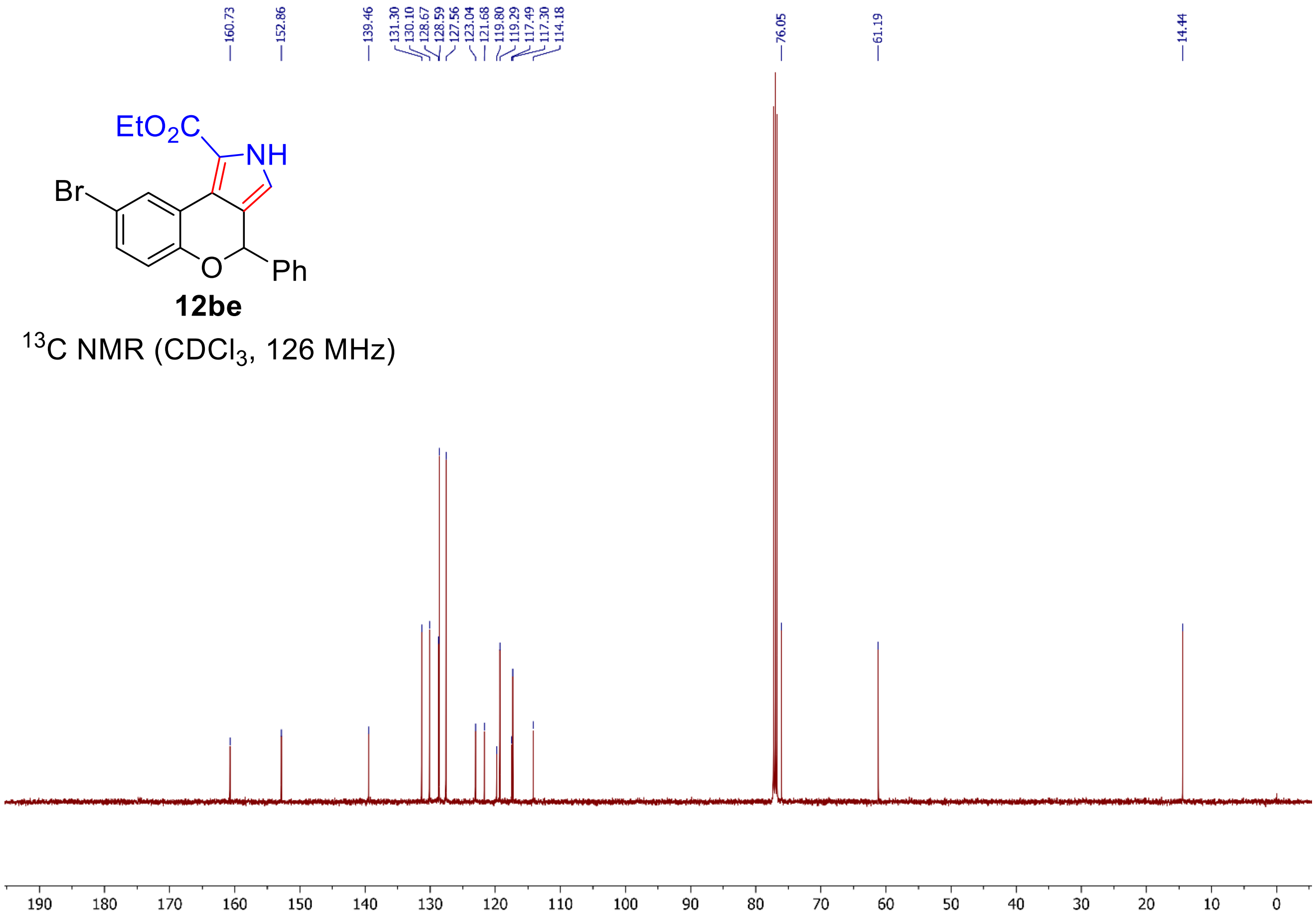

12be

^{13}C NMR ($CDCl_3$, 126 MHz)

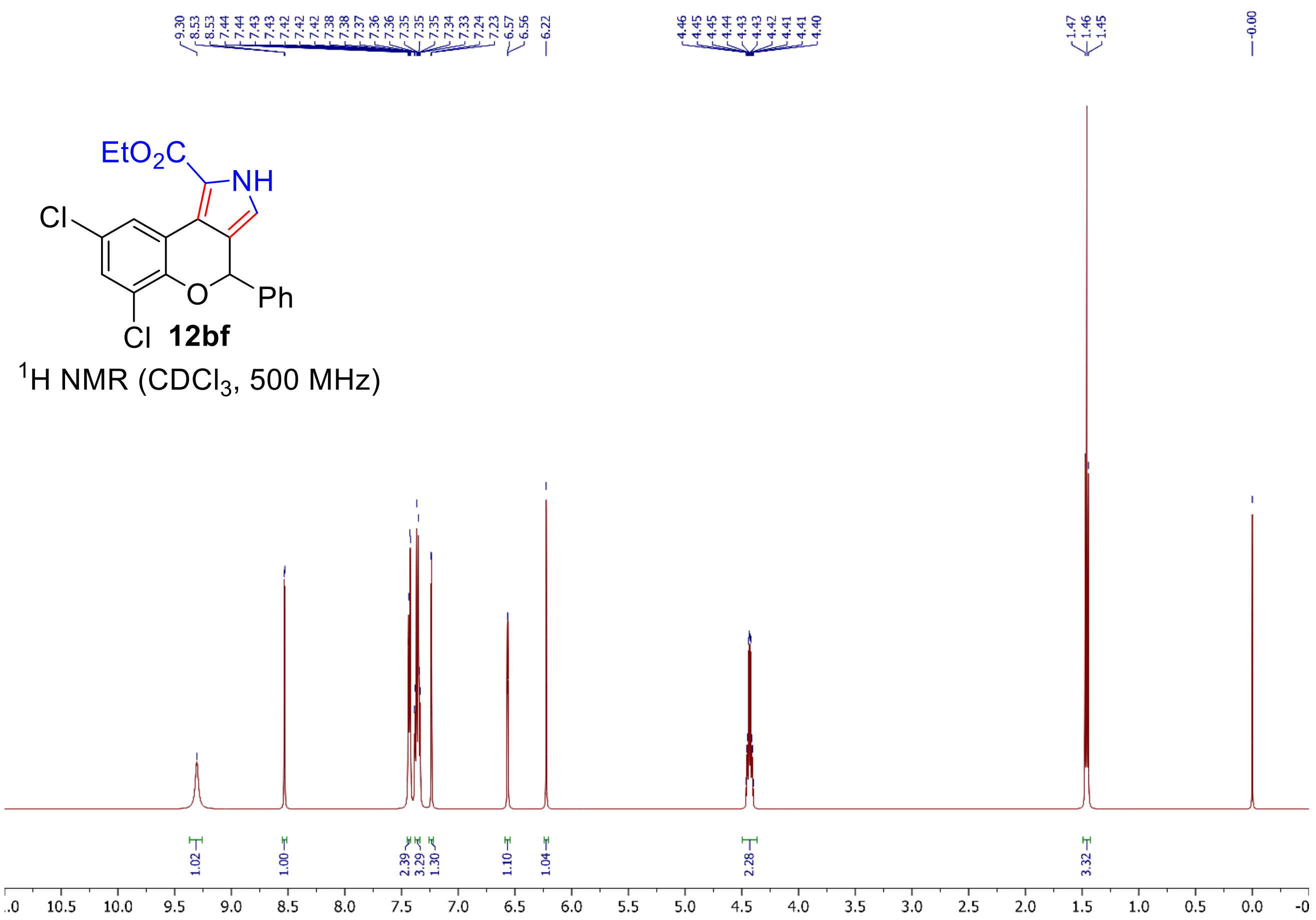

12bf

^{1}H NMR ($CDCl_3$, 500 MHz)

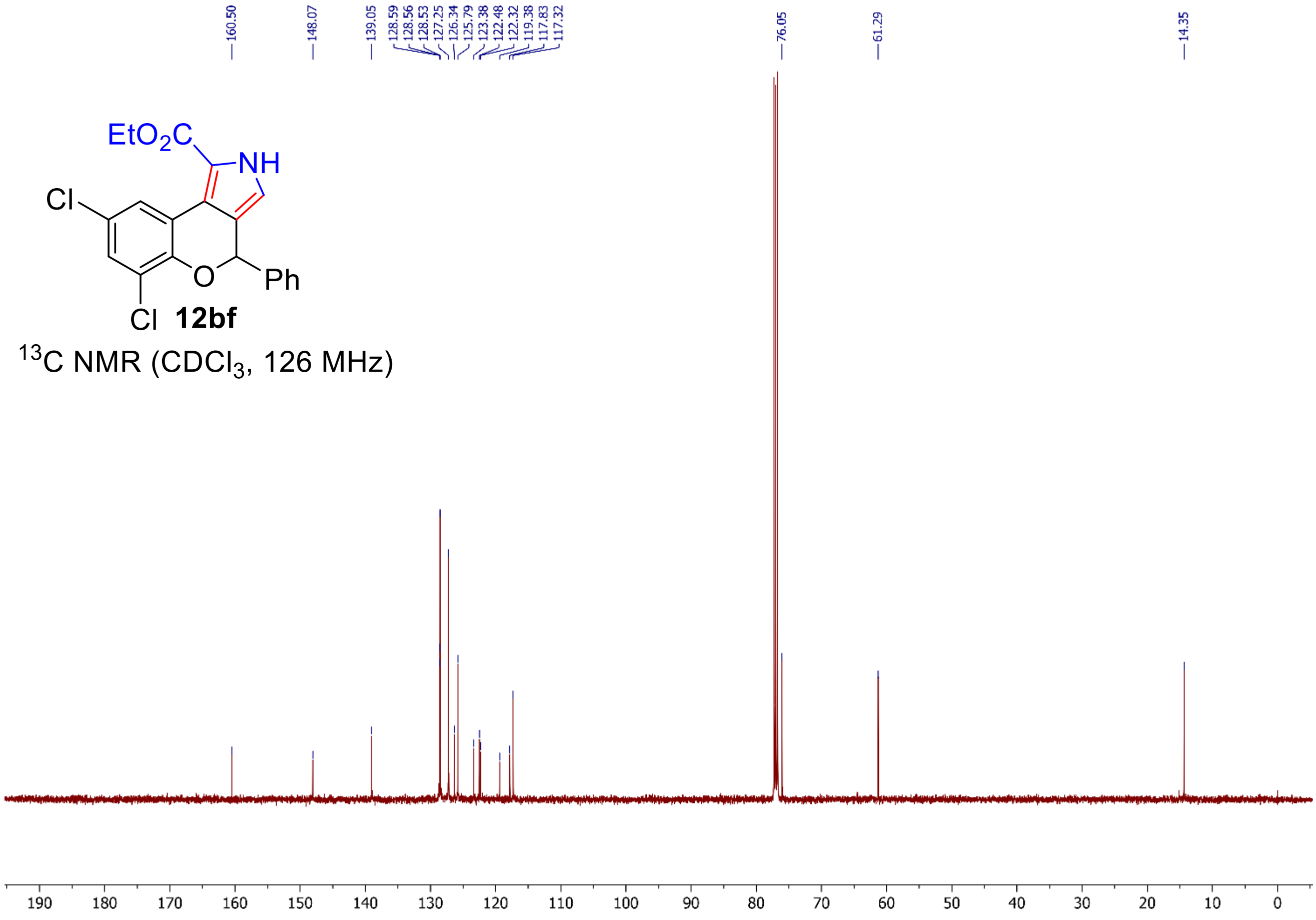

12bf

^{13}C NMR ($CDCl_3$, 126 MHz)

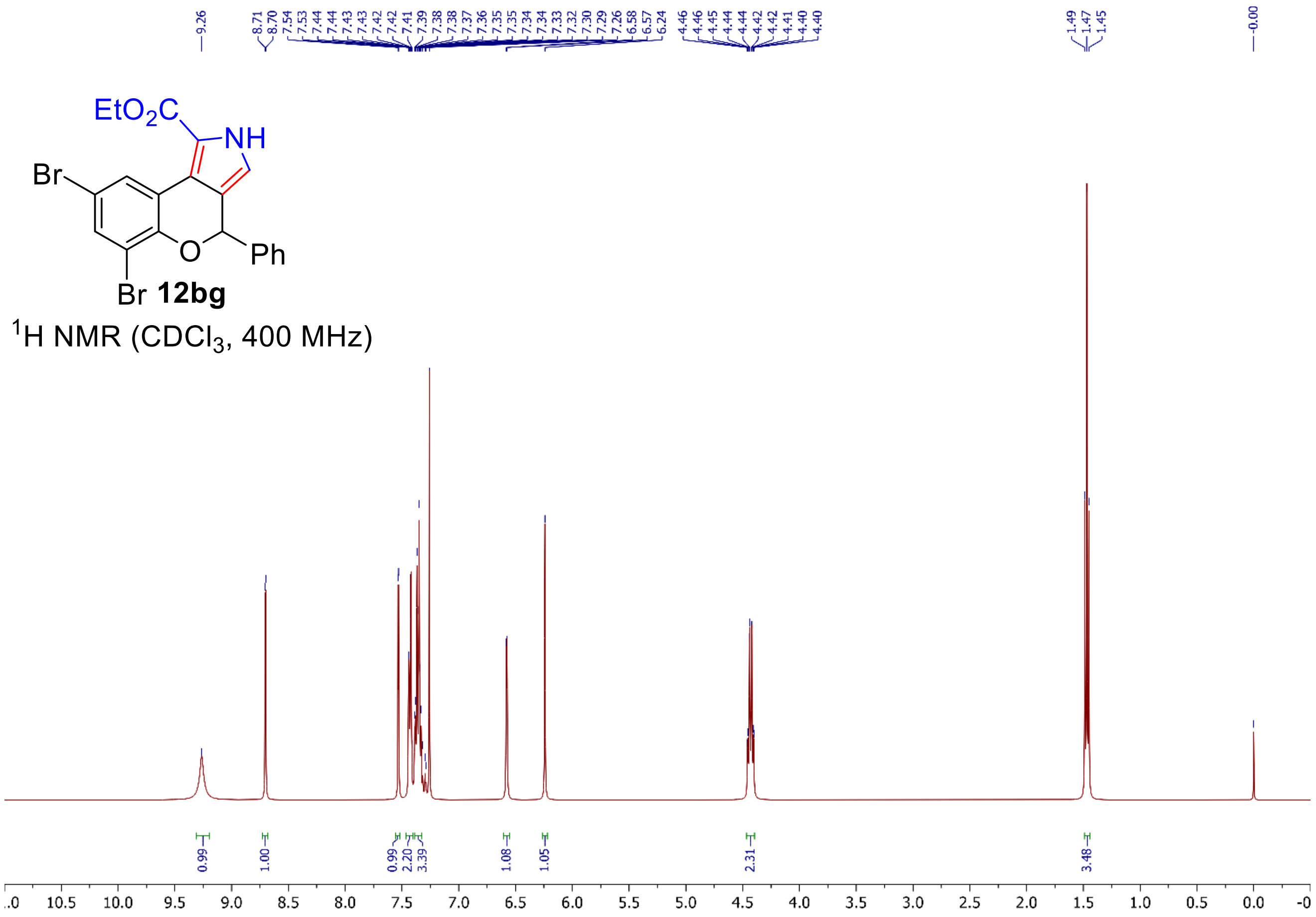

EtO2C
NH
Br
O
Ph
Br 12bg
1H NMR (CDCl3, 400 MHz)

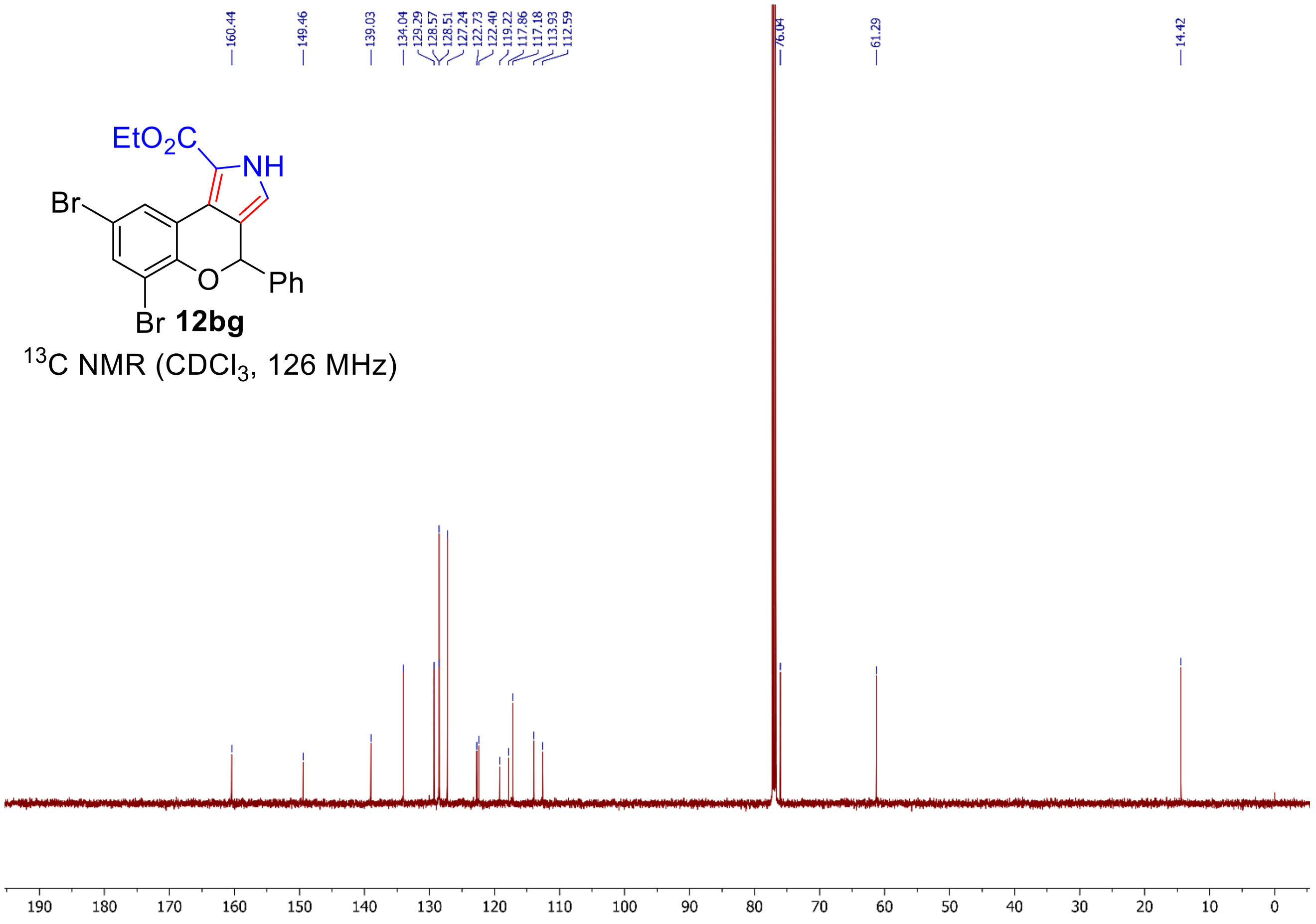

EtO2C
NH
Br
O
Ph
Br 12bg
13C NMR (CDCl3, 126 MHz)

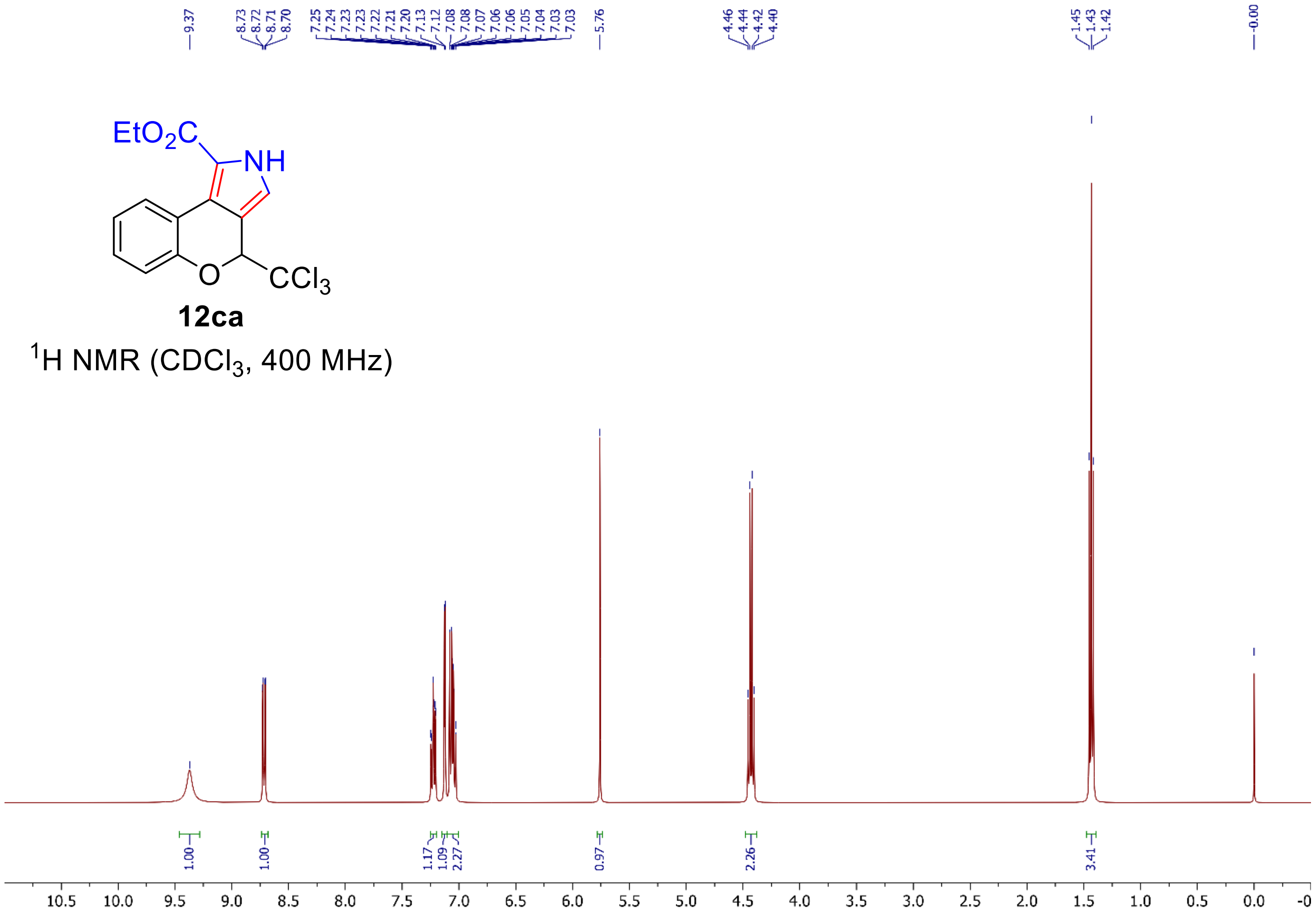
9.37
8.73 8.72 8.71 8.70
7.25 7.24 7.23 7.23 7.22 7.21 7.20 7.13 7.12 7.08 7.08 7.07 7.06 7.06 7.05 7.04 7.03 7.03
5.76
4.46 4.44 4.42 4.40
1.45 1.43 1.42
-0.00
EtO2C
NH
O
CCl3
12ca
1H NMR (CDCl3, 400 MHz)
1.00 1.00 1.17 1.09 2.27 0.97 2.26 3.41

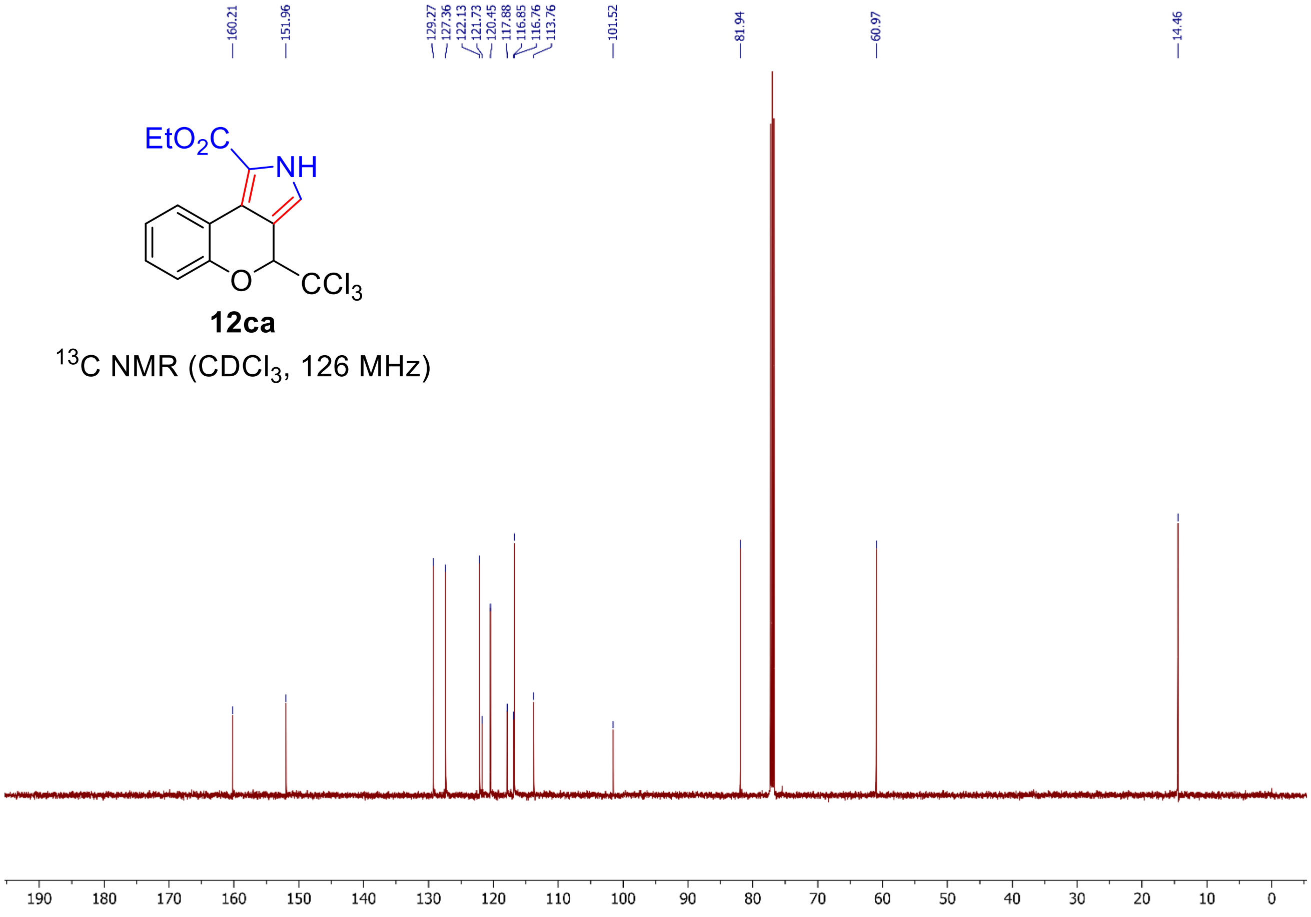
160.21
151.96
129.27 127.36 122.13 121.73 120.45 117.88 116.85 116.76 113.76
101.52
81.94
60.97
14.46
EtO2C
NH
O
CCl3
12ca
13C NMR (CDCl3, 126 MHz)

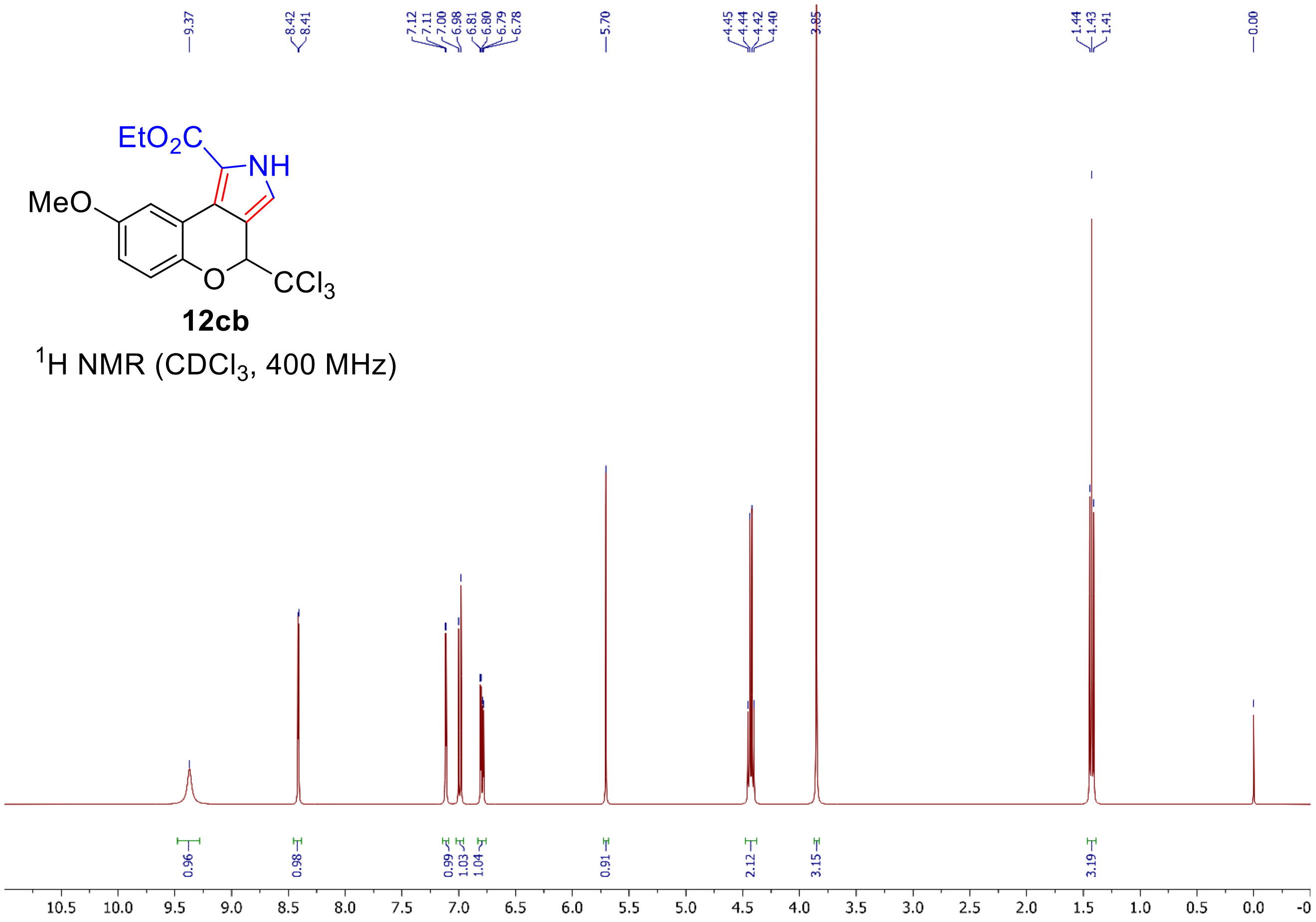

12cb

^{1}H NMR ($CDCl_3$, 400 MHz)

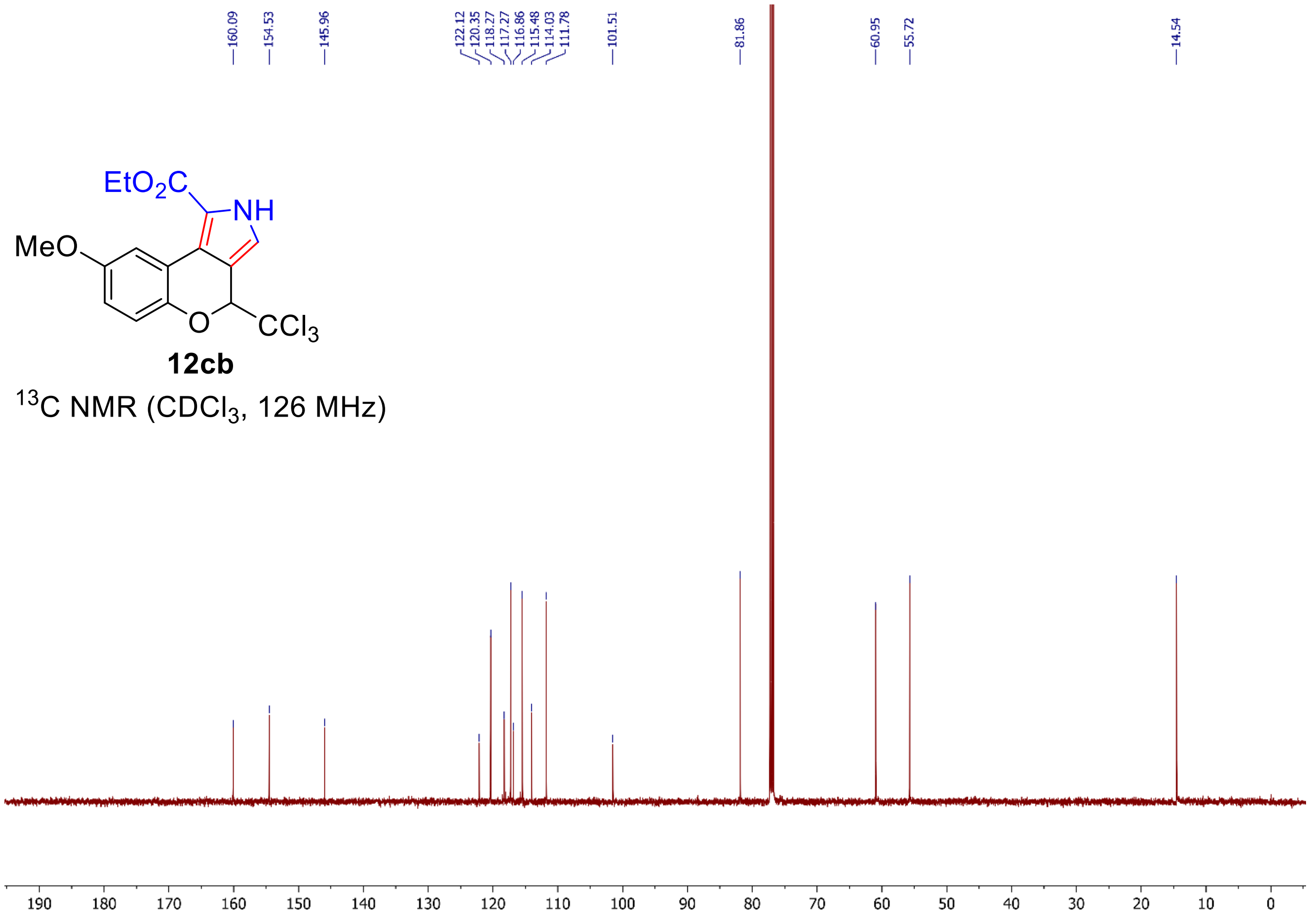

12cb

^{13}C NMR ($CDCl_3$, 126 MHz)

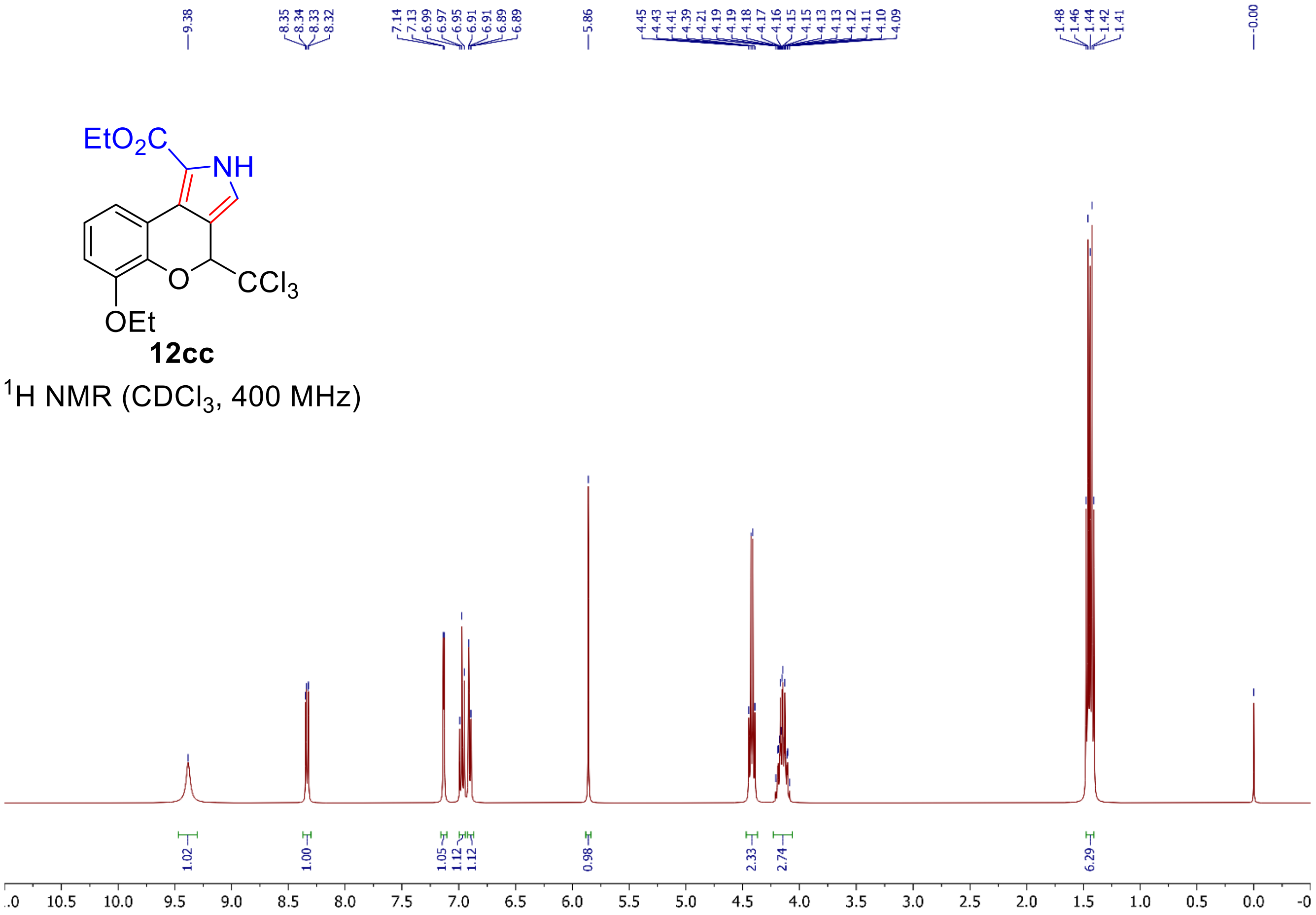
9.38
8.35 8.34 8.33 8.32
7.14 7.13 6.99 6.97 6.95 6.91 6.91 6.89 6.89
5.86
4.45 4.43 4.41 4.39 4.21 4.19 4.19 4.18 4.17 4.16 4.15 4.15 4.13 4.13 4.12 4.11 4.10 4.09
1.48 1.46 1.44 1.42 1.41
0.00
EtO2C
NH
O
CCl3
OEt
12cc
1H NMR (CDCl3, 400 MHz)
1.02
1.00
1.05
1.12
1.12
0.98
2.33
2.74
6.29

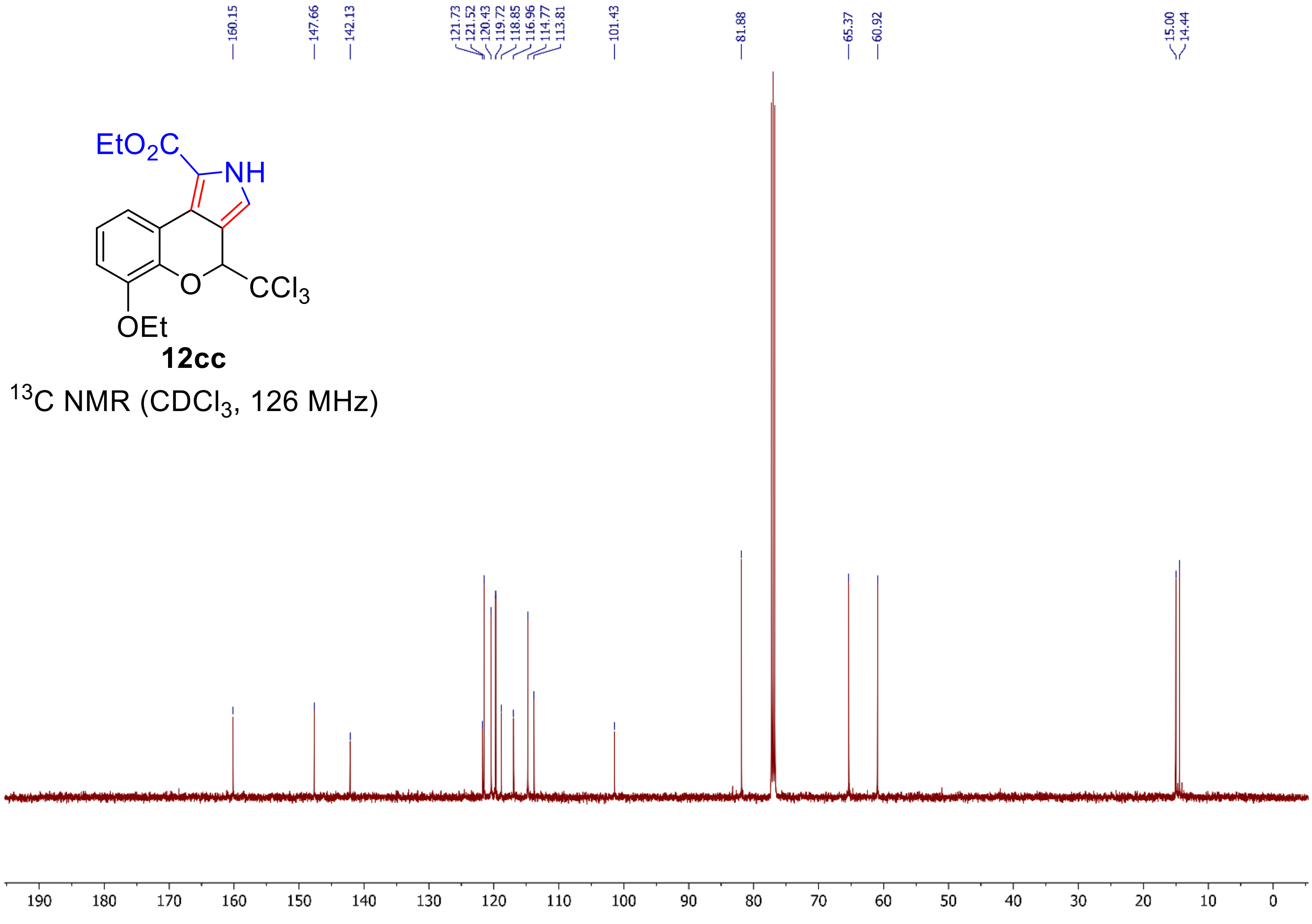
160.15
147.66
142.13
121.73 121.52 120.43 119.72 118.85 116.96 114.77 113.81
101.43
81.88
65.37
60.92
15.00 14.44
EtO2C
NH
O
CCl3
OEt
12cc
13C NMR (CDCl3, 126 MHz)

12cd

^{1}H NMR ($CDCl_3$, 400 MHz)

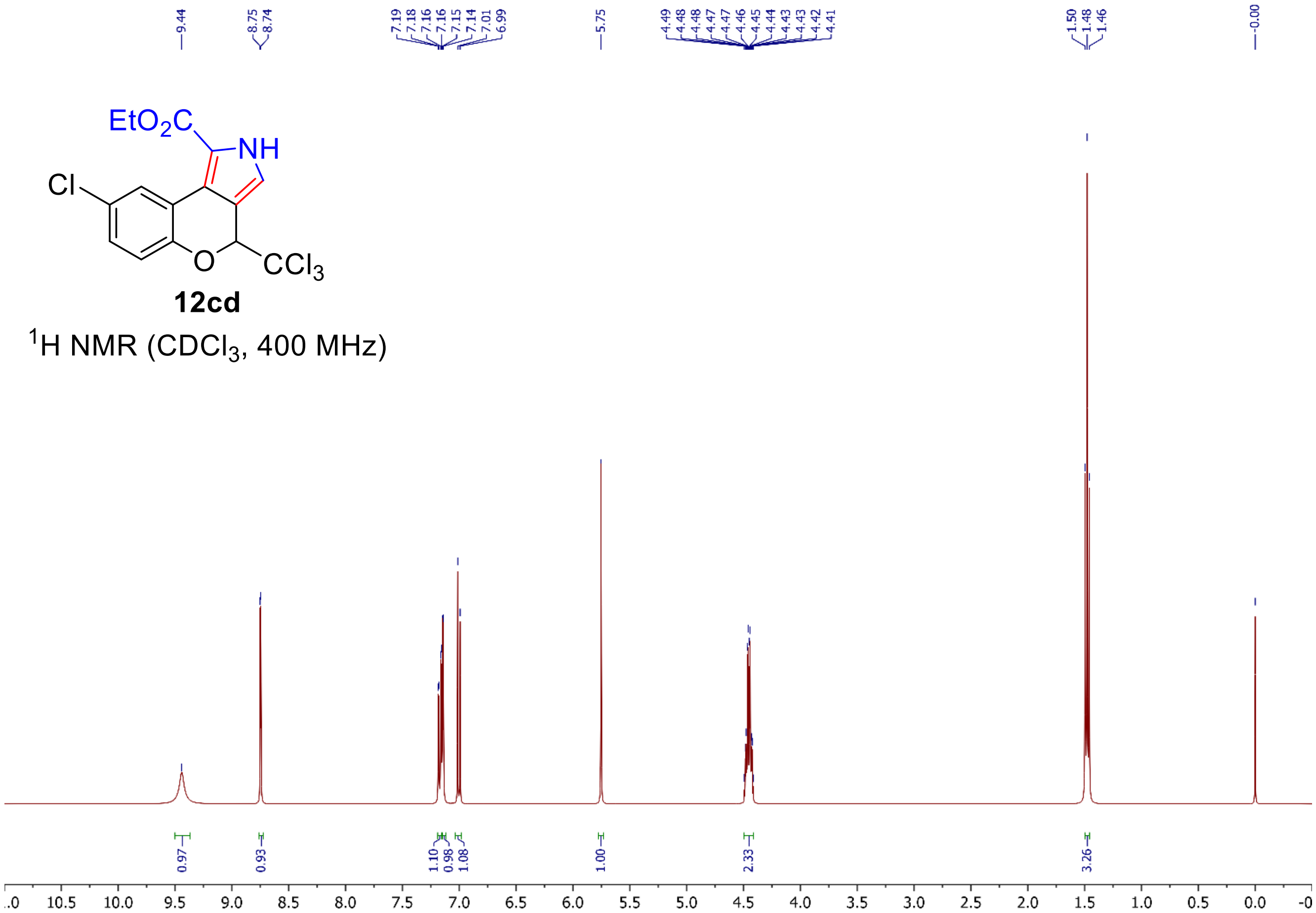

12cd

^{13}C NMR ($CDCl_3$, 126 MHz)

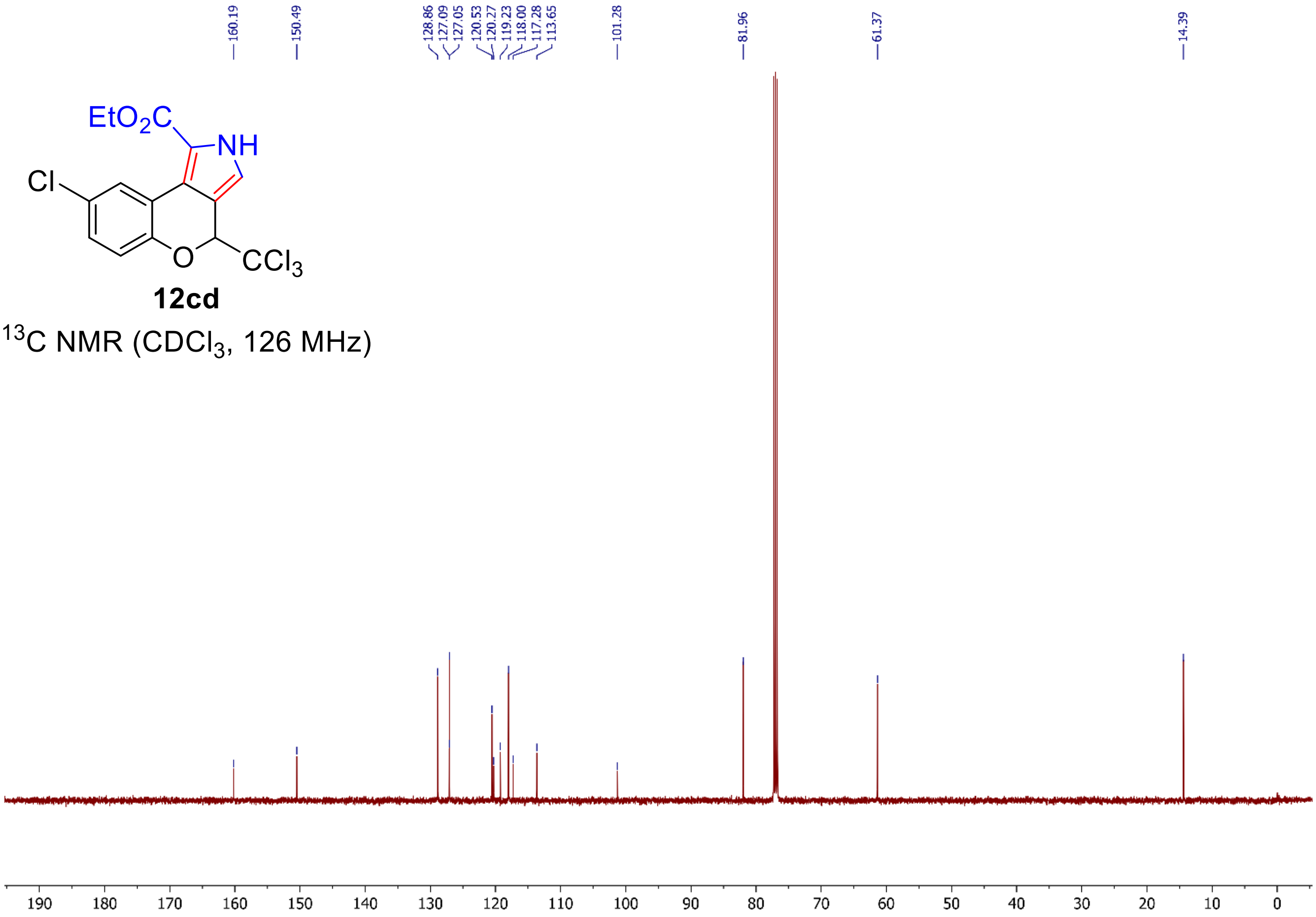

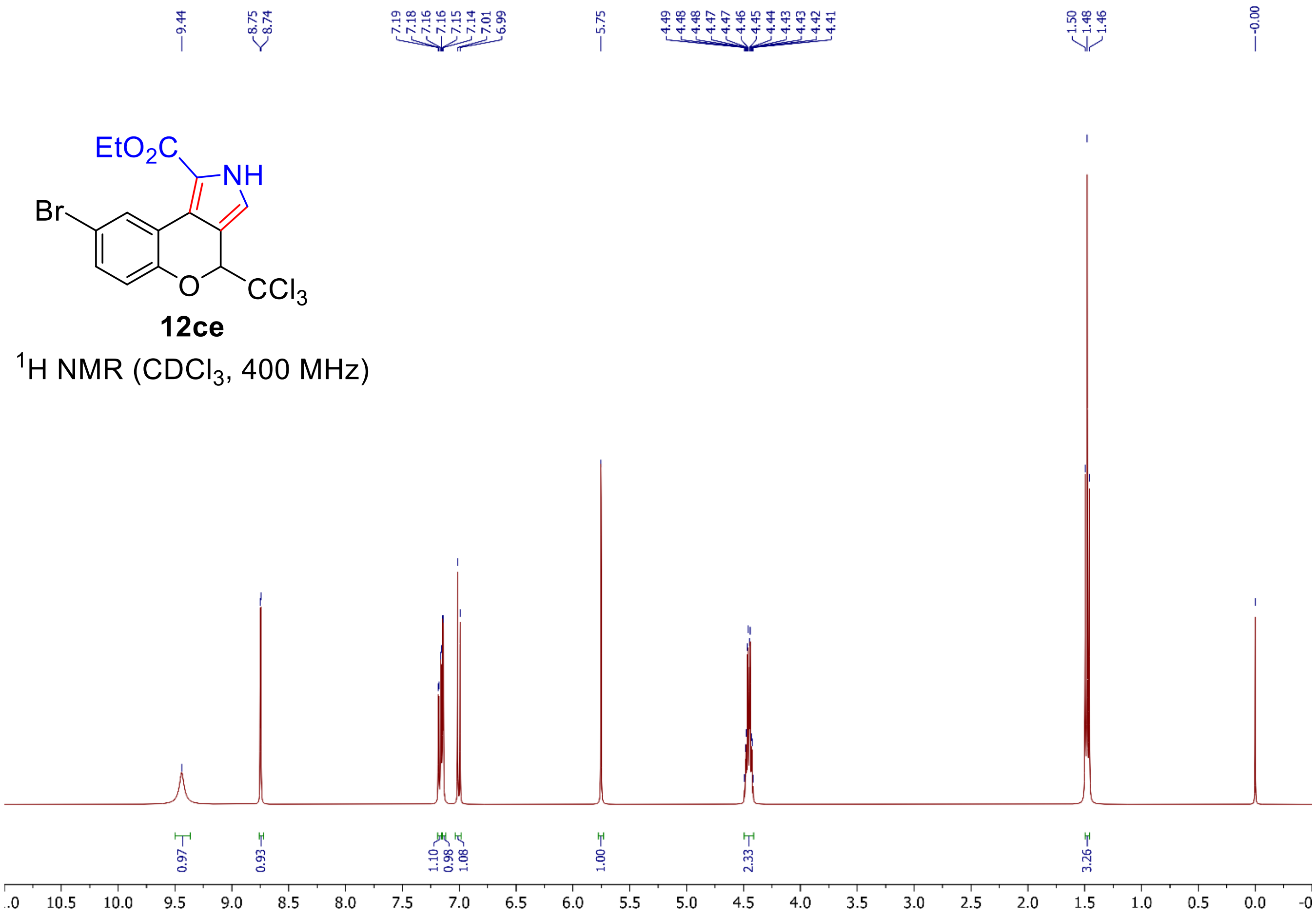
EtO2C
NH
Br
O
CCl3
12ce
1H NMR (CDCl3, 400 MHz)

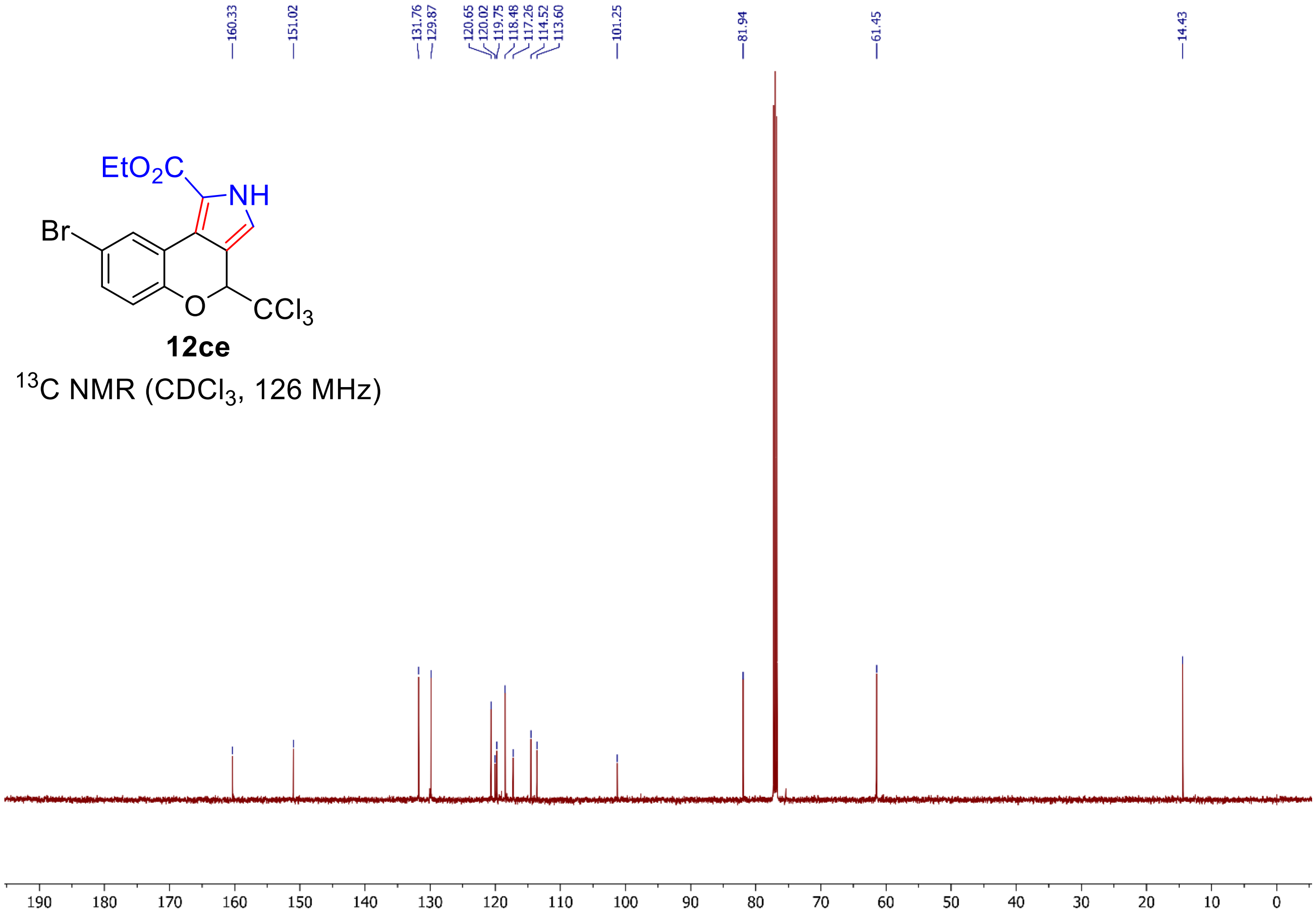
EtO2C
NH
Br
O
CCl3
12ce
13C NMR (CDCl3, 126 MHz)

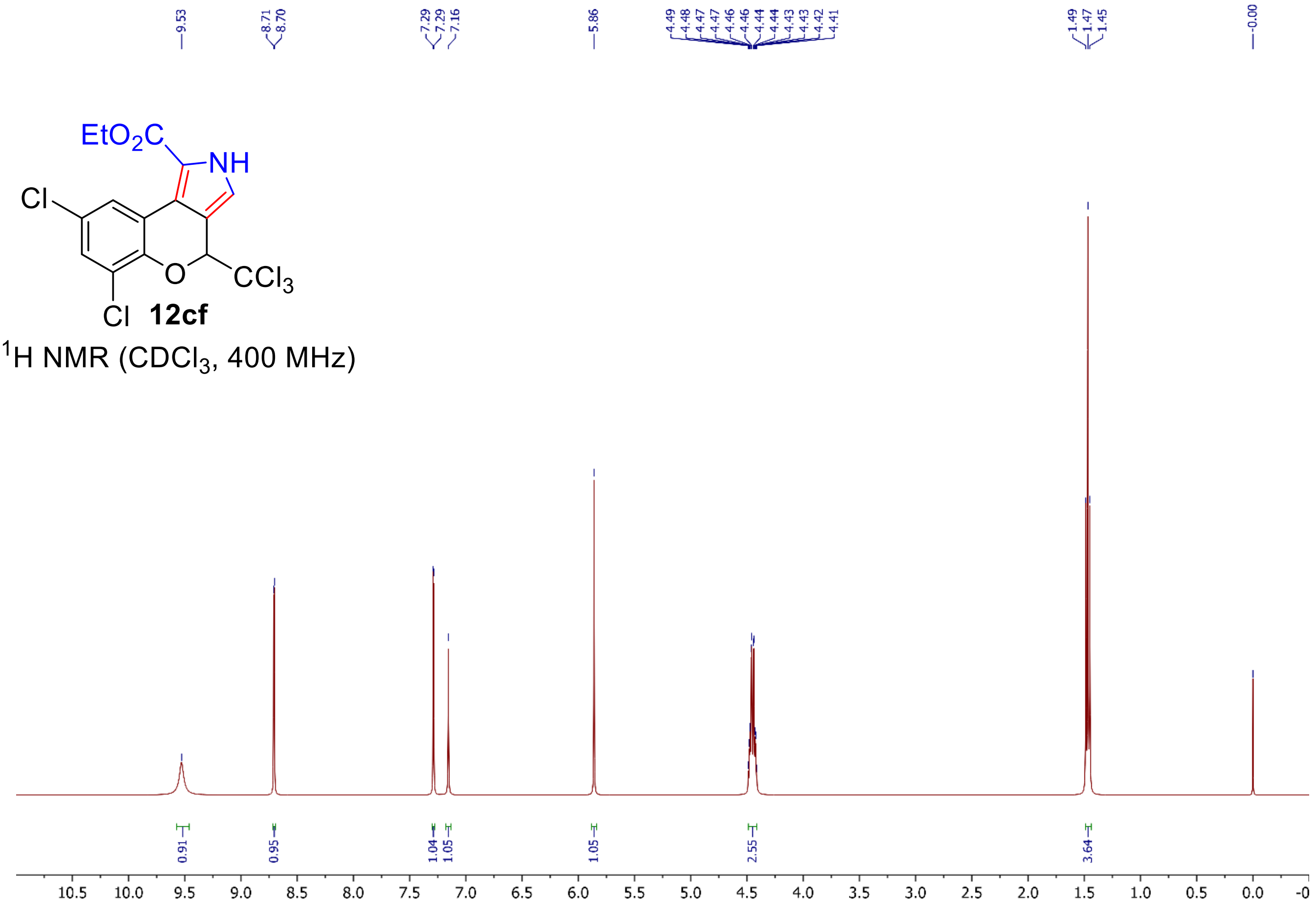

EtO2C
NH
Cl
O
CCl3
Cl 12cf
1H NMR (CDCl3, 400 MHz)
9.53
8.71 8.70
7.29 7.29 7.16
5.86
4.49 4.48 4.47 4.46 4.46 4.44 4.44 4.43 4.43 4.42 4.41
1.49 1.47 1.45
-0.00
0.91 0.95 1.04 1.05 1.05 2.55 3.64

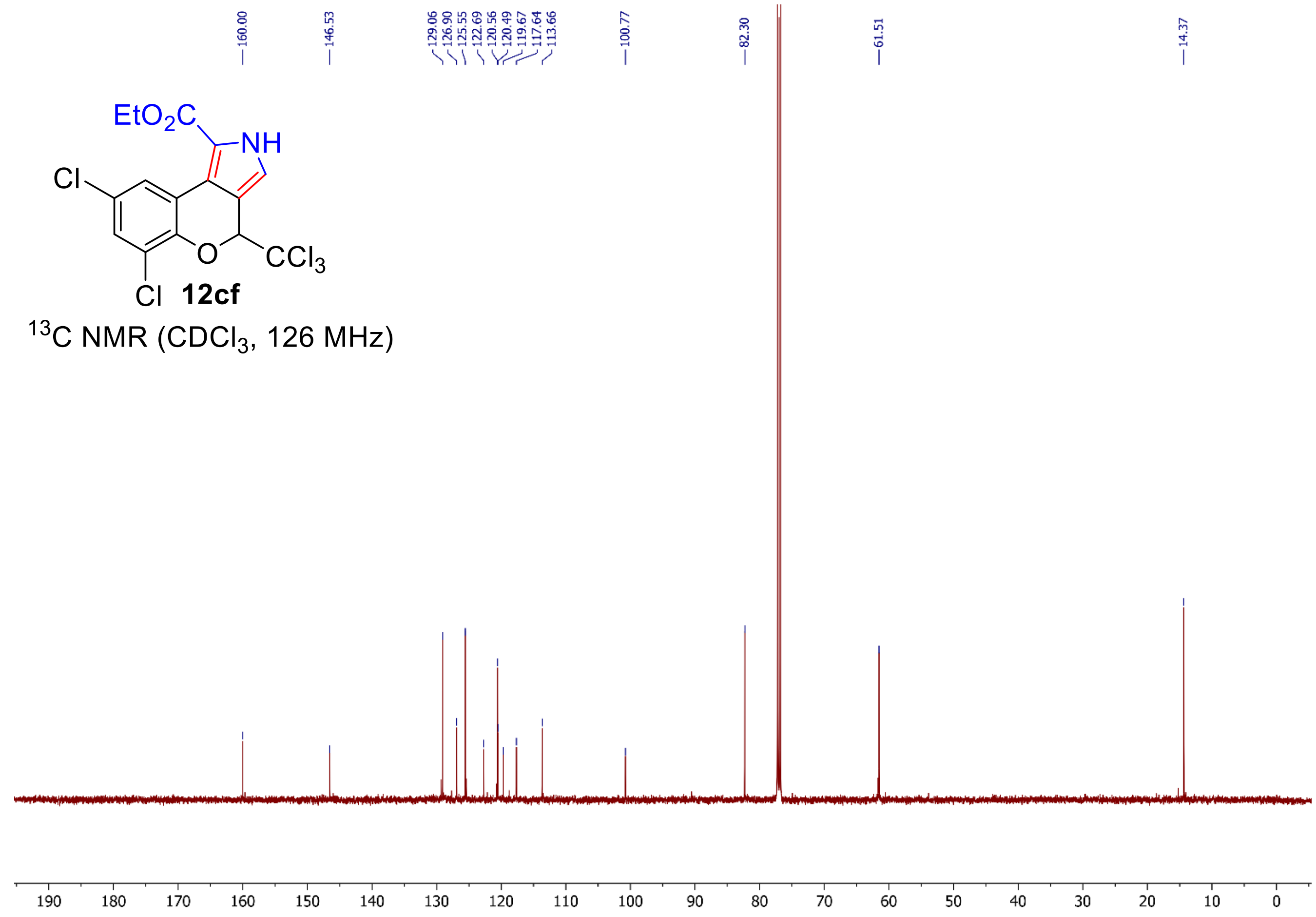

EtO2C
NH
Cl
O
CCl3
Cl 12cf
13C NMR (CDCl3, 126 MHz)
160.00
146.53
129.06 126.90 125.55 122.69 120.56 120.49 119.67 117.64 113.66
100.77
82.30
61.51
14.37

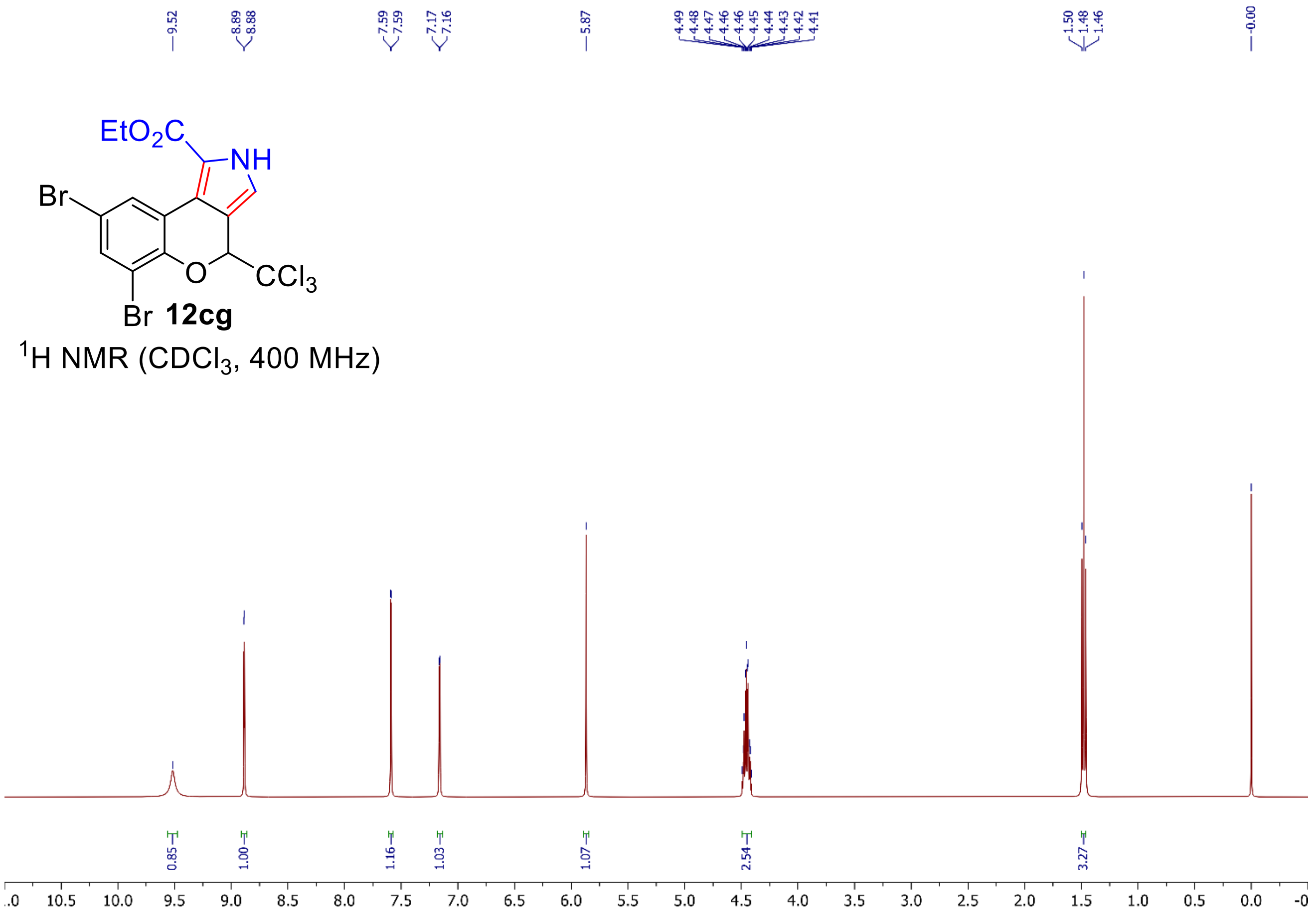
EtO2C
NH
Br
O
CCl3
Br
12cg
1H NMR (CDCl3, 400 MHz)
9.52
8.89
8.88
7.59
7.59
7.17
7.16
5.87
4.49
4.48
4.47
4.46
4.46
4.45
4.44
4.43
4.42
4.41
1.50
1.48
1.46
-0.00
0.85
1.00
1.16
1.03
1.07
2.54
3.27

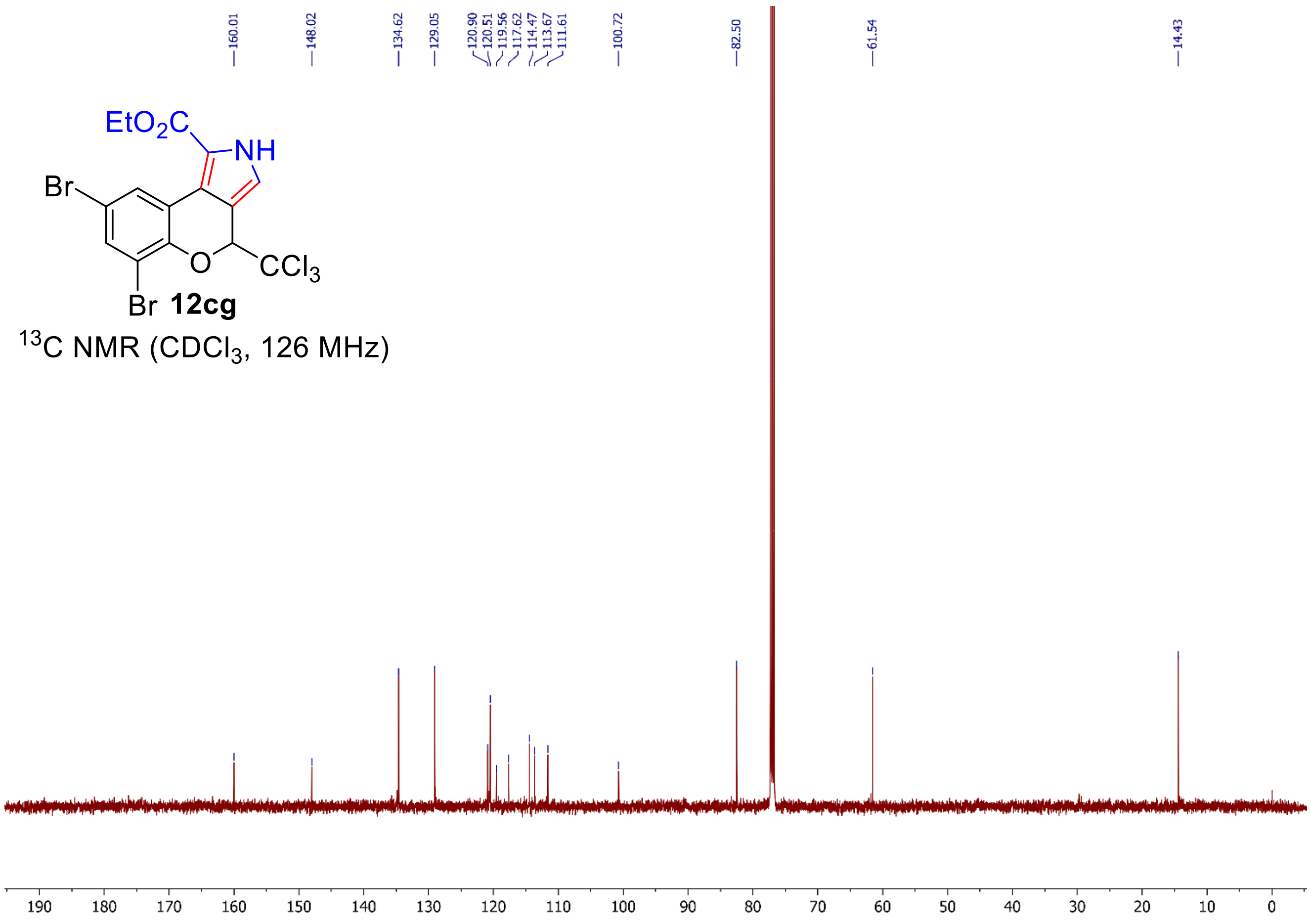
EtO2C
NH
Br
O
CCl3
Br
12cg
13C NMR (CDCl3, 126 MHz)
160.01
148.02
134.62
129.05
120.90
120.51
119.56
117.62
114.47
113.67
111.61
100.72
82.50
61.54
14.43

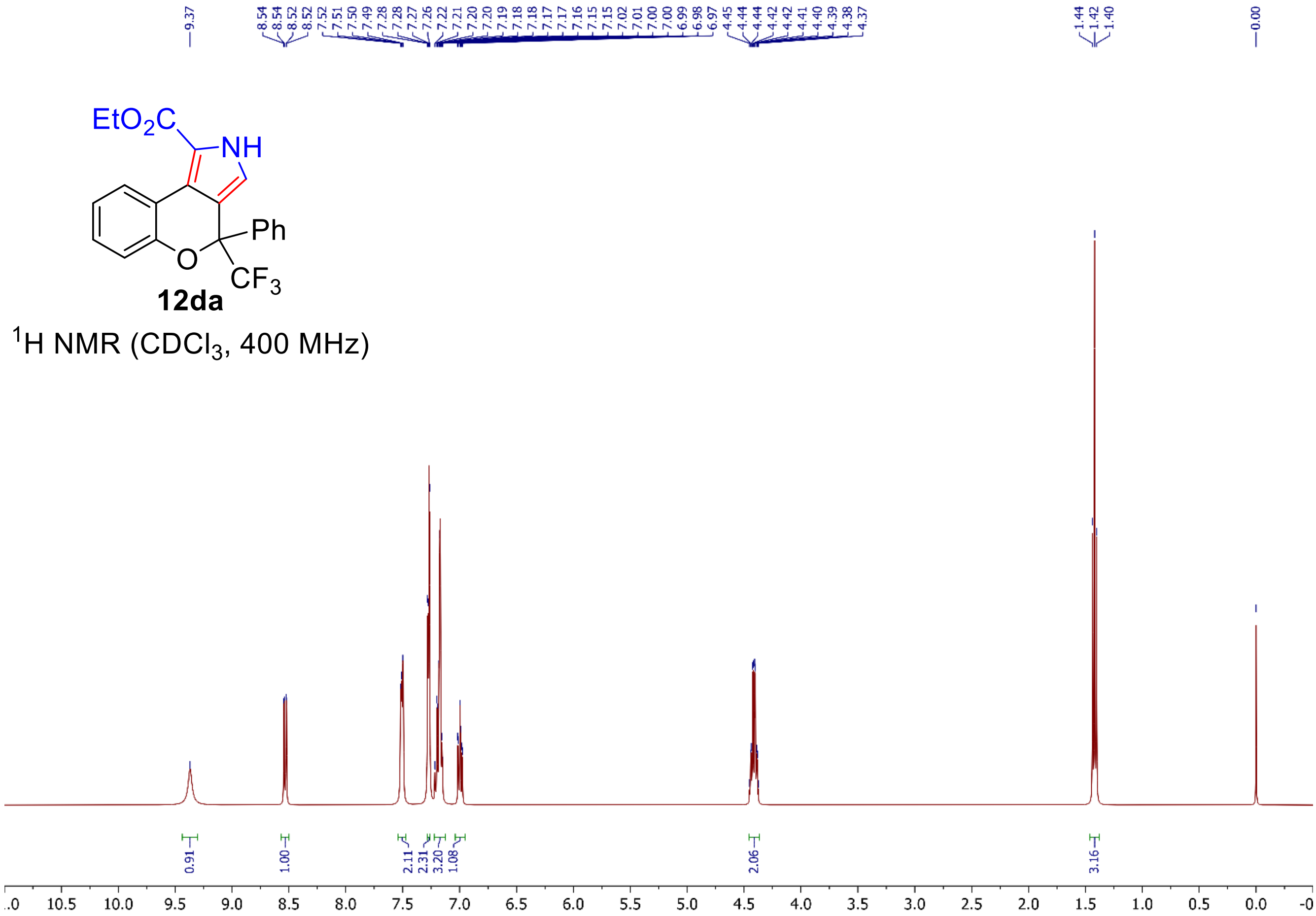
EtO2C
NH
Ph
O
CF3
12da
1H NMR (CDCl3, 400 MHz)

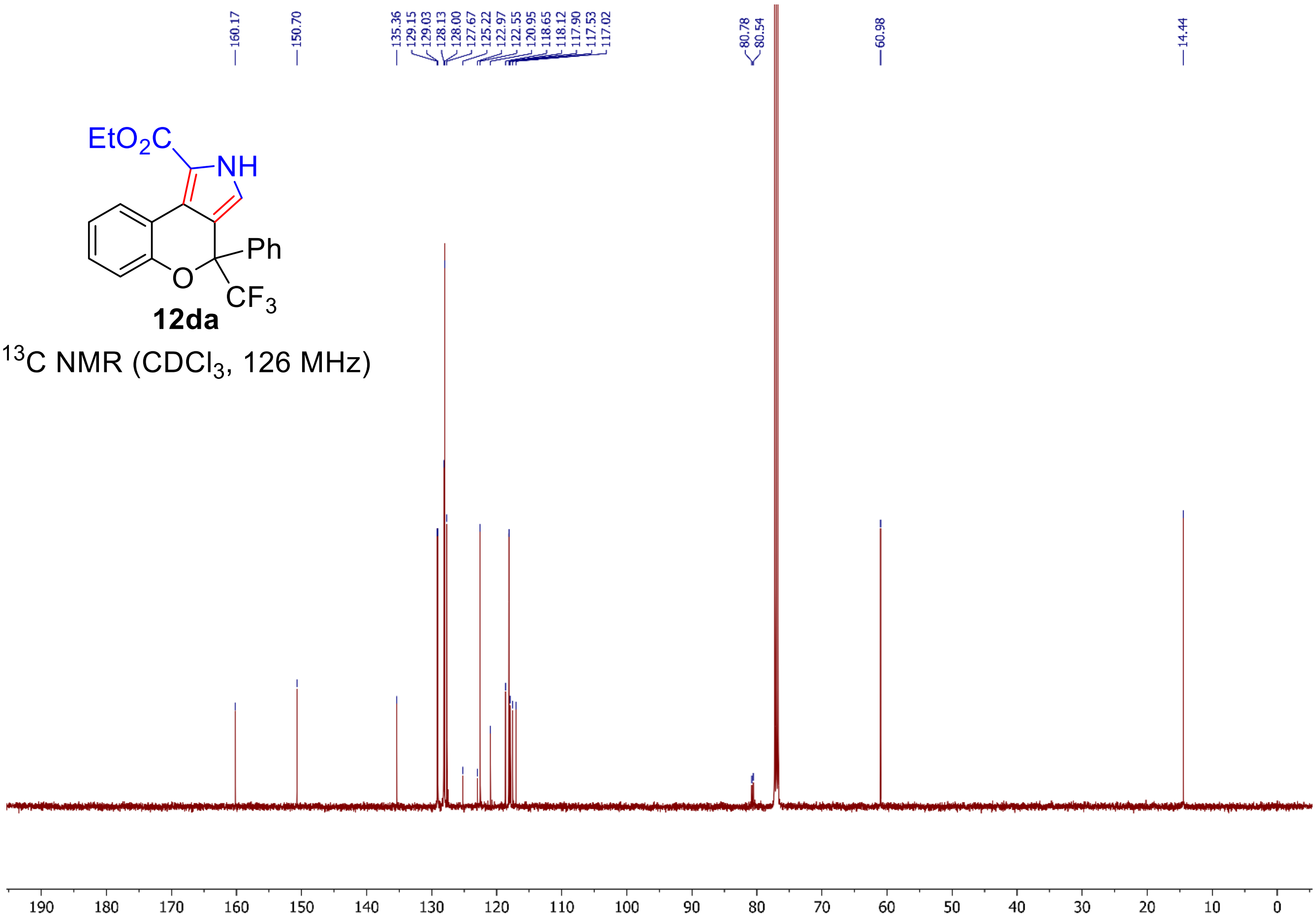
EtO2C
NH
Ph
O
CF3
12da
13C NMR (CDCl3, 126 MHz)

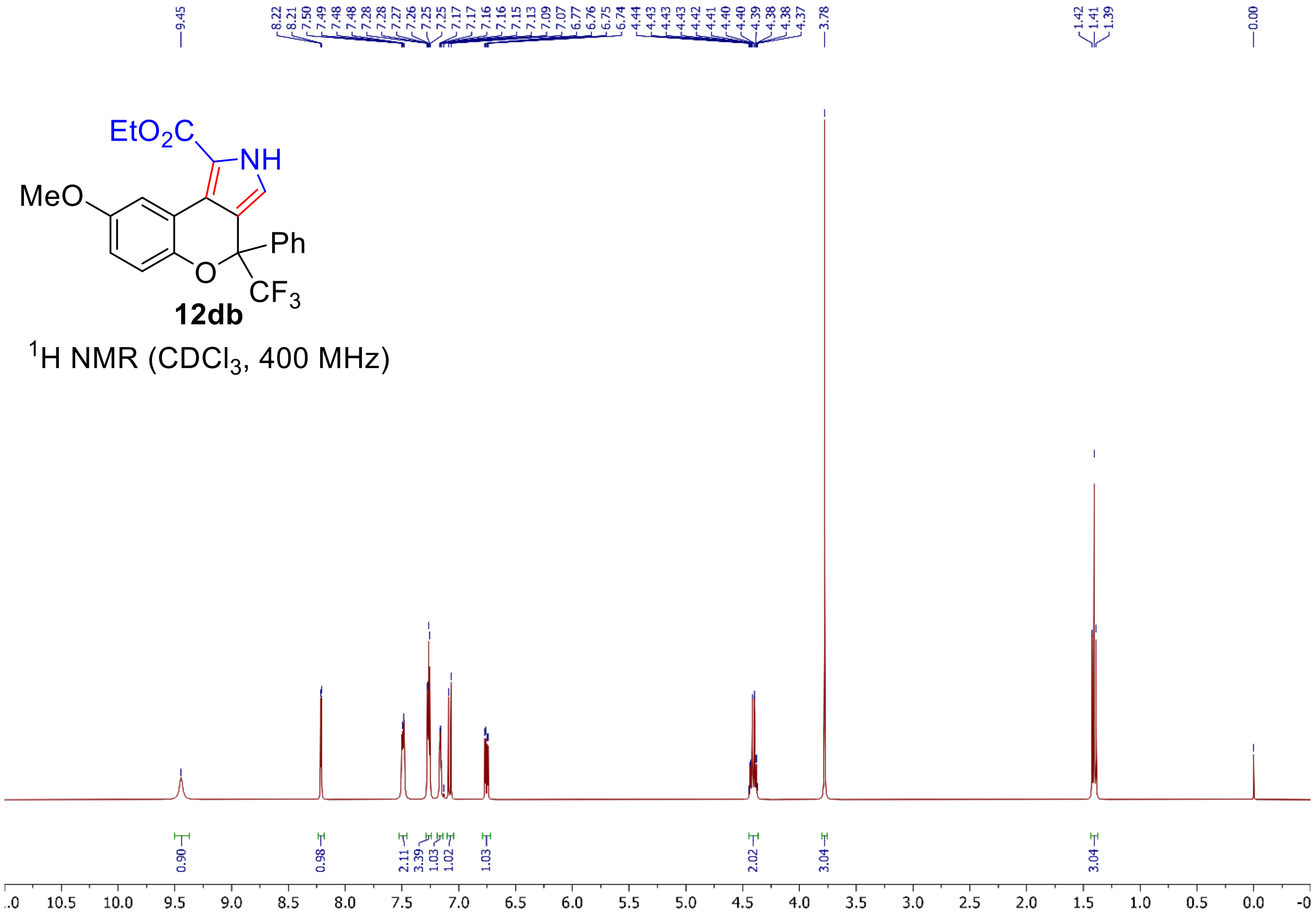
9.45
8.22 8.21 7.50 7.49 7.48 7.48 7.28 7.28 7.27 7.26 7.25 7.25 7.17 7.17 7.16 7.16 7.15 7.13 7.09 7.07 6.77 6.76 6.75 6.74 4.44 4.43 4.43 4.42 4.41 4.40 4.40 4.39 4.38 4.38 4.37 3.78
1.42 1.41 1.39
0.00
EtO2C
NH
MeO
Ph
O
CF3
12db
1H NMR (CDCl3, 400 MHz)
0.90
0.98
2.11
3.39
1.03
1.02
1.03
2.02
3.04
3.04

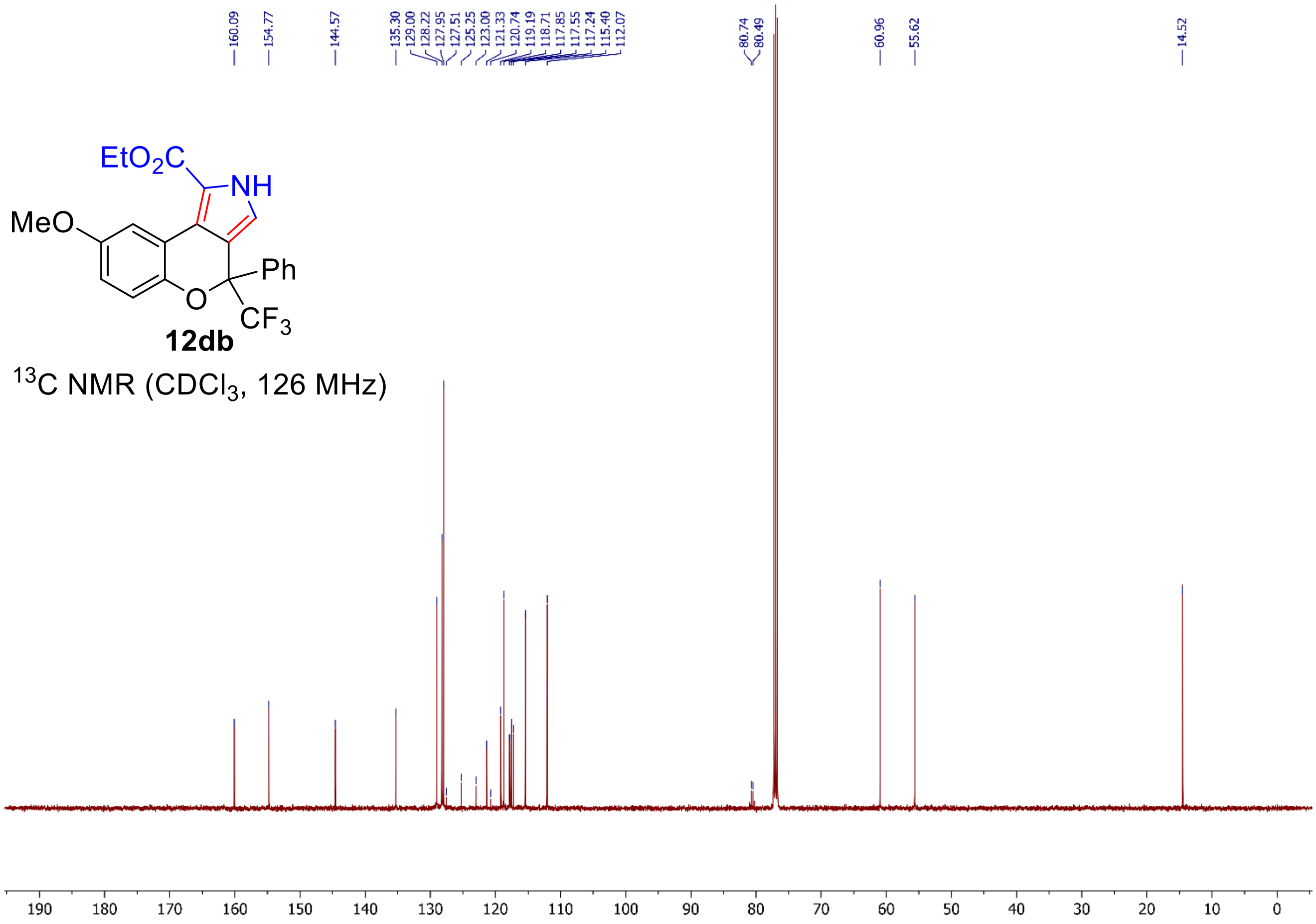
160.09
154.77
144.57
135.30 129.00 128.22 127.95 127.51 125.25 123.00 121.33 120.74 119.19 118.71 117.85 117.55 117.24 115.40 112.07
80.74 80.49
60.96
55.62
14.52
EtO2C
NH
MeO
Ph
O
CF3
12db
13C NMR (CDCl3, 126 MHz)

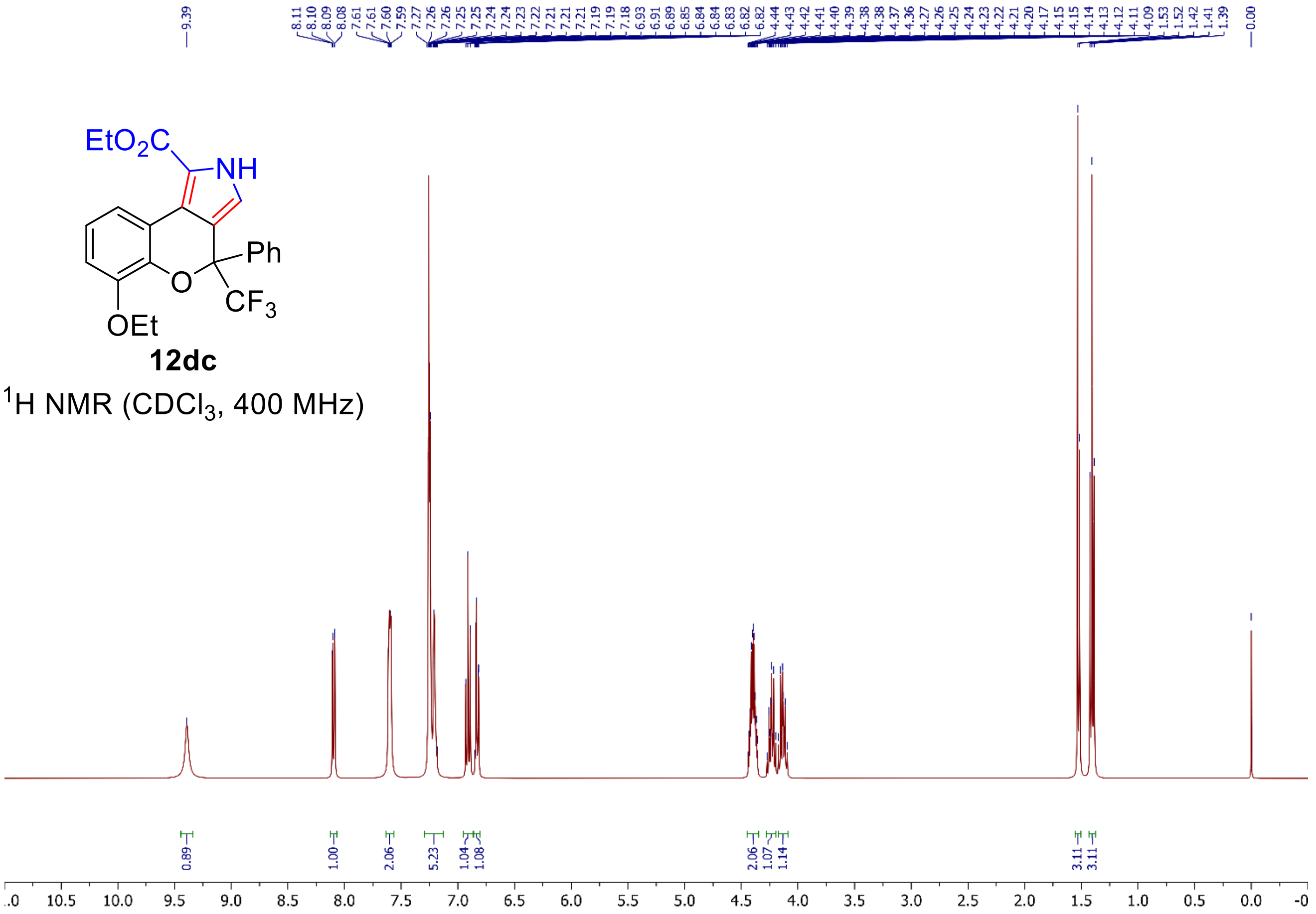
EtO2C
NH
Ph
O
CF3
OEt
12dc
1H NMR (CDCl3, 400 MHz)
9.39
8.11 8.10 8.09 8.08 7.61 7.61 7.60 7.59 7.27 7.26 7.26 7.25 7.25 7.24 7.24 7.23 7.22 7.21 7.21 7.21 7.19 7.19 7.18 6.93 6.91 6.89 6.85 6.84 6.84 6.83 6.82 6.82
4.44 4.43 4.42 4.41 4.40 4.39 4.38 4.38 4.37 4.36 4.27 4.26 4.25 4.24 4.23 4.22 4.21 4.20 4.17 4.15 4.15 4.14 4.13 4.12 4.11 4.09 1.53 1.52 1.42 1.41 1.39
0.00
0.89 1.00 2.06 5.23 1.04 1.08 2.06 1.07 1.14 3.11 3.11
10.5 10.0 9.5 9.0 8.5 8.0 7.5 7.0 6.5 6.0 5.5 5.0 4.5 4.0 3.5 3.0 2.5 2.0 1.5 1.0 0.5 0.0

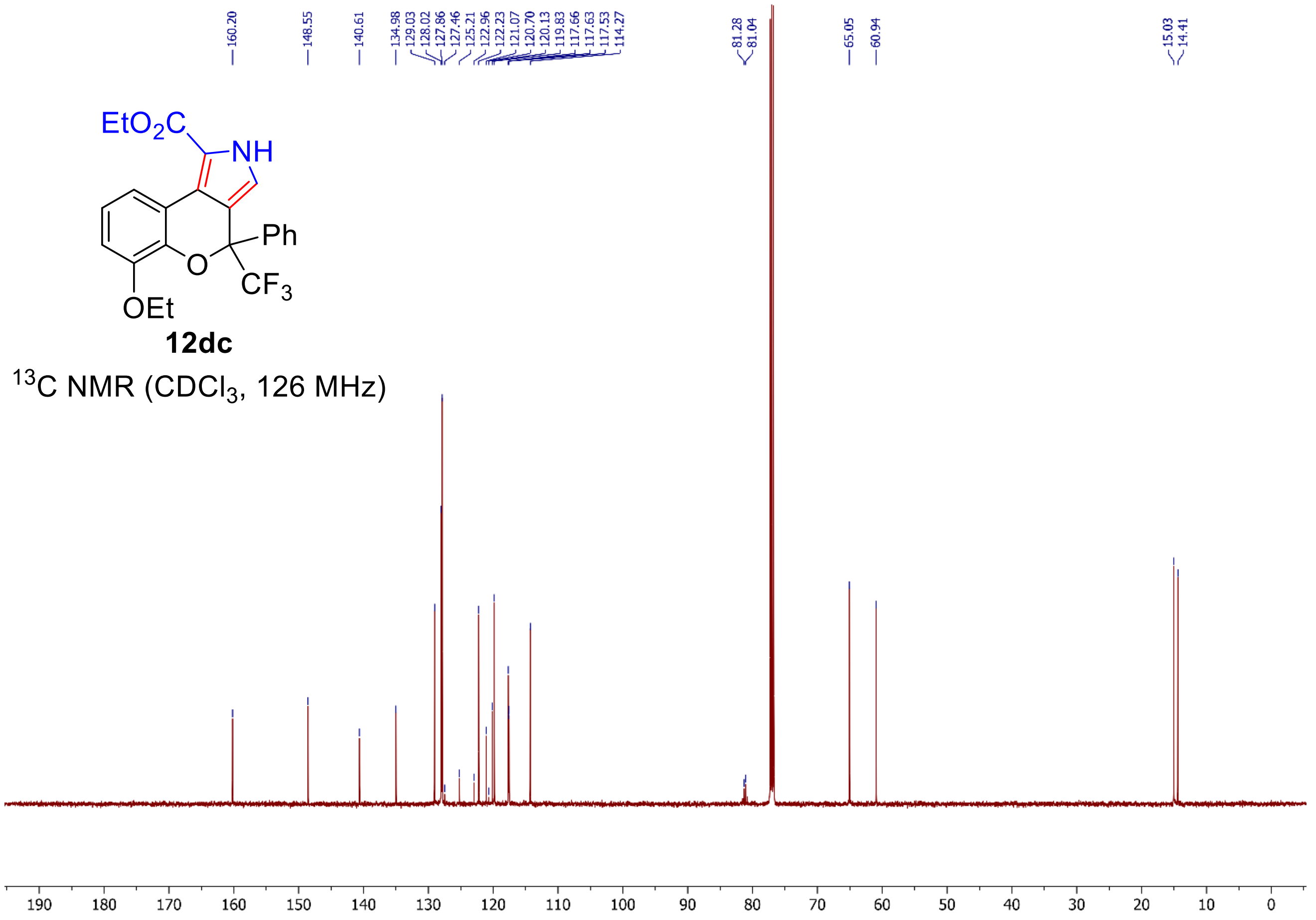
160.20
148.55
140.61
134.98 129.03 128.02 127.86 127.46 125.21 122.96 122.23 121.07 120.70 120.13 119.83 117.66 117.63 117.53 114.27
81.28 81.04
65.05
60.94
15.03 14.41
EtO2C
NH
Ph
O
CF3
OEt
12dc
13C NMR (CDCl3, 126 MHz)
190 180 170 160 150 140 130 120 110 100 90 80 70 60 50 40 30 20 10 0

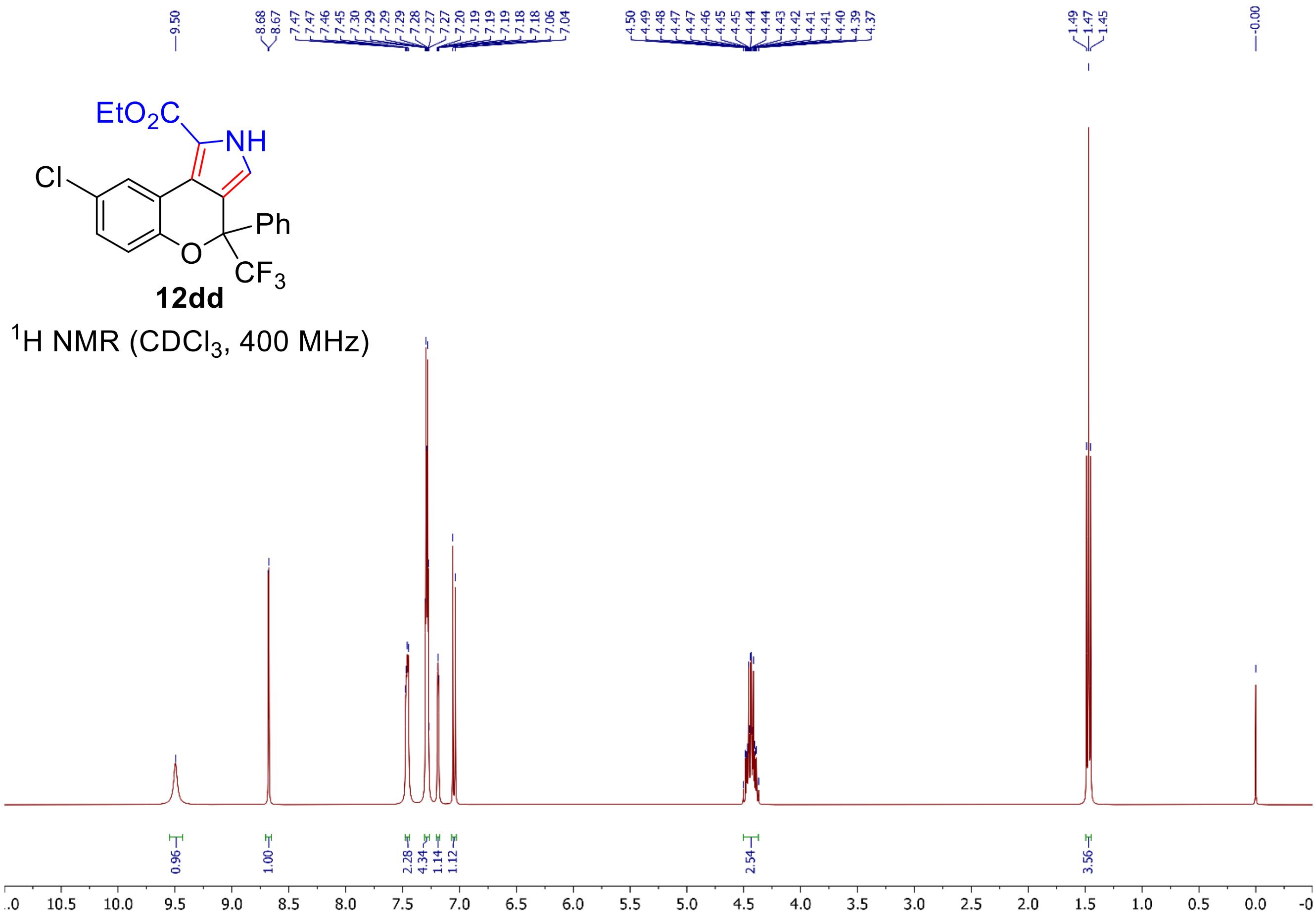
EtO2C
NH
Cl
Ph
O
CF3
12dd
1H NMR (CDCl3, 400 MHz)

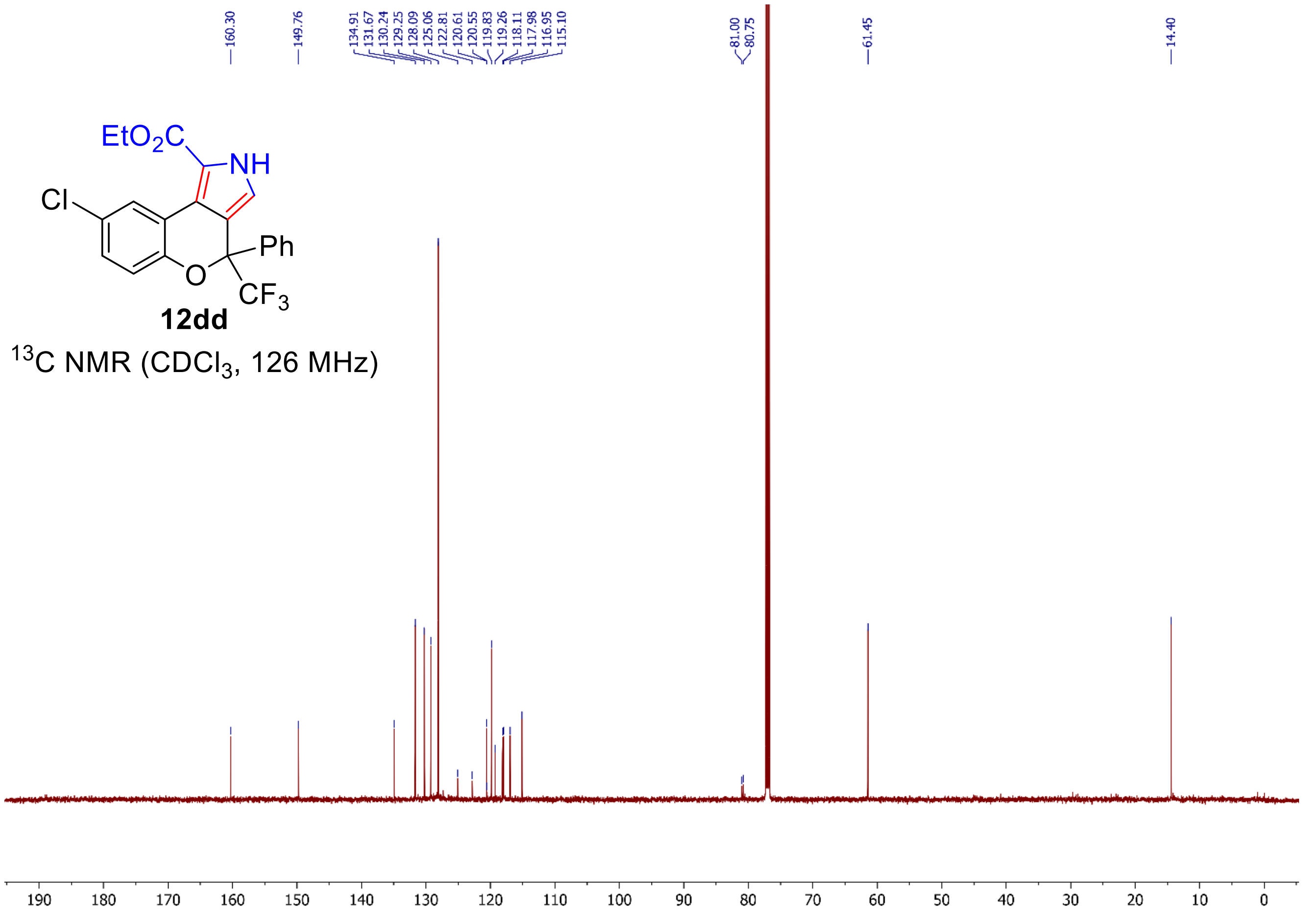
EtO2C
NH
Cl
Ph
O
CF3
12dd
13C NMR (CDCl3, 126 MHz)

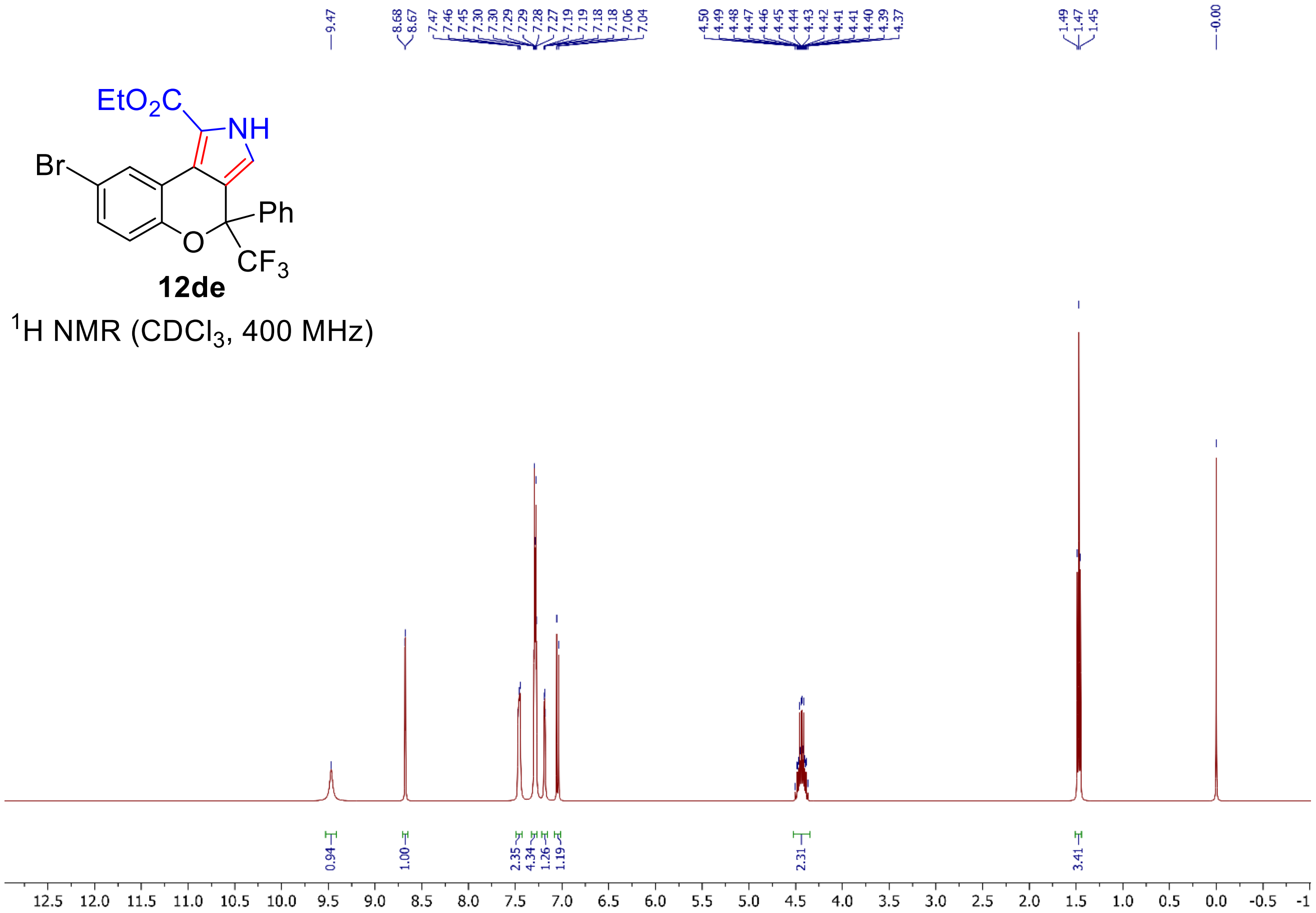

12de

^{1}H NMR ($CDCl_3$, 400 MHz)

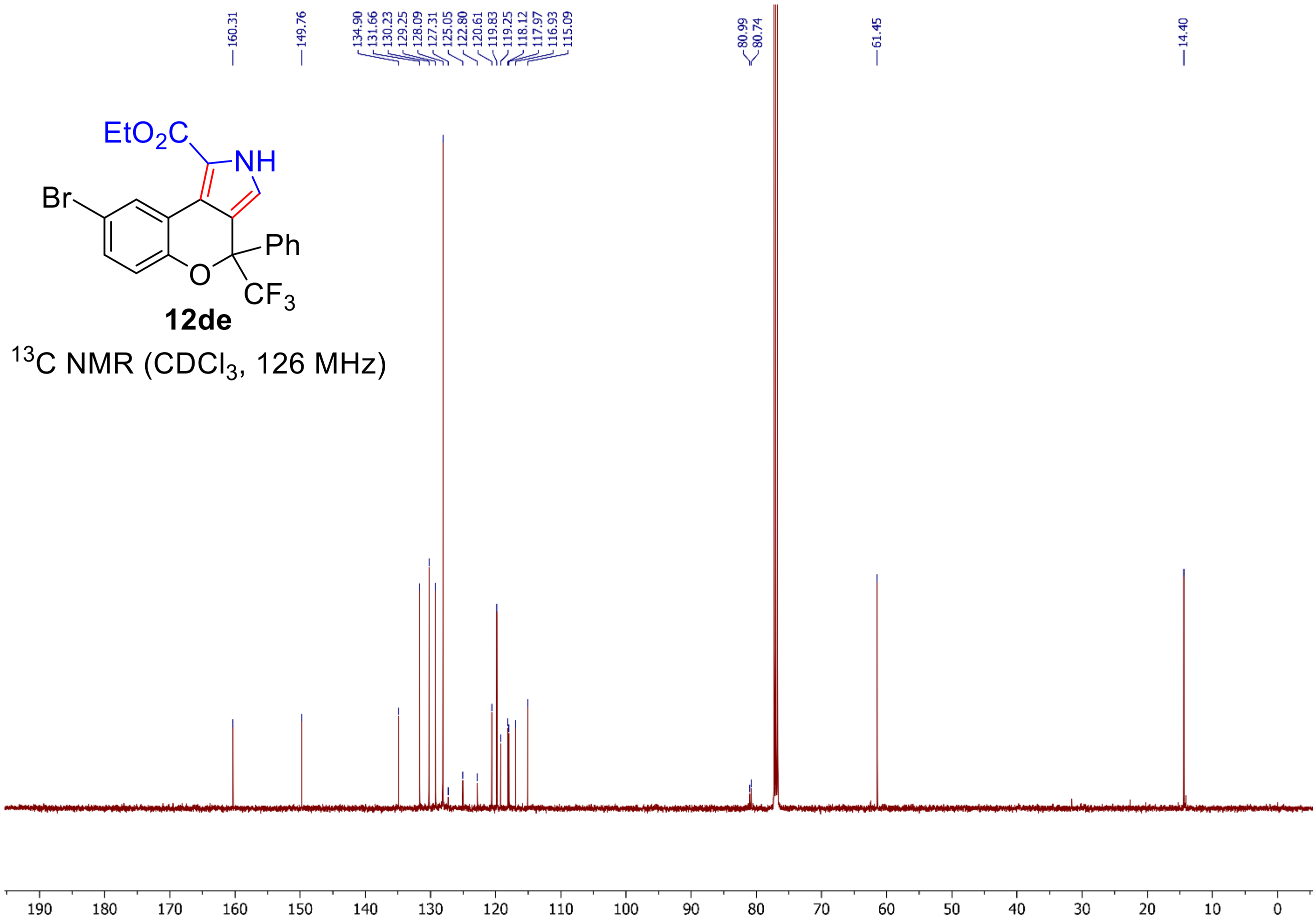

12de

^{13}C NMR ($CDCl_3$, 126 MHz)

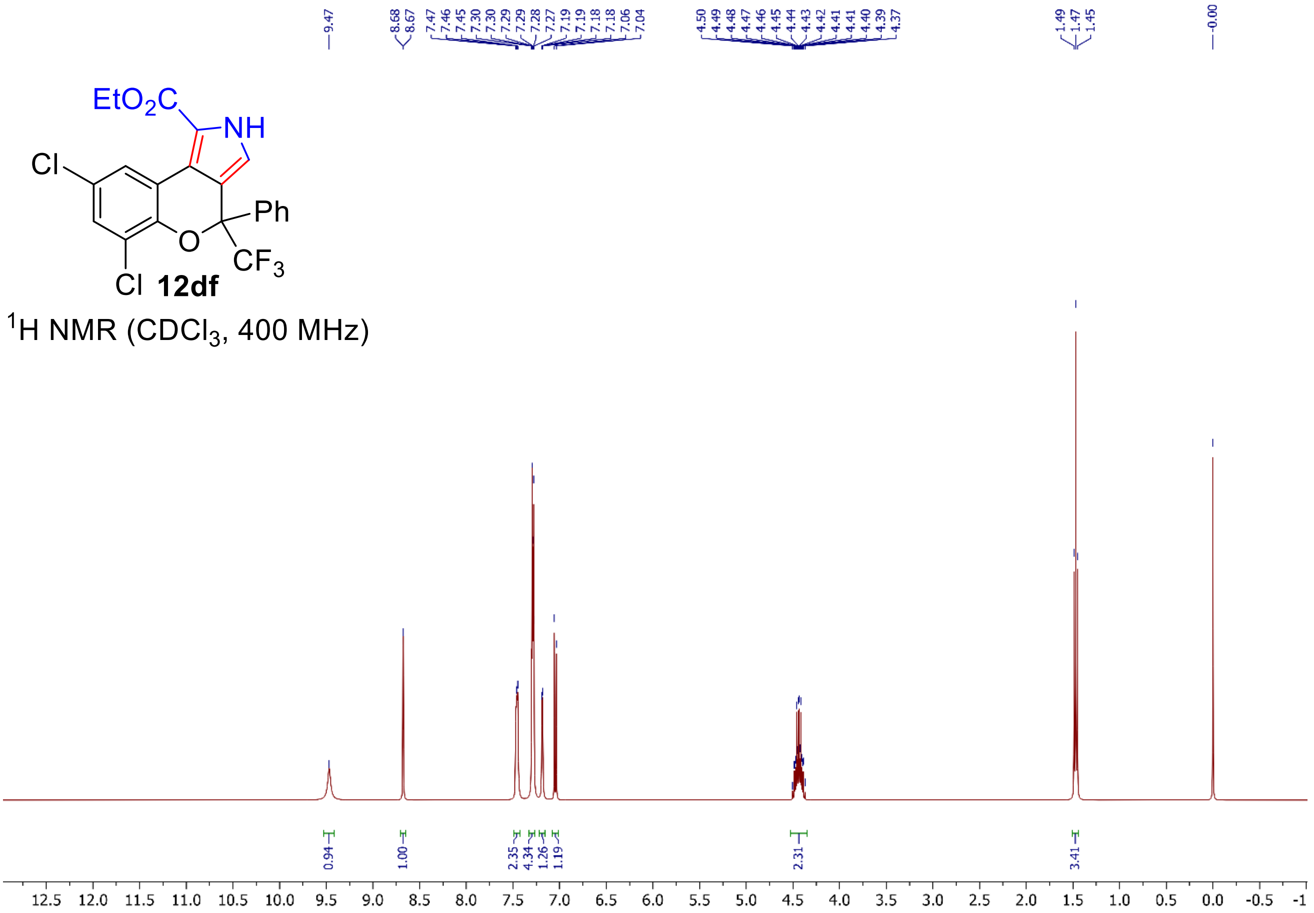

^{1}H NMR ($CDCl_3$, 400 MHz)

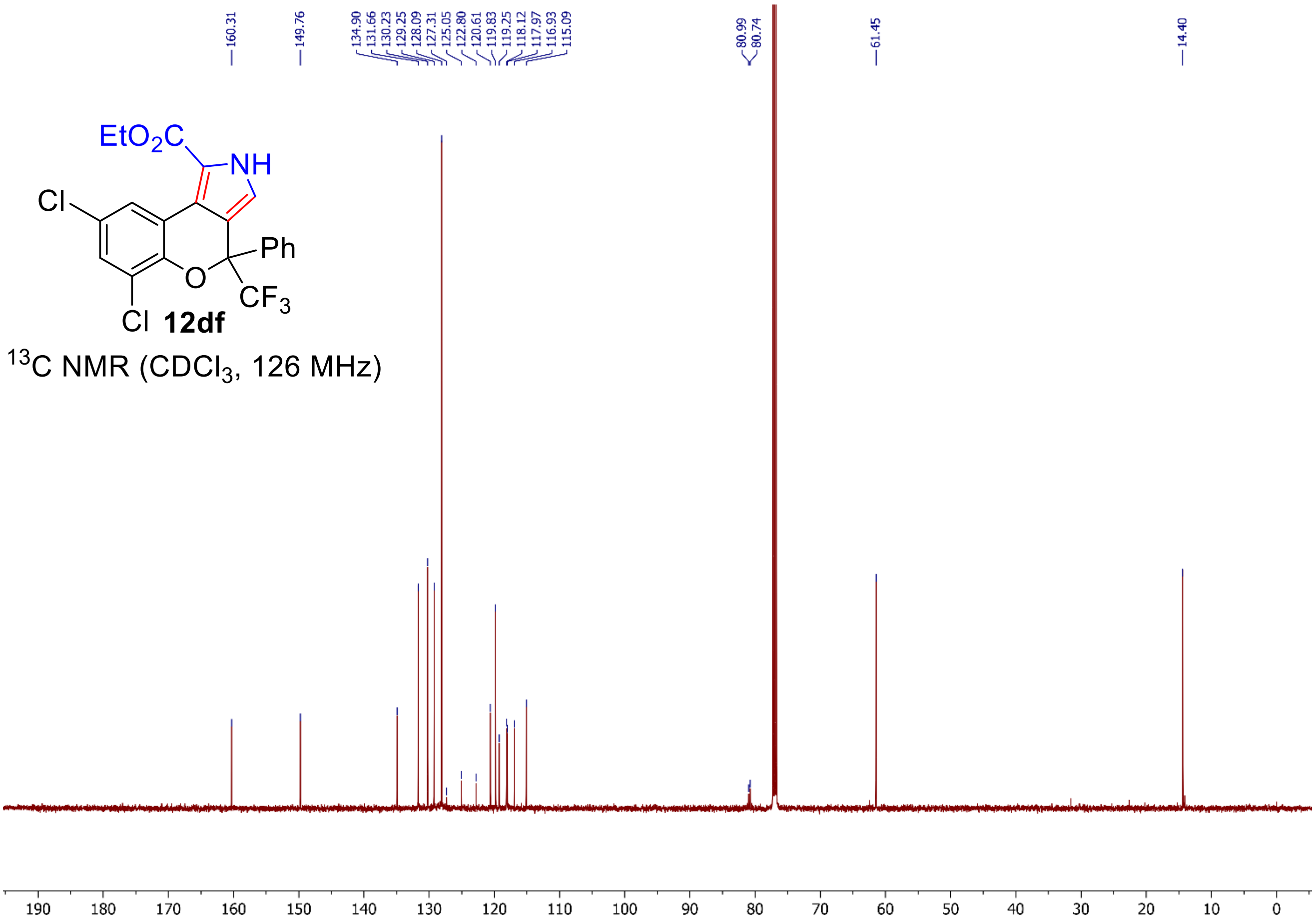

^{13}C NMR ($CDCl_3$, 126 MHz)

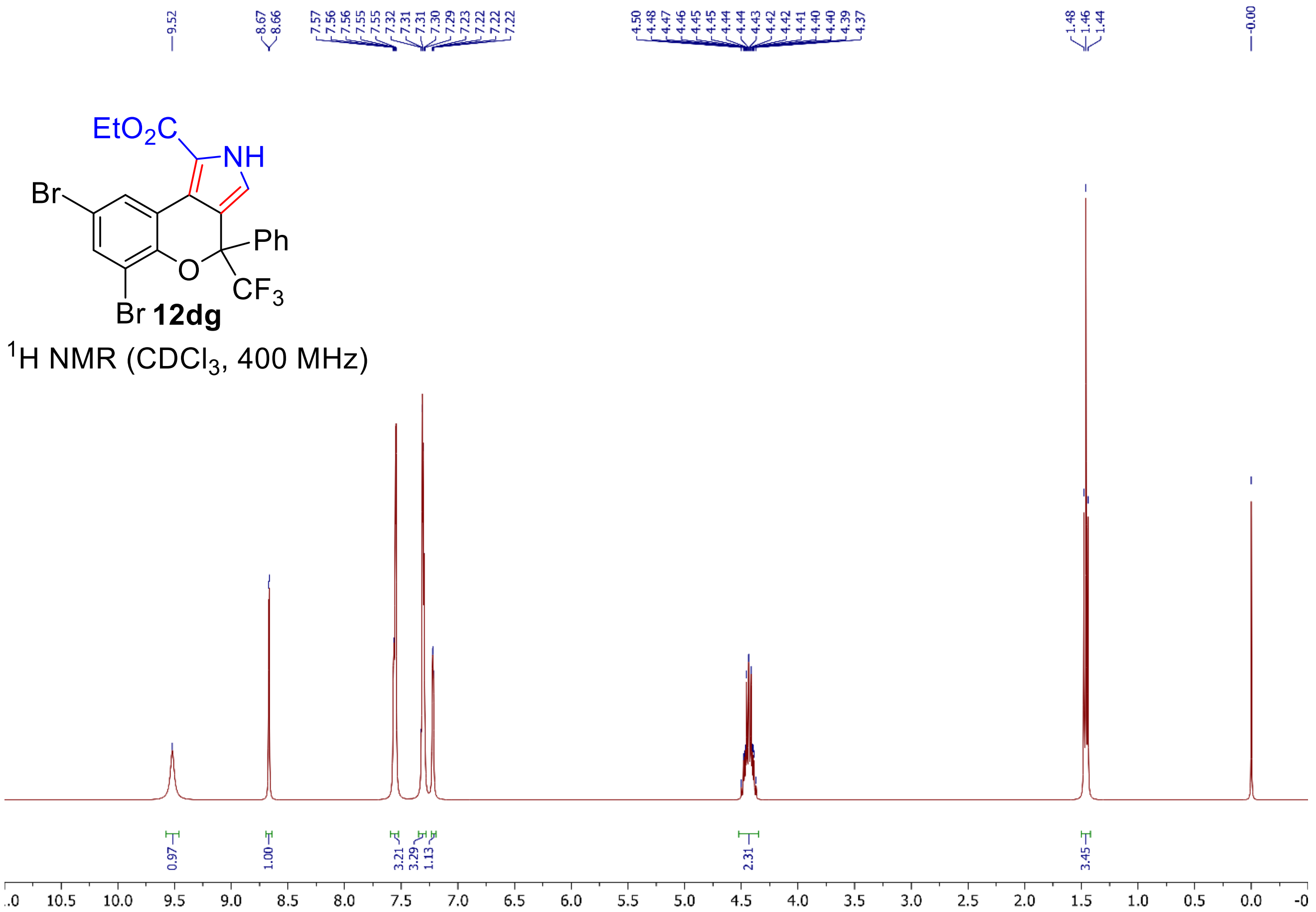

12dg

^{1}H NMR ($CDCl_3$, 400 MHz)

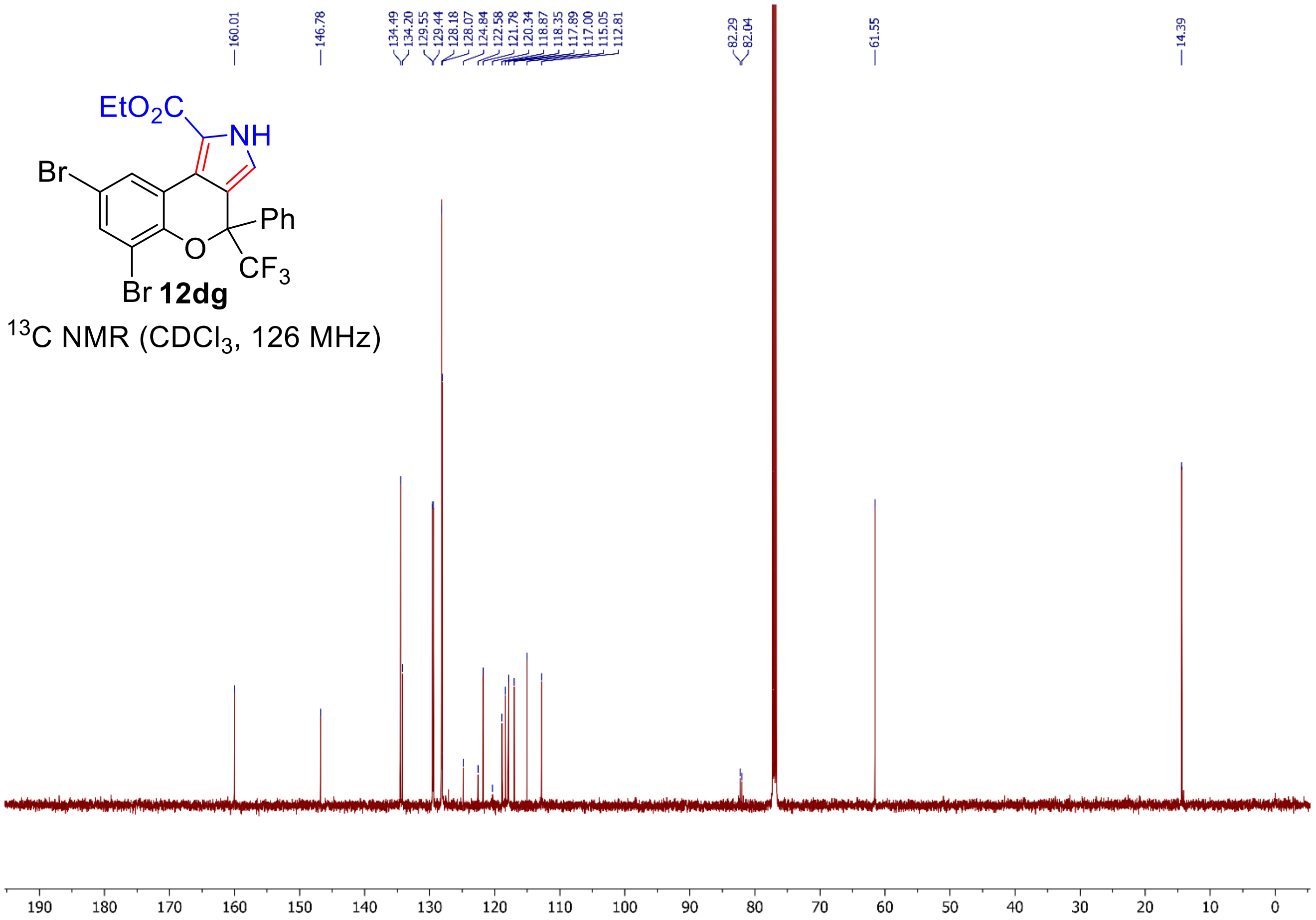

12dg

^{13}C NMR ($CDCl_3$, 126 MHz)

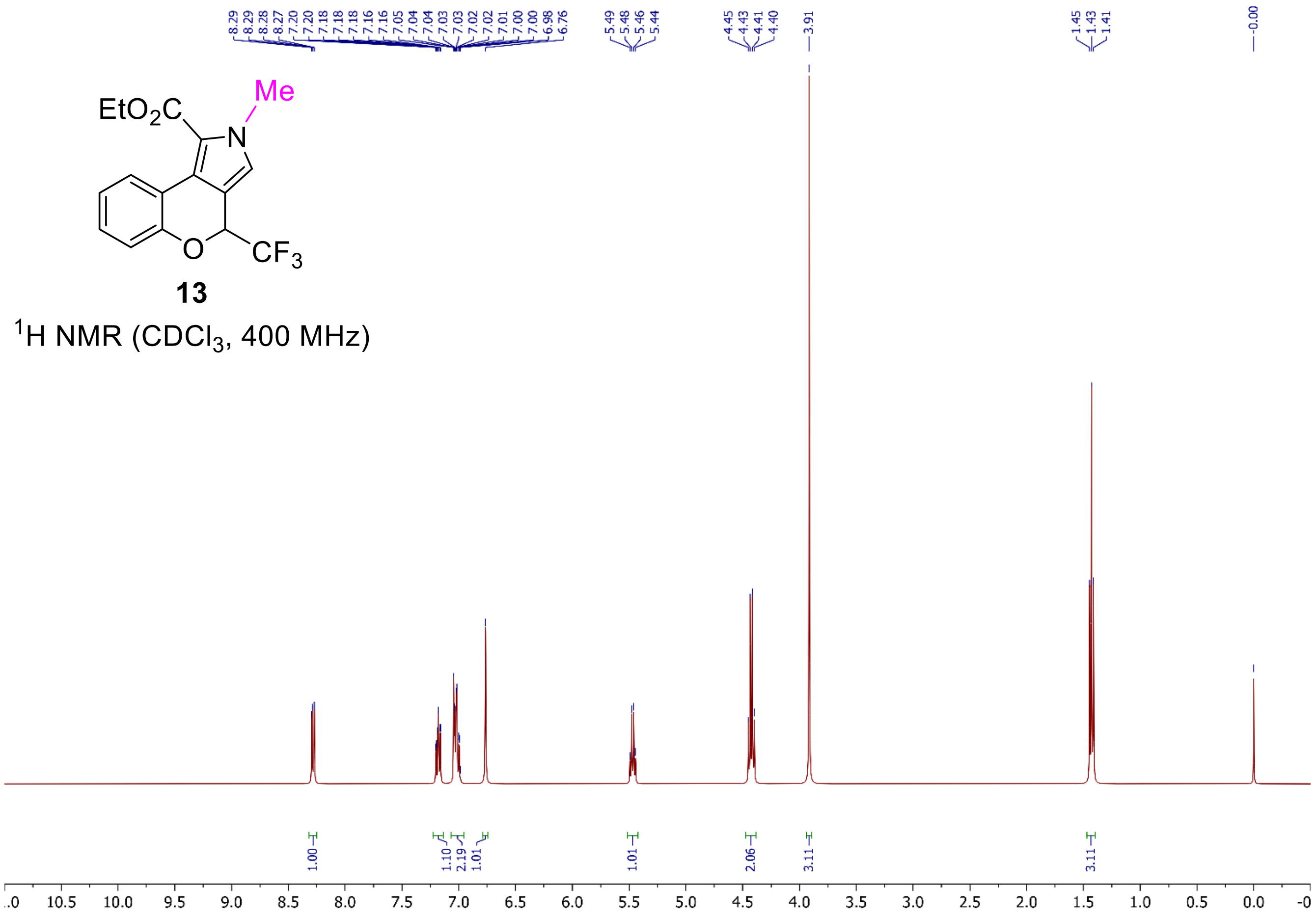

13

^{1}H NMR ($CDCl_3$, 400 MHz)

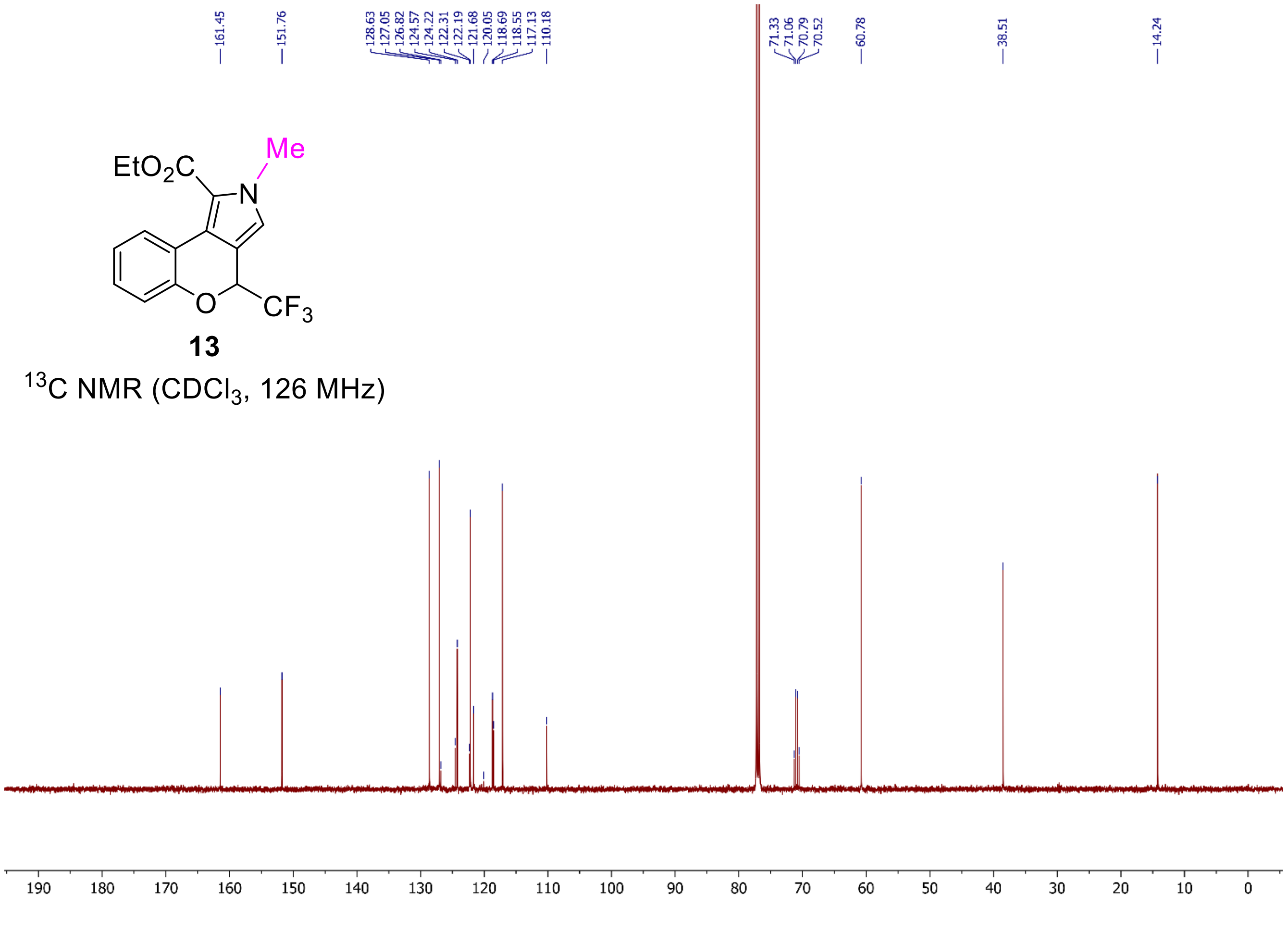

13

^{13}C NMR ($CDCl_3$, 126 MHz)

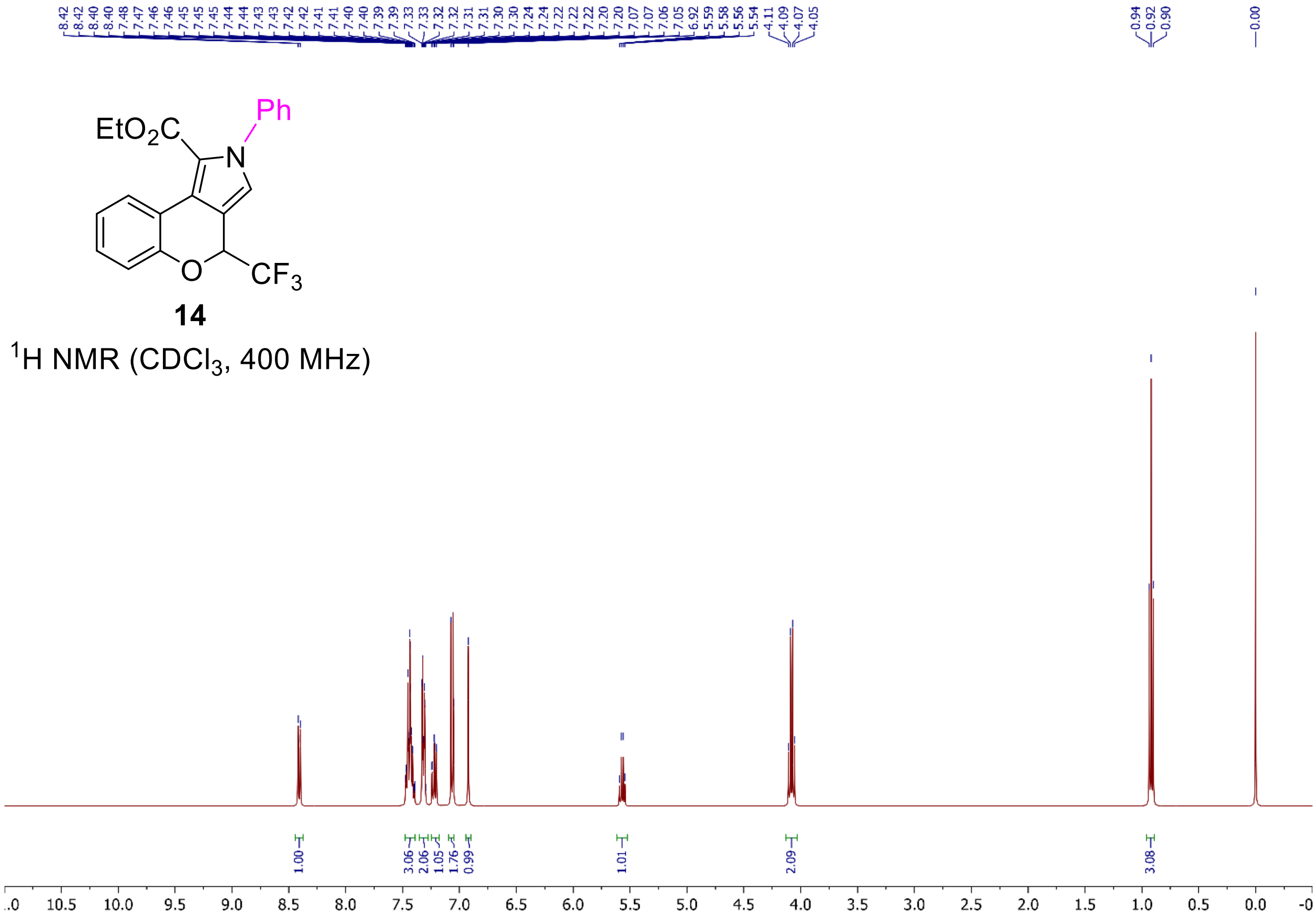

14

^{1}H NMR (CDCl$_3$, 400 MHz)

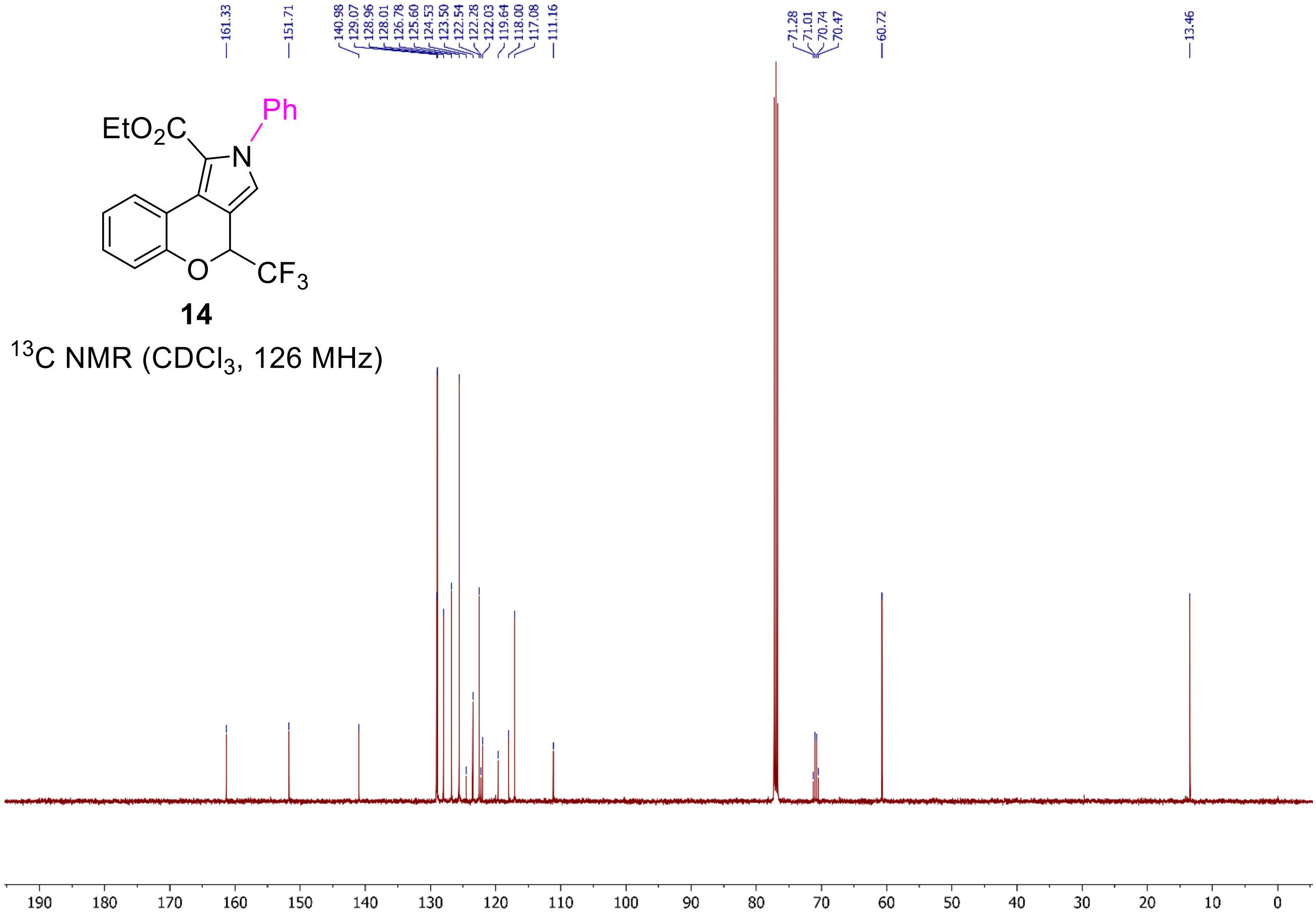

14

^{13}C NMR (CDCl$_3$, 126 MHz)

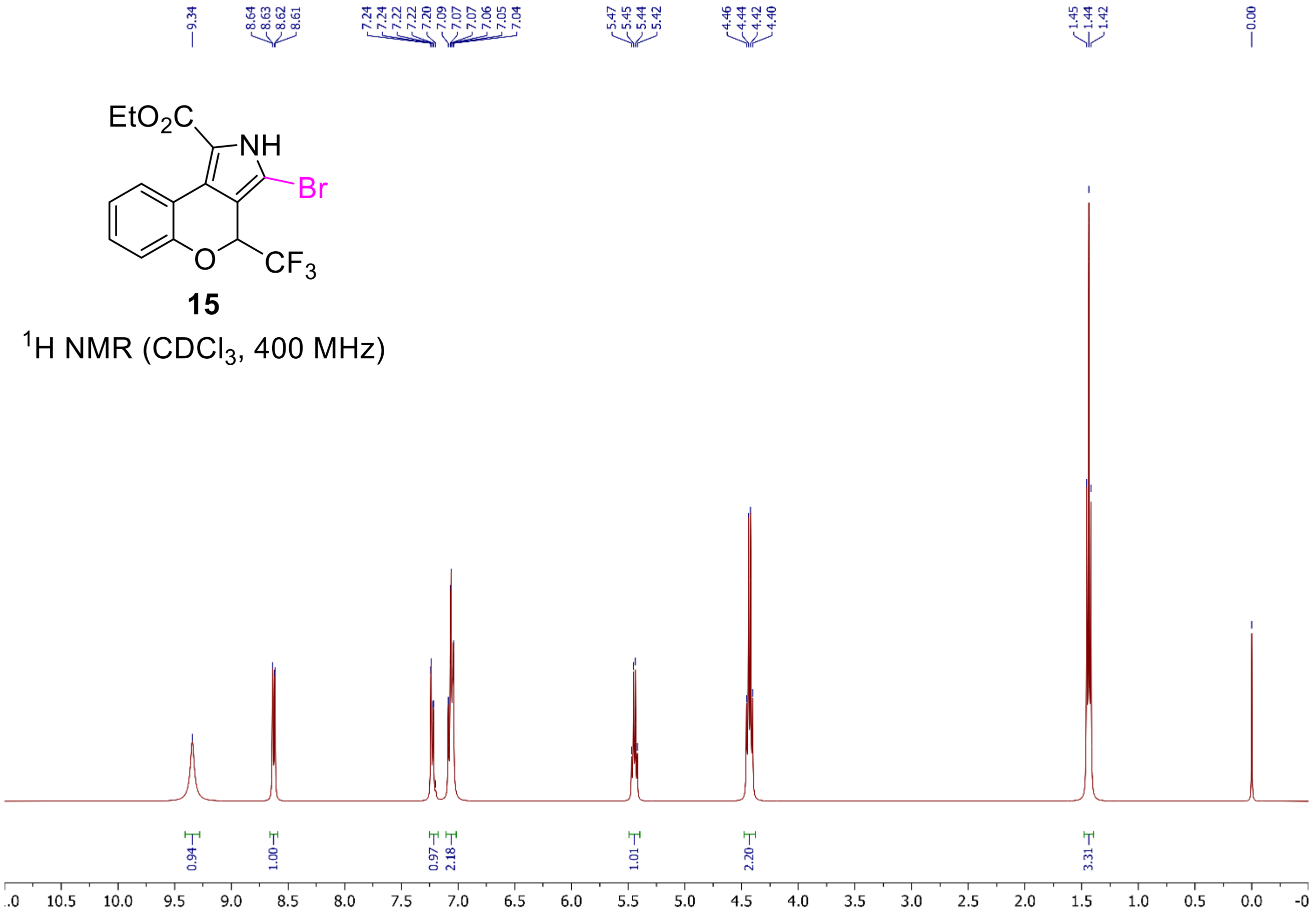
9.34
8.64 8.63 8.62 8.61
7.24 7.24 7.22 7.22 7.20 7.09 7.07 7.07 7.06 7.05 7.04
5.47 5.45 5.44 5.42
4.46 4.44 4.42 4.40
1.45 1.44 1.42
0.00
EtO2C
NH
Br
O
CF3
15
1H NMR (CDCl3, 400 MHz)
0.94 1.00 0.97 2.18 1.01 2.20 3.31

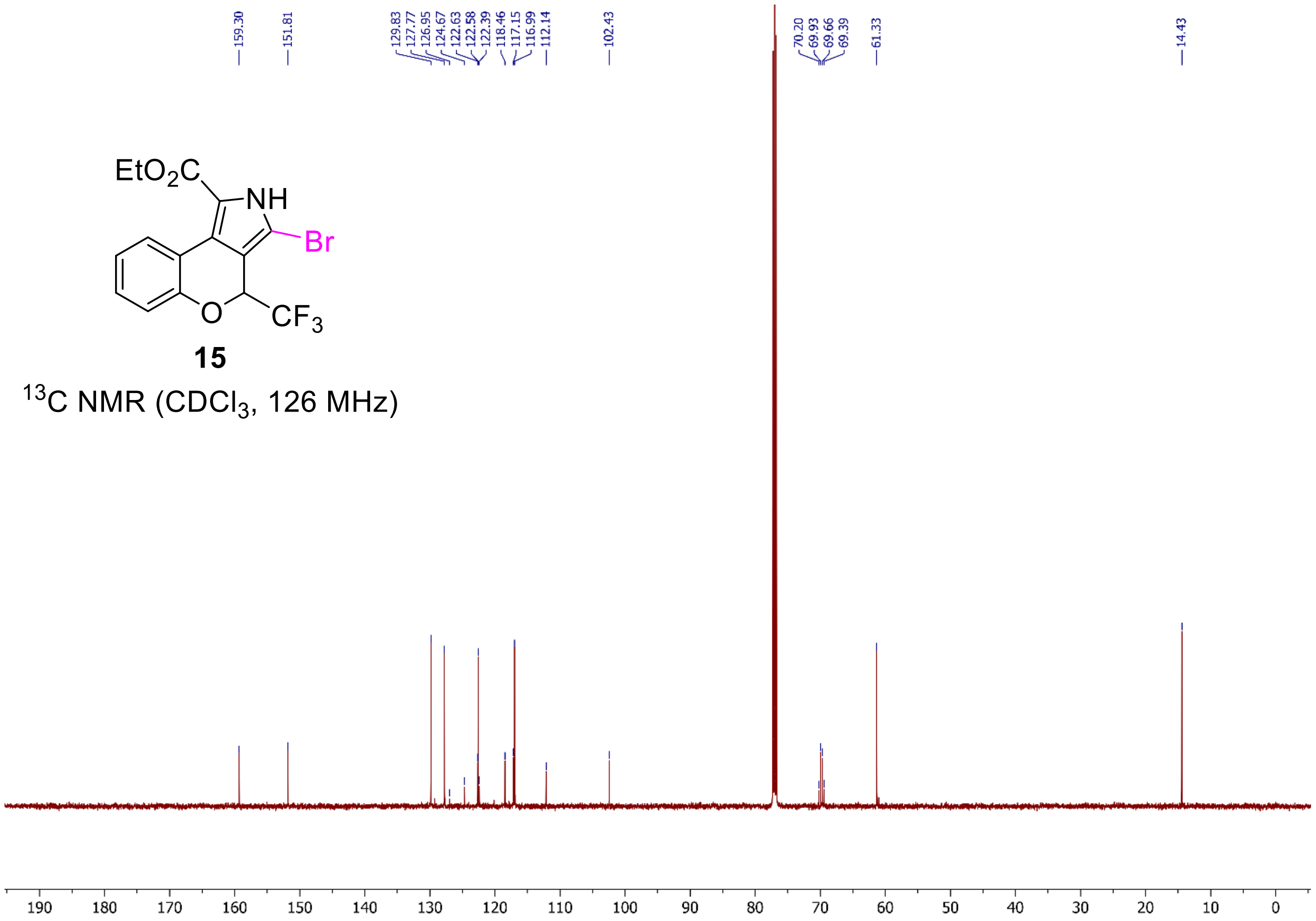
159.30
151.81
129.83 127.77 126.95 124.67 122.63 122.58 122.39 118.46 117.15 116.99 112.14
102.43
70.20 69.93 69.66 69.39
61.33
14.43
EtO2C
NH
Br
O
CF3
15
13C NMR (CDCl3, 126 MHz)
